# Ternifolipyrons A–J: new cytotoxic α-pyrones from *Isodon ternifolius* (D. Don) Kudô†

**DOI:** 10.1039/d3ra03146b

**Published:** 2023-06-29

**Authors:** Abdelsamed I. Elshamy, Tarik A. Mohamed, Ningombam Swapana, Yusuke Kasai, Masaaki Noji, Thomas Efferth, Hiroshi Imagawa, Mohamed-Elamir F. Hegazy, Akemi Umeyama

**Affiliations:** a Chemistry of Natural Compounds Department, National Research Centre 33 El Bohouth St., Dokki Giza 12622 Egypt elshamynrc@yahoo.com; b Chemistry of Medicinal Plants Department, National Research Centre 33 El-Bohouth St., Dokki Giza 12622 Egypt mohegazy@uni-mainz.de; c Faculty of Pharmaceutical Sciences, Tokushima Bunri University Yamashiro-cho Tokushima 770-8514 Japan umeyama@ph.bunri-u.ac.jp; d Department of Chemistry, Manipur Technical University Takyelpat Imphal 795004 Manipur India; e Department of Pharmaceutical Biology, Institute of Pharmaceutical and Biomedical Sciences, University of Mainz Staudinger Weg 5 55128 Mainz Germany

## Abstract

*Isodon ternifolius* (D.Don) Kudô is an important Asian herb used in traditional medicine against several diseases. Nineteen compounds were isolated from the dichloromethane–methanol (1 : 1) extract of *I. ternifolius* roots, including ten new α-pyrone derivatives, named ternifolipyrons A–J. The chemical structures of the isolates were determined by a combination of 1D and 2D NMR, along with LR- and HRMS spectroscopy. The absolute configurations of the α-pyrone derivatives were constructed based upon the X-ray signal crystal of the bromobenzoyl derivative of 1 as well as the electronic circular dichroism (ECD). All isolates (1–19) were investigated for their growth-inhibitory potential towards CCRF-CEM-leukemia cells at a fixed concentration of 30 μM. The compounds which exerted more than 50% inhibition at this concentration, compounds (7, 10, 12, 15–17), were tested at a different concentration range to determine their IC_50_ values in CCRF-CEM leukemia, MDA-MB-231 triple-negative breast cancer, and MCF7 breast cancer cell lines. Ursolic acid (16) showed the most potent activity against the three cancer cell lines with IC_50_ values of 8.37, 18.04, and 18.93 μM, respectively.

## Abstract 2

*Isodon ternifolius* (D.Don) Kudô is an important Asian herb used in traditional medicine against several diseases.

## Graphical abstract

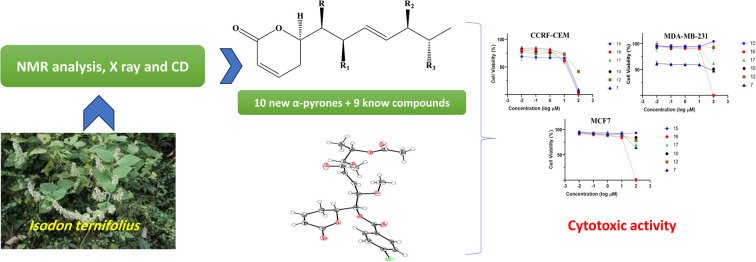

## 1. Introduction

The ≈150 species belonging to the *Isodon* genus are common in tropical and subtropical Asian areas.^1^ In traditional medicines, several *Isodon* species were medicinally used for the treatment of many microbial diseases, infections in the gastrointestinal and respiratory systems, tumors, inflammation, and hypertension.^1–3^ Recently, numerous clinical trial studies revealed the medicinal significance of these plants such as their anti-inflammatory, antimalarial, anti-enteritis, anti-jaundice, hepatoprotective effects; as well as the treatment of gastrointestinal ailments, arthralgia, hepatitis, and mastitis.^1,2,4^ Chemically, the *Isodon* species were documented to synthesize diverse diterpenes^1,5,6^ and lignans along with phenylethanoid glycosides.^2^

*I. ternifolius* (D.Don) Kudô is one of the important traditional herbal plant in traditional Chinese medicine against inflammation, icterohepatitis, enteritis, and diarrhoea^2^ alongside hepatitis and hepatitis B infection.^5^ Several unusual diterpenoids,^6–8^ lignans, phenylethanoid glycosides,^2^ triterpenes,^8^ sterols,^9^ spiroketones, and flavonoids^10^ were isolated and identified through chemical characterization of various extracts from distinct *I. ternifolius* parts. Because of the plant's historic significance and documented chemical variety, several biological actions of plant extracts and/or metabolites involving anti-cancer activity have been reported^6–8,10,11^ and the inhibition of DNA topoisomerase IB (TOP1) and tyrosyl-DNA phosphodiesterase 1 (TDP1).^8,9^ Longikaurin A from *I. ternifolius* exerted anticancer activity against several cancer cell lines, specifically hepatocellular carcinoma cells.^10^

The present investigation described (i) ten new α-pyrone derivatives isolated and identified from the roots of *I. ternifolius* along with other known compounds, (ii) the absolute configuration of the isolated compounds by NMR, X-ray signal crystal, and electronic circular dichroism (ECD), and (iii) the growth inhibition of these compounds towards CCRF-CEM leukemia, MDA-MB-23 triple-negative breast cancer, and MCF7 breast cancer cell lines.

## 2. Results and discussion

### 2.1. Structure elucidation of isolated compounds

**Fig. 1:**
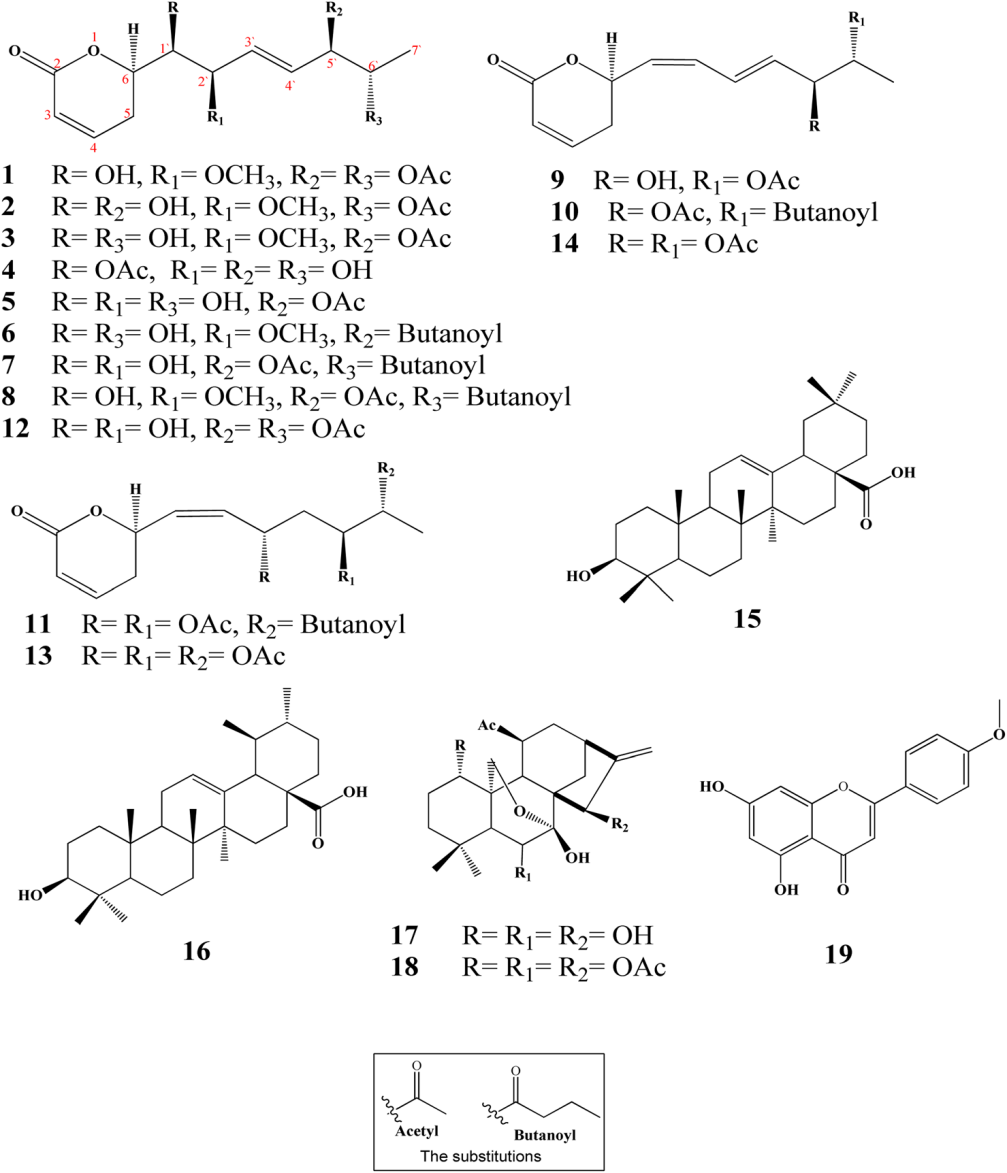
Chemical structures of isolated metabolites from *I. ternifolius* roots.

Ten new α-pyrones (2–11) along with further nine well-known compounds were isolated and identified from the dichloromethane–methanol (1 : 1) extract of the *I. ternifolius* roots *via* different chromatographic and spectroscopic tools (Fig. 1). The known metabolites were characterized as (6*R*,5′*R*,6′*S*,1′*R*,2′*R*)-6-[5′,6′-diacetyloxy-1′-hydroxy-2′-methoxy-3*E*-heptenyl]-5,6-dihydro-2*H*-pyran-2-one (1),^12^ synargentolide A (12),^13^ hyptolide (13),^14,15^ 6*R*-[5*R*,6*S*-diacetyloxy-1*Z*,3*E*-heptadienyl]-5,6-dihydro-2*H*-pyran-2-one (14),^16^ oleanolic acid (15),^17^ ursolic acid (16),^18,19^ sodoponin (18),^20^ rabdosianin B (17),^21,22^ and acacetin (19).^23^

**Fig. 2:**
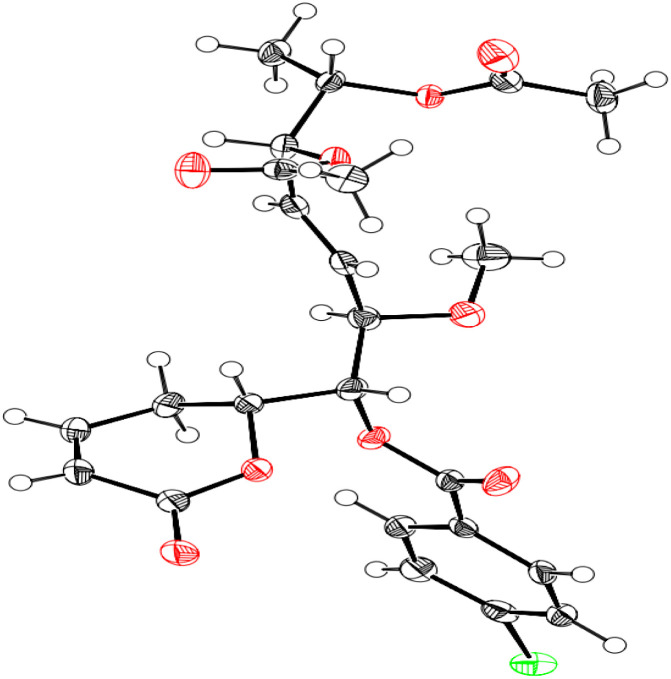
X-ray single crystal diffraction of the bromobenzoate derivative of 1 (S1).

Compound 1 was identified using mass spectroscopy with low and high resolution, as well as NMR analysis. Comparing with the reported data,^12^ the structure of 1 was confirmed as α-pyrone derivative, 6-[5′,6′-diacetyloxy-1′-hydroxy-2′-methoxy-3*E*-heptenyl]-5,6-dihydro-2*H*-pyran-2-one. The absolute configuration of 1 was constructed and confirmed by the X-ray single crystal diffraction of its bromobenzoate derivative (S1) (Fig. 2) and the positive ECD cotton effect at (Δ*ε*) 265.0 nm (+45.1). This detailed analysis allowed the unequivocal determination of 1 as (6*R*,5′*R*,6′*S*,1′*R*,2′*R*)-6-[5′,6′-diacetyloxy-1′-hydroxy-2′-methoxy-3*E*-heptenyl]-5,6-dihydro-2*H*-pyran-2-one that was isolated previously from *Hyptis oblongifolia* leaves.^12^

**Fig. 3:**
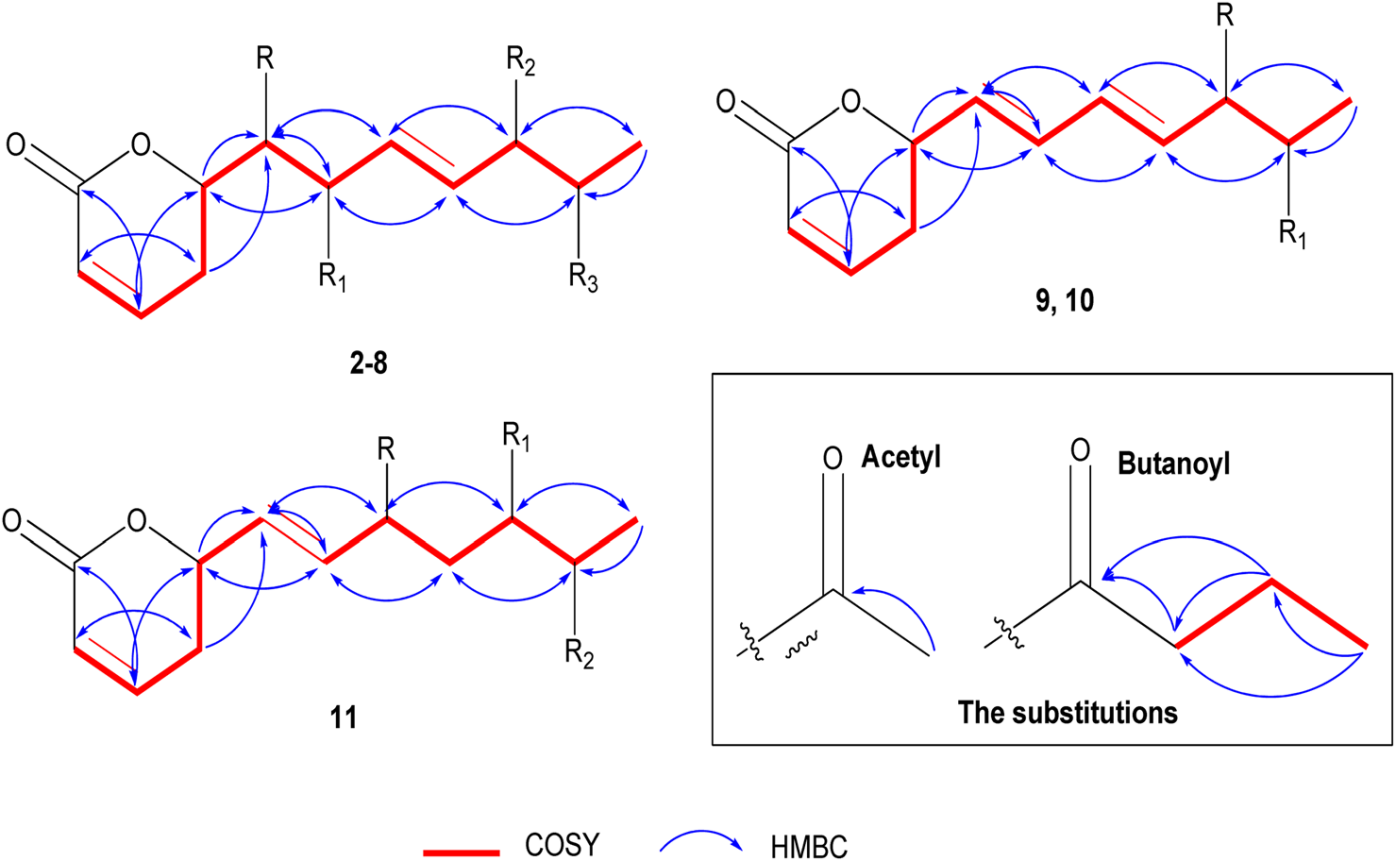
Significant ^1^H ^1^H COSY and HMBC of 2–11.

The positive mode HRCIMS of 2 exhibited a molecular ion peak *m*/*z* at 315.1453 that revealed a molecular formula of C_15_H_23_O_7_ (calc. 315.1444) and five unsaturation indexes. The presented ^1^H NMR data in Table 1 revealed the existence of five aliphatic oxygenated methene protons at *δ*_H_ 4.44 dt (*J* = 6.1, 3.8 Hz), 3.57 dd (*J* = 2.2, 6.1 Hz), 3.72 dd (*J* = 4.0, 7.6 Hz), 4.05 t (*J* = 5.0 Hz), and 4.79 m, four olefinic methene protons at *δ*_H_ 5.88 dt (*J* = 1.6, 9.9 Hz), 6.97 dd (*J* = 5.7, 9.7 Hz), 5.66 dd (*J* = 7.7, 15.7 Hz), and 5.73 dd (*J* = 5.9, 15.8 Hz), one methylene at *δ*_H_ 2.45 m and one methyl protons at *δ*_H_ 1.11 d (*J* = 6.5 Hz). Also, two protons characteristic for two methyl groups were assigned in oxygenated systems, including methoxy and acetoxy groups, at respective *δ*_H_ 3.22 s and 1.93 s. Totally, 15 carbon resonances were characterized based on the ^13^C NMR data (Table 2) and classified by the DEPT-135 and HSQC experiments. The careful assignments of these analyses yielded two quaternary carbons characteristic for two carbonyls at *δ*_C_ 164.9 (carbonyl of δ-lactone moiety) and *δ*_C_ 171.0 (acetoxyl carbonyl group), four olefinic methenes at *δ*_C_ (119.7, 129.1, 147.1, and 133.7), five oxygenated methenes at *δ*_C_ (73.0, 73.2, 74.3, 77.5, and 80.6), one aliphatic methylene at *δ*_C_ 24.7, one, and one methyl proton at *δ*_C_ 14.1, one methyl for acetoxy at *δ*_C_ 19.7, and one methyl of methoxy group at *δ*_C_ 55.6. All these data revealed that 2 has the same structure as 1 (ref. 12) except for the presence of only one acetoxyl substituent alongside two hydroxyl groups. The acetoxyl group was located in C-6′ depending upon the ^1^H ^1^H COSY correlations (Fig. 3) of the olefinic proton H-4′ at *δ*_H_ 5.73 dd (*J* = 5.9, 15.8 Hz) and the hydroxylated proton (H5′) at *δ*_H_ 4.05 t (*J* = 5.0 Hz), H-5′/H-6′ at *δ*_H_ 4.79 m, and H-6′ and methyl proton (H-7′) at *δ*_H_ 1.11 d (*J* = 6.5 Hz). The ^3^*J* HMBC correlations (Fig. 3) between the H-4′/C-6′ (*δ*_C_ 73.0), H-6′/Ac-CO (*δ*_C_ 171.0), H-7′/C-5′ (*δ*_C_ 73.2), H-5 (*δ*_H_ 2.45 m)/C-1′ (74.3), and H-3′(*δ*_H_ 5.66 dd (*J* = 7.7, 15.7 Hz))/C-1′ confirmed the localization of the acetoxyl group in C-6′ and the two hydroxyl groups in C-1′ and C-5′. Based upon these 1D and 2D NMR analyses, the structure of 2 was deduced as 6-[6′-acetyloxy-1′,5′-dihydroxy-2′-methoxy-3-heptenyl]-5,6-dihydro-2*H*-pyran-2-one. The *trans* (*E*) geometry of the C-3′/C-4′ olefinic system was confirmed by the discernible coupling constants of both sets of olefinic protons at 15.7 Hz.^12,13^ Comparing with 1 and the literature,^12^ the absolute orientation of 2 was affirmed *via* the coupling constants of the chiral carbons (Rahman and Gibbons, 2015 ^16^) and the positive ECD cotton effect at (Δ*ε*) 259.2 nm (+109.9). Thus, 2 was elucidated as (6*R*,5′*R*,6′*S*,1′*R*,2′*R*)-6-[6′-acetyloxy-1′,5′-dihydroxy-2′-methoxy-3*E*-heptenyl]-5,6-dihydro-2*H*-pyran-2-one (ternifolipyron A).


^1^H NMR (500 Hz) spectral data of 2–8^a^

**Table:** 

No.	2	3	4	5	6	7	8
1	—	—	—	—	—	—	—
2	—	—	—	—	—	—	—
3	5.88 dt (1.6, 9.9)	5.87 dt (1.9, 9.8)	5.87 dt (1.9, 11.7)	5.88 dt (2.1, 9.9)	5.88 dt (1.7, 11.6)	5.87 ddd (1.2, 4.0, 8.6)	5.99 dt (2.0, 9.9)
4	6.97 dd (5.7, 9.7)	6.96 dt (4.4, 9.7)	6.98 dt (4.1, 13.9)	6.97 dd (3.2, 9.6)	6.96 dd (4.9, 9.7)	6.98 dddd (2.9,5.6, 9.7)	7.09 dddd (4.2, 8.6, 10.0)
5	2.45 m	2.44 dddd (1.9, 4.2. 9.3)	2.47 m	2.47 m	2.44 m	2.46 m	2.57 m
6	4.44 dt (6.1, 3.8)	4.42 m	4.46 m	4.45 dd (5.5, 2.6)	4.43 m	4.44 t (2.6)	4.56 ddd (9.0, 9.0, 6.4)
1′	3.57 dd (2.2, 6.1)	3.58 dd (4.3, 13.3)	3.55 dd (3.7, 6.3)	3.57 dd (3.7, 6.4)	3.58 t (3.8)	3.55 dd (3.4, 6.4)	3.68 dd (3.8, 6.4)
2′	3.72 dd (4.0, 7.6)	3.72 dd (3.8, 7.6)	4.18 dd (3.8, 5.9)	4.18 d (3.8, 5.7)	3.71 dd (4.0, 7.9)	4.22 brt (4.2)	3.89 m
3′	5.66 dd (7.7, 15.7)	5.64 dd (7.5, 15.8)	5.80 dd (6.2, 15.7)	5.77 dd (6.1, 15.9)	5.64 dd (7.9, 15.8)	5.84 dddd (1.0, 5.9 15.8)	5.86 dd (7.4, 15.8)
4′	5.73 dd (5.9, 15.8)	5.72 dd (6.6, 15.8)	5.71 dd (6.3, 15.6)	5.73 dd (6.2, 13.3)	5.73 dd (6.8, 15.8)	5.69 dddd (1.3, 6.9, 15.8)	5.85 dd (6.0, 15.8)
5′	4.05 t (5.0)	5.06 dd (4.0, 6.5)	4.03 t (5.5)	5.08 dd (4.1, 6.5)	5.07 ddd (0.7, 4.2, 10.4)	5.30 dd (3.4, 7.0)	5.15 dddd (3.6, 6.6, 13.2)
6′	4.79 m	3.78 dd (4.0, 6.5)	4.78 m	3.76 dd (4.1, 6.4)	3.76 dd (4.5, 6.6)	4.99 dddd (3.4, 6.6, 13.2)	5.41 ddd (3.5, 5.0)
7′	1.11 d (6.5)	1.06 d (6.5)	1.08 d (6.5)	1.05 d (6.5)	1.06 d (6.5)	1.11 d (6.6)	1.25 d (6.6)
2′-OMe	3.22 s	3.19 s	—	—	3.21 s	—	3.33 br s

a All the compounds were measured in CD_3_OD at 500 MHz; the coupling constants (*J* in Hz) are given in parentheses.

**Table:** 

6′-Ac		5′-Ac		1′-Ac		5′-Ac		6′-But		5′-Ac		5′-Ac	
CO	1.93 s	CO	1.99 s	CO	1.99 s	CO	1.98 s	CO	—	CO	—	CO	—
CH_3_	—	CH_3_	—	CH_3_	—	CH_3_	—	CH_2_	2.26 t (7.4)	CH_3_	1.95 s	CH_3_	2.09 s
								CH_2_	1.55 m	6′-But		6′-But	
								CH_3_	0.86 t (7.4)	CO	—	CO	—
										CH_2_	2.26 t	CH_2_	2.31 t (7.3)
										CH_2_	1.55 m	CH_2_	1.65 m
										CH_3_	0.86 t (7.4)	CH_3_	0.97 t (7.4)


^13^C NMR (125 Hz) spectral data of 2–8

**Table:** 

No.	2	3	4	5	6	7	8
1	—	—	—	—	—	—	—
2	164.9	164.9	165.0	165.0	164.9	165.0	164.8
3	119.7	119.7	119.7	119.7	119.7	119.8	119.8
4	147.1	147.1	147.2	147.2	147.1	147.2	147.1
5	24.7	24.6	24.9	24.9	24.6	25.1	24.9
6	77.5	77.5	77.6	77.6	77.5	77.5	77.4
1′	74.3	74.1	74.6	74.4	74.1	74.4	74.1
2′	80.6	80.7	70.4	70.4	80.7	70.1	80.4
3′	129.1	131.0	132.6	134.2	131.0	135.3	131.7
4′	133.7	129.8	130.3	126.5	130.1	125.1	128.5
5′	73.2	77.7	73.4	77.8	77.5	75.0	70.1
6′	73.0	68.1	73.1	68.3	68.2	70.4	74.7
7′	14.1	17.1	13.9	17.1	17.2	13.9	14.1
2′-OMe	55.6	55.6	—	—	55.6	—	55.8

**Table:** 

6′-Ac		5′-Ac		1′-Ac		5′-Ac		6′-But		5′-Ac		5′-Ac	
CO	19.7	CO	19.6	CO	19.8	CO	19.7	CO	173.1	CO	170.4	CO	170.3
CH_3_	171.0	CH_3_	170.7	CH_3_	171.1	CH_3_	170.8	CH_2_	35.7	CH_3_	19.5	CH_3_	19.6
								CH_2_	18.1	6′-But		6′-But	
								CH_3_	12.5	CO	173.2	CO	173.1
										CH_2_	35.7	CH_2_	35.7
										CH_2_	18.1	CH_2_	18.1
										CH_3_	12.5	CH_3_	12.6

The positive mode HRCIMS of 3 showed a molecular ion peak *m*/*z* at 315.1453, indicating a molecular formula of C_15_H_23_O_7_ (calc. 315.1444) with five unsaturation indexes. The assigned 1D NMR, including ^1^H (Table 1) and ^13^C NMR (Table 2) data, revealed that 3 had the same structure as 2, with differences in the localization of the groups that were deduced *via* the variation of some protons and carbons. These variations were clearly observed in the downfield shift of H-5′/C-5′ by 1.01/4.5 ppm at *δ*_H_ 5.06 dd (*J* = 4.0, 6.5 Hz)/*δ*_C_ 77.7, the upfield shift of H-6′/C-5′ by 1.01/4.9 ppm at *δ*_H_ 3.78 dd (*J* = 4.0, 6.5 Hz)/*δ*_C_ 68.1 and C-7′ by 3.0 ppm at *δ*_C_ 17.1 indicating the presence of the acetoxyl group in C-6′ and the hydroxyl group in C-5′. These localizations were confirmed *via*^1^H ^1^H COSY correlations (Fig. 3) of H-4′ (*δ*_H_ 5.72 dd (*J* = 6.6, 15.8 Hz))/H-5′ (*δ*_H_ 5.06 dd (*J* = 4.0, 6.5 Hz)), H-5′/H-6′ (*δ*_H_ 3.78 dd (*J* = 4.0, 6.5 Hz)), and H-6′/H-7′ (*δ*_H_ 1.06 d (*J* = 6.5 Hz)) along with the ^3^*J* HMBC (Fig. 3) correlations of H-5′/Ac-CO (*δ*_C_ 170.7) and H-7′/C-5′ (*δ*_C_ 77.7). From above, 3 was constructed as 6-[5′-acetyloxy-1′,6′-dihydroxy-2′-methoxy-3-heptenyl]-5,6-dihydro-2*H*-pyran-2-one. As above compounds, the large coupling constants of the C-3′/C-4′ olefinic system sets at 15.8 Hz allowed its unequivocal geometry as *trans* (*E*).^12,13^ The determination of the absolute configuration of 3 was carried out depending upon the comparing of the chiral carbons′ coupling constants with the literature^16^ along with the positive ECD cotton effect at (Δ*ε*) 261.8 nm (+83.4).^16^ So, 3 was predicted as (6*R*,5′*R*,6′*S*,1′*R*,2′*R*)-6-[5′-acetyloxy-1′,6′-dihydroxy-2′-methoxy-3-heptenyl]-5,6-dihydro-2*H*-pyran-2-one (ternifolipyron B).

The HRCIMS of 4 in a positive mode exhibited a molecular ion peak *m*/*z* at 301.1265, confirming the molecular formula of C_14_H_21_O_7_ (calc. 301.1209) and four unsaturation indexes. The overall data presented in 1D NMR (Tables 1 and 2) deduced that 4 was very close to the structure of 3 with some clear variations in the functional groups in the long chain. These significances were (i) the presence of three free hydroxy groups at C-2′, C5′, and C-6′ at *δ*_H_/*δ*_C_ 4.18 dd (*J* = 3.8, 5.9 Hz)/70.4, 4.03 t (*J* = 5.5 Hz)/73.4 and 4.78 m/73.1, (ii) the absence of the methoxyl group that deduced *via* the downfield shift of H-2′ by 0.46 at *δ*_H_ 4.18 dd (*J* = 3.8, 5.9 Hz) and upfield of C-2′ by 9.8 ppm at *δ*_C_ 70.4, (iii) the upfield shift of the methyl proton, H-7′, by 3.1 ppm at *δ*_H_ 13.9, indicating that the only acetoxyl group was located in another carbon except C-6′. The localization of the acetoxyl group was affirmed in C-1′ *via* the ^1^H ^1^H COSY correlations of H-6 (*δ*_H_ 4.46 m)/H-1′ (*δ*_H_ 3.55 dd (*J* = 3.7, 6.3 Hz), H-1′/H-2′ (*δ*_H_ 4.18 dd (*J* = 3.8, 5.9 Hz) and H-2′/H-3′ (*δ*_H_ 5.80 dd (*J* = 6.2, 15.7 Hz) along with the ^3^*J* HMBC correlations of H-1′/Ac-CO (*δ*_C_ 171.1), H-1′/C–H-5 (*δ*_C_ 24.9) and H-1′/C-3′ (*δ*_C_ 132.6) (Fig. 3). Hence, the structure of 4 was predicted as 6-[1′-acetyloxy-2′,5′,6′-trihydroxy-3-heptenyl]-5,6-dihydro-2*H*-pyran-2-one. As described for the above compounds, the H-3′/H-4′ olefinic system sets coupling constant at 15.7 Hz confirmed its *trans* (*E*) geometry.^13,16^ Similar to above compounds, the absolute configuration of 4 was established based upon the positive ECD cotton effect at (Δ*ε*) 258.0 nm (+46.2).^12^ Thus, 4 was assigned as (6*R*,5′*R*,6′*S*,1′*R*,2′*R*)-6-[1′-acetyloxy-2′,5′,6′-trihydroxy-3-heptenyl]-5,6-dihydro-2*H*-pyran-2-one (ternifolipyron C).

Based upon the positive mode HRCIMS of 5, which exhibited a molecular ion peak *m*/*z* at 301.1269, indicating a molecular formula of C_14_H_21_O_7_ (calcd 301.1287) and four unsaturation indexes. By assignment of the ^1^H (Table 1) and ^13^C (Table 2) NMR data, 5 was affirmed to have the structure of 3 with only one exception, *i.e*., the absence of the methoxylation in C-2′. This exception was assigned *via* the downfield shift of H-2′ by 0.46 ppm at *δ*_H_ 4.18 d (*J* = 3.8, 5.7 Hz), upfield shift of C-2′ by 9.7 ppm at *δ*_C_ 70.4, and the absence of the proton and carbon resonances of the methyl of methoxyl group. The ^1^H ^1^H COSY correlations (Fig. 3) of H-4′ (*δ*_H_ 5.72 dd (*J* = 6.6, 15.8 Hz))/H-5′ (*δ*_H_ 5.06 dd (*J* = 4.0, 6.5 Hz)), H-5′/H-6′ (*δ*_H_ 3.78 dd (*J* = 4.0, 6.5 Hz)), H-6′/H-7′ (*δ*_H_ 1.06 d (*J* = 6.5 Hz)) along with the ^3^*J* HMBC (Fig. 3) correlations of H-5′/Ac-CO (*δ*_C_ 170.7) and H-7′/C-5′ (*δ*_C_ 77.7) confirmed the hydroxylation of C-1′, C-2′, and C-6′ along with acetoxylation of C-5′. From these data, 5 was established as 6-[5′-acetyloxy-1′,2′,6′-trihydroxy-3-heptenyl]-5,6-dihydro-2*H*-pyran-2-one. As well, the *trans* (*E*) configuration of the C-3′/C-4′ olefinic system was constructed *via* the large coupling constants of both sets at 15.9 Hz.^12,13^ The construction of the absolute stereochemistry of 5 was performed by the positive ECD cotton effect at (Δ*ε*) 257.4 nm (+31.8).^12^ So, 5 was predicted as (6*R*,5′*R*,6′*S*,1′*R*,2′*R*)-6-[5′-acetyloxy-1′,2′,6′-trihydroxy-3-heptenyl]-5,6-dihydro-2*H*-pyran-2-one (ternifolipyron D).

The positive mode HRCIMS molecular ion peak at *m*/*z* at 343.1755 of 6 revealed a molecular formula of C_17_H_27_O_7_ (calc. 343.1757) alongside of five unsaturation indexes. The ^1^H (Table 1) and ^13^C (Table 2) NMR data of 6, that exhibited seventeen carbon signals, affirmed that it was very close to 3 with only the exception of replacement of acetyl by butanoyl group in C-5′. The butanoyl group was determined *via* its characteristic ^1^H/^13^C signals at *δ*_H_ 2.26 t (*J* = 7.4 Hz)/*δ*_C_ 35.7, *δ*_H_ 1.55 m/*δ*_C_ 18.1, and *δ*_H_ 0.86 t (*J* = 7.4 Hz)/*δ*_C_ 12.5 in addition to its carbonyl at *δ*_C_ 173.1. The sequence of the butanoyl group was established by the ^1^H ^1^H COSY of the butanoyl protons, CH_3_ (*δ*_H_ 0.86 t (*J* = 7.4 Hz)/CH_2_ (*δ*_H_ 1.55 m), and CH_2_ (*δ*_H_ 1.55 m)/CH_2_ (*δ*_H_ 2.26 t (*J* = 7.4 Hz)), along with the ^3^*J* HMBC (Fig. 3) correlations of CH_2_ (*δ*_H_ 1.55 m)/CO (*δ*_C_ 173.1), and CH_3_ (*δ*_H_ 0.86 (t (*J* = 7.4 Hz))/C_H2_ (*δ*_C_ 35.7). While the placement of butanoyl group in C-5′ was proven through ^3^*J* HMBC (Fig. 3) correlation of H-5′ (*δ*_H_ 5.07 ddd (*J* = 0.7, 4.2, 10.4 Hz))/CO (*δ*_C_ 173.1). Based on these data, the structure of 6 was established as 6-[5′-butyroyl-1′,6′-dihydroxy-2′-methoxy-3-heptenyl]-5,6-dihydro-2*H*-pyran-2-one. The large coupling constants of the olefinic bond, C-3′/C-4′, at 15.8 Hz confirmed its *trans* (*E*) configuration.^12,13^ Furthermore, the absolute configuration verification of 6 was achieved by the positive ECD cotton effect at (Δ*ε*) 258.6 nm (+41.8).^13^ So, 6 was predicted as (6*R*,5′*R*,6′*S*,1′*R*,2′*R*)-6-[5′-butyroyl-1′,6′-dihydroxy-2′-methoxy-3-heptenyl]-5,6-dihydro-2*H*-pyran-2-one (ternifolipyron E).

The molecular formula of 7 was predicted as C_17_H_28_O_8_ (calc. 371.1706) from the HRCIMS molecular ion peak *m*/*z* at 371.1705, showing four unsaturation indexes. The structure of 7 was closely constructed as that of 5*via* the ^1^H (Table 1) and ^13^C (Table 2) NMR data, except the presence of one acetoxyl and one butanoyl groups instead of one acetoxyl group in 5. As described in 6, the characterization of the butanoyl group was performed by the assigned ^1^H/^13^C signals as well as ^1^H ^1^H COSY and HMBC correlations (Fig. 3). The presence of the butanoyl group in C-6′ was confirmed *via* the downfield shift of H-6′/C-6′ by 1.23/2.1 ppm at *δ*_H_ 4.99 dddd (*J* = 3.4, 6.6, 13.2 Hz)/*δ*_C_ 70.4, along with the ^3^*J* HMBC correlation (Fig. 3) of H-6′/CO (*δ*_C_ 173.2). Subsequently, the structure of 7 was assigned as 6-[5′-acetyloxy-6′-butyroyl-1′,2′-dihydroxy-3-heptenyl]-5,6-dihydro-2*H*-pyran-2-one. As described for all above compounds, the orientation of the olefinic system, C-3′/C-4′, was concluded as *trans* (*E*) from the large coupling constants at 15.8 Hz.^12,13^ Also, the absolute stereochemistry of 7 was derived by means of ECD that exhibited a positive cotton effect at (Δ*ε*) 259.2 nm (+94.7).^12^ So, 7 was predicted as (6*R*,5′*R*,6′*S*,1′*R*,2′*R*)-6-[5′-acetyloxy-6′-butyroyl-1′,2′-dihydroxy-3-heptenyl]-5,6-dihydro-2*H*-pyran-2-one (ternifolipyron F).

The HRTOFESI-MS of 8 exhibited a molecular ion peak at *m*/*z* 407.1678, affirmed the molecular formula of C_19_H_28_O_8_Na (calc. 407.1682) and five unsaturation indexes. According to the ^1^H (Table 1) and ^13^C (Table 2) NMR data, the structure of 8 was closely identical to that of 1, except for the existence of one acetoxyl and one butanoyl group instead of the two acetoxyl groups in 1. As mentioned for the compounds above, the butanoyl group was validated by the assigned ^1^H/^13^C signals, ^1^H ^1^H COSY, and HMBC correlations (Fig. 3). Moreover, the butanoyl group's location in C-6′ was confirmed by the ^3^*J* HMBC correlation of H-6′ (*δ*_H_ 5.41 ddd (*J* = 3.5, 5.0 Hz))/CO (*δ*_C_ 173.1) (Fig. 3). After considering the aforementioned data, the structure of 8 was determined to be 6-[5′-acetyloxy-6′-butyroyl-1′-hydroxy-2′-methoxy-3-heptenyl]-5,6-dihydro-2*H*-pyran-2-one. The C-3′/C-4′ olefinic system's orientation was determined to be *trans* (*E*) given a large coupling constant at 15.8 Hz as indicated in all of the compounds above.^12,13^ The absolute stereochemistry of compound 8 was verified using the ECD, which showed a positive cotton effect at (Δ*ε*) 257.8 nm (+104.8), in comparison to data from compound 1 and published data.^12^ Thereby, the predicted structure of 8 was (6*R*,5′*R*,6′*S*,1′*R*,2′*R*)-6-[5′-acetyloxy-6′-butyroyl-1′-hydroxy-2′-methoxy-3-heptenyl]-5,6-dihydro-2*H*-pyran-2-one (ternifolipyron G).

Based on the molecular ion peak at *m*/*z* 267.1236 in the HRCIMS of 9, the chemical formula was determined to be C_14_H_19_O_5_ (calc. 267.1232), which revealed five unsaturation indices. Based upon the analysis of the ^1^H and ^13^C (Table 3) NMR data, which exhibited 14 carbon resonances, the structure of 9 was closely linked to that of 2, with a few notable minor exceptions. These exceptions were summarized in (i) the presence of two olefinic systems at *δ*_H_ 5.49 dd (*J* = 8.5, 11.0 Hz)/*δ*_C_ 127.1, *δ*_H_ 6.16 t (*J* = 11.0 Hz)/*δ*_C_ 131.9, *δ*_H_ 6.56 ddt (*J* = 1.0, 11.0, 15.2 Hz)/*δ*_C_ 125.9, and *δ*_H_ 5.76 dd (*J* = 6.5, 15.2 Hz)/*δ*_C_ 135.5, and (ii) the presence of the only two functional groups in the heptanyl chain, including one hydroxyl at *δ*_H_ 4.10 ddd (*J* = 1.0, 4.4, 6.5 Hz)/*δ*_C_ 73.3 and one acetoxyl at *δ*_H_ 4.78 m/*δ*_C_ 73.0. In addition to the C-3′/C-4′ olefinic system in all above compounds, the other olefinic system was located in C-1′/C-2′ based upon the ^1^H ^1^H COSY correlations (Fig. 3) of H-6 (*δ*_H_ 5.40 m)/H-1′ (*δ*_H_ 5.49 dd (8.5, 11.0)), H-1′/H-2′ (*δ*_H_ 6.16 t (*J* = 11.0 Hz)), H-2′/H-3′ (*δ*_H_ 6.56 ddt (*J* = 1.0, 11.0, 15.2 Hz)), H-3′, H-4′ (*δ*_H_ 5.76 dd (*J* = 6.5, 15.2 Hz)). The site of C-1′/C-2′ olefinic system was assured by ^3^*J* HMBC correlations (Fig. 3) of the H-5 (*δ*_H_ 2.38 m)/C-1′ (*δ*_C_ 127.1), H-6/C-2′ (*δ*_C_ 131.9), H-1′/C-3′ (*δ*_C_ 125.9), and H-2′/C-4′ (*δ*_C_ 135.5). Also, the presence of the hydroxyl and acetoxyl groups in C-5′ and C-6′, respectively, was confirmed by the same described ^1^H ^1^H COSY and HMBC correlations in compound 2. After taking all aforementioned information into account, the structure of 9 was determined to be 6-[6′-acetyloxy-5′-hydroxy-1,3-heptadienyl]-5,6-dihydro-2*H*-pyran-2-one. According to what was previously stated, the significant coupling constant at 15.7 Hz validated the geometry of the C-3′/C-4′ olefinic system as *trans* (*E*).^12,13^ In contrast, the C-1′/C-2′ olefinic system's geometry was predicted to be *cis* (*Z*) according to the modest coupling constant at 11.0 Hz in both sites.^13,14,24^ The absolute configuration of 9 was ascertained by the ECD study, which showed a positive cotton effect at (Δ*ε*) 264.0 nm (+35.3) by comparison with compound 1, other compounds, and the literature.^12^ With the aforementioned information, 9 was ultimately determined as (6*R*,5′*R*,6′*S*)-6-[6′-acetyloxy-5′-hydroxy-1*Z*,3*E*-heptadienyl]-5,6-dihydro-2*H*-pyran-2-one (ternifolipyron H).


^1^H and ^13^C NMR spectral data of 9–11^a^

**Table:** 

No.	9		10		11	
	*δ*_H_	*δ*_C_	*δ*_H_	*δ*_C_	*δ*_H_	*δ*_C_
1	—	—	—	—	—	—
2	—	165.2	—	165.0	—	164.6
3	5.92 dd (1.6, 10.0)	120.1	5.92 dd (1.7, 9.8)	120.1	5.92 dddd (1.0, 2.6, 7.2, 9.8)	120.1
4	6.96 m	146.6	6.95 m	146.5	6.97 m	146.3
5	2.38 m	29.4	2.37 m	29.3	2.33 m, 2.43 m	29.4
6	5.40 m	74.2	5.39 m	74.1	5.32 m	74.4
1′	5.49 dd (8.5, 11.0)	127.1	5.55 t (8.7, 10.9)	128.5	5.64 t (7.9, 10.6)	129.9
2′	6.16 t (11.0)	131.9	6.16 t (11.1)	131.2	5.49 dd (1.1, 10.6)	130.9
3′	6.56 ddt (1.0, 11.0, 15.2)	125.9	6.59 ddt (1.0, 11.3, 15.2)	128.6	5.45 dd (3.6, 9.5)	65.9
4′	5.76 dd (6.5, 15.2)	135.5	5.73 dd (7.3, 15.2)	130.1	1.73 m, 1.89 m	35.0
5′	4.10 ddd (1.0, 4.4, 6.5)	73.3	5.33 dddd (1.0, 3.6, 3.8, 7.3)	75.0	5.00 m	70.6
6′	4.78 m	73.0	5.00 m	70.3	4.89 dd (4.9, 6.5)	70.6
7′	1.08 d (6.5)	13.9	1.09 d (6.6)	14.0	1.08 d (6.5)	15.0

a All the compounds were measured in CD_3_OD at 500 MHz; the coupling constants (*J* in Hz) are given in parentheses.

**Table:** 

6′-Ac			5′-Ac			4′-Ac		
CO	—	171.0	CO	—	170.3	CO	—	170.6
CH_3_	1.96 s	19.7	CH_3_	1.93 s	19.5	CH_3_	1.90 s	19.4
			6′-But			5′-Ac		
			CO	—	173.2	CO	—	170.8
			CH_2_	2.18 t (7.4)	35.7	CH_3_	1.94 s	19.5
			CH_2_	1.53 m	18.1	6′-But		
			CH_3_	0.85 t (7.5)	12.5	CO	—	173.0
						CH_2_	2.20 t (7.3)	35.7
						CH_2_	1.54 m	18.1
						CH_3_	0.85 t (7.4)	12.5

Compound 10's TOFESIMS results showed a molecular ion peak at *m*/*z* 359.1465 that revealed the molecular formula to be C_18_H_24_O_6_Na (calc. 359.1471) and six unsaturation indices. The study of the ^1^H and ^13^C (Table 3) NMR data, which showed 18 carbon resonances, revealed that compound 10's structure was largely similar to compound 9's, with a few significant minor deviations. These changes could be clearly seen when there was only one butanoyl group and one acetoxyl group present. The ^3^*J* HMBC correlation (Fig. 3) of H-5′ (*δ*_H_ 5.33 dddd (*J* = 1.0, 3.6, 3.8, 7.3 Hz))/Ac-CO (*δ*_C_ 170.3) corroborated the placement of the acetoxyl group in C-5′. The butanoyl group was created, as mentioned for the aforementioned compounds by the ^1^H ^1^H COSY and HMBC correlations (Fig. 3) of its protons and carbons. The ^3^*J* HMBC correlations (Fig. 3) of the H-6′ (*δ*_H_ 5.00 m)/But-CO (*δ*_C_ 173.2) showed that the butanoyl group existed in C-6′. The olefinic systems, C-1′/C-2′ and C-3′/C-4′, in 10 were confirmed by the same in 9. Based on the data discussed above, 10 was chemically created as 6-[5′-acetyloxy-6′-butyroyl-1,3-heptadienyl]-5,6-dihydro-2*H*-pyran-2-one. The *cis* (*Z*) and *trans* (*E*) geometries of the olefinic systems were confirmed by the coupling constants of C-1′/C-2′ and C-3′/C-4′, respectively, at 10.9 and 15.2 Hz.^12–14,24^ In a similar way, the absolute configuration of 10 was established using the ECD experimental data, which revealed a positive cotton effect at (Δ*ε*) 264.0 nm (+76.9).^14^ Based on the information above, 10 was eventually (6*R*,5′*R*,6′*S*)-6-[5′-acetyloxy-6′-butyroyl-1*Z*,3*E*-heptadienyl]-5,6-dihydro-2*H*-pyran-2-one (ternifolipyron I).

From the HRCIMS molecular ion peak at *m*/*z* 397.1871, compound 11's molecular formula was inferred to be C_20_H_29_O_8_ (calc. 397.1862) coupled with six unsaturation indices. The 11's ^1^H and ^13^C (Table 3) NMR data, which displayed 20 carbon signals, demonstrated that it was largely similar to 10, with prominent variations. These two variations were located in the heptanyl chain as follows: (i) presence of only one olefinic system, and (ii) the existence of a new acetoxylated carbon at *δ*_H_ 5.45 dd (*J* = 3.6, 9.5 Hz)/*δ*_C_ 65.9 along with one methylene carbon at *δ*_H_ 1.73 m, 1.89 m/*δ*_C_ 35.0. The olefinic system was demonstrated to be C-1′/C-2′ by the ^1^H ^1^H COSY correlations (Fig. 3) of the H-6 (*δ*_H_ 5.32 m)/H-1′ (*δ*_H_ 5.64 t (*J* = 7.9, 10.6 Hz)), H-1′/H-2′ (*δ*_H_ 5.49 dd (*J* = 1.1, 10.6 Hz)) along with the ^3^*J* HMBC correlation (Fig. 3) of H-1′/C-5 (*δ*_C_ 29.4). Furthermore, the new acetoxylated carbon and methylene carbon were localized in C-3′ and C-4′, respectively, depending on the ^1^H ^1^H COSY correlations (Fig. 3) of the H-2′/H-3′ (*δ*_H_ 5.45 dd (*J* = 3.6, 9.5 Hz)), H-3′/H-4′ (*δ*_H_ 1.73 m, 1.89 m), and H-4′/H-5′ (*δ*_H_ 5.00 m) as well as the ^3^*J* HMBC correlation (Fig. 3) of H-1′/C-3 (*δ*_C_ 65.9), H-4′/C-2′ (*δ*_C_ 130.9), and H-3′/Ac-CO (*δ*_C_ 170.6). The other sections of this compound, including 5′-acetoxyl and 6′-butyroyl, were determined as described for 10. Thus, 11 was established as 6-[6′-butyroyl-3′,5′-diacetyloxy-1-heptadienyl]-5,6-dihydro-2*H*-pyran-2-one. The C-1′/C-2′ olefinic system's *cis* (*Z*) geometry was decided by the system's modest coupling constant at 10.6 Hz.^13,14,24^ Also, 11′ absolute configuration was defined depending upon the positive cotton effect at (Δ*ε*) 257.8 nm (+91.2) in the ECD experimental data.^14^ Therefore, 11 was determined as (6*R*,3′*R*,5′*R*,6′*S*)-6-[6′-butyroyl-3′,5′-diacetyloxy-1*Z*-heptenyl]-5,6-dihydro-2*H*-pyran-2-one (ternifolipyron J).

### 2.2. Cytotoxic activity of isolates

**Fig. 4:**
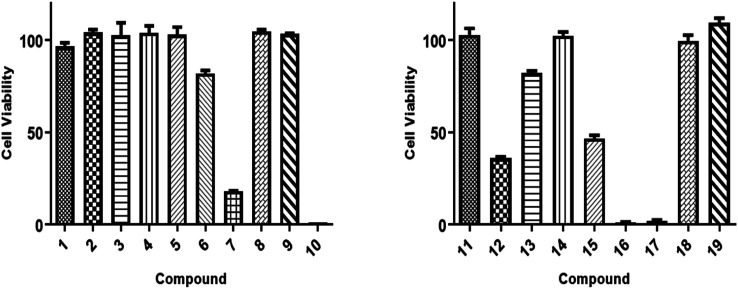
Cytotoxic activity screening results of isolates (1–19) against CCRF-CEM cancer cells at 30 μg mL^−1^ as a fixed concentration. Three different experiments' average and error bars are displayed in bars. The paired Student's *t*-test was used for statistical analysis.

**Fig. 5:**
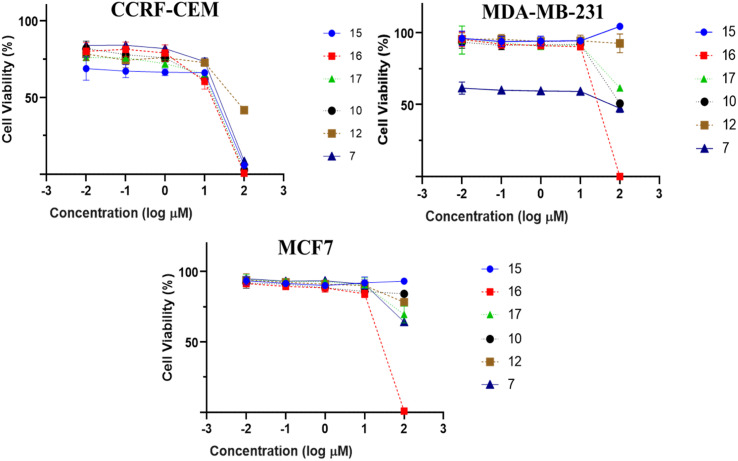
Dose response curves of isolates (1–19) against CCRF-CEM leukemia, MDA-MB-231 triple-negative breast cancer and MCF7 a breast cancer cells by resazurin assessment. Mean values and standard deviations of each three independent experiments with each six parallel measurements are shown.

All isolated compounds were initially screened against CCRF-CEM leukemia cell lines at one fixed concentration (30 μM) (Fig. 4). The compounds 7, 10, 12, and 15–17 were the most effective metabolites with this fixed concentration (30 μM). As a next step, dose–response curves were performed with concentrations in a range from 0.001 to 100 μg mL^−1^ for three cancer cell lines (CCRF-CEM, MDA-MB-23, MCF7). Ursolic acid (16) exhibited the strongest cytotoxic activity against three cancer cell lines as follows: the IC_50_ for the three cell lines were 8.37 μM, 18.04 μM, and 18.93 μM, respectively (Fig. 5).

## 3. Experimental

### 3.1. General experimental procedures

Silica gel 60 (230–400 and 100–200 mesh), pre-coated Kieselgel-60 F-254-TLC plates silica gel (Merck, Darmstadt, Germany), and Sephadex LH-20 (Pharmacia Co. Ltd) were used as packing materials for column chromatography. Isolera-one flash chromatography (Biotage; Suite C Charlotte, NC; USA) was used for flash chromatographic analysis as well as the isolation and purification processes. The compounds were purified using HPLC with a Jasco-pump (PU-980) equipped with a Jasco UV-970 intelligent detector (UV/VIS) at 210 nm. A HPLC semi-preparative Supelco C18-RP-column (250 × 10 mm, 5 μm) was also used. The optical rotation of the isolated compounds was measured by a JASCO (P-2300) polarimeter (Tokyo, Japan). A Bruker 500 Hz (MA, USA) spectrometer was used for recording the 1D NMR, including ^1^H, ^13^C, and DEPT-135, and the 2D NMR, including HSQC, HMBC, ^1^H ^1^H COSY, and NOESY. All NMR analyses were measured in deuterated methanol (CD_3_OD) at room temperature. The chemical shifts and coupling constants were given in delta (*δ*, ppm) and Hertz (Hz), respectively. CD_3_OD was referenced at *δ*_H_ 4.77 and *δ*_H_ 3.31 ppm in ^1^H NMR, and at *δ*_C_ 49.14 in ^13^C NMR. The experimentally measured electronic circular dichroism (ECD) of the compounds were measured in CH_3_OH by a JASCO-810 spectrometer. The mass spectral data including, LR and HR-MS, were derived by a JEOL (JMS-700) instrument (Tokyo, Japan).

### 3.2. Plant material

The roots of *I. ternifolius* were collected in West Imphal, Manipur, India, 24.6637° N, 93.9063° E, during March/April 2020. The identification and authentication of the collected plant were kindly performed by Dr Biseshwori Thongam, Taxonomist, Bioresource and Sustainable Development Institute, IBSD Imphal, Manipur, India. A plant voucher (No.: IBSD/Z-ITF-1578) was stored at the Plant Bioresource Division Herbarium, IBSD Imphal, India.

### 3.3. Extraction process

*I. ternifolius* roots were carefully cleaned, left for one week in a dry and shady place at room temperature until complete dryness, and then crushed using a clean plant grindery into fine powder. The extraction of the powdered roots (1.3 kg) occurred by maceration in dichloromethane-methanol (CH_2_Cl_2_–MeOH, 6 L) at room temperature for successive 72 h and filtered. The filtrate was extracted with the same steps two more times. The total extract (84.5 g) was obtained as black gum by complete drying of the overall amount of liquid extract under vacuum at 45–50 °C.

### 3.4. Metabolite isolation and purification

The extract was subjected to rapid fractionation over silica gel (230–400 mesh) column chromatography (CC) starting with *n*-hexane/CH_2_CL_2_ (1/0, 4/1, 3/2, 2/3, 1/4, 0/1) followed by CH_2_CL_2_/MeOH (4/1, 3/2, 2/3, 0/1) as elution systems afforded 46 fractions. These fractions were collected to 8 main fractions (designated YGS-1 to YGS-8) after examination with thin layer chromatography (TLC) with different solvent systems. Fraction YGS-4 (1.4 g) was fractionated over Isolera-one flash CC using a step gradient of CHCl_3_/MeOH that yielded compound 1 (178.7 mg) along with three sub-fractions (YGS-4A, YGS-4B, and YGS-4C). The further fractionation of YGS-4A (152.1 mg) with a mixture of CHCl_3_/MeOH (1 : 1) as an elution system over Sephadex LH-20 CC yielded 2 (7.9 mg), 3 (8.5 mg), 4 (10.2 mg), and 18 (17.6 mg). Fraction YGS-4B (131.2 mg) was carefully filtered, and then the clearly soluble portion was subjected to C18-RP-HPLC with an eluting system of MeOH–H_2_O (3 : 2) to yield 5 (13.4 mg), 6 (11.6 mg), and 7 (9.8 mg). With the same sequence, 9 (7.7 mg), 12 (56.8 mg), and 19 (143.2 mg) were purified from the fraction YGS-4C (94.3 mg). The filtrated methanol-soluble portion of fraction YGS-2 (1.0 g) was subjected to C18-RP-HPLC using MeOH/H_2_O (4 : 1) as eluting system and yielded 8 (14.3 mg), 15 (46.1 mg), and 16 (53.5 mg). The further fractionation of fraction YGS-3 (1.3 g) over silica gel CC using CHCl_3_/MeOH (1/0, 9/1, 4/1, 7/3, 3/2, 1/1, 2/3, and 0/1) yielded 17 (161.4 mg), 14 (128.2 mg), and sub-fraction YGS-3A. The eluting of the sub-fraction YGS-3A (68.7 mg) by MeOH–H_2_O (3 : 2) as the mobile phase using the C18-RP-HPLC led to the purification of 10 (12.4 mg), 11 (16.1 mg), and 13 (33.7 mg).

### 3.5. Preparation of bromobenzoyl derivative of 1

**Scheme 1:**
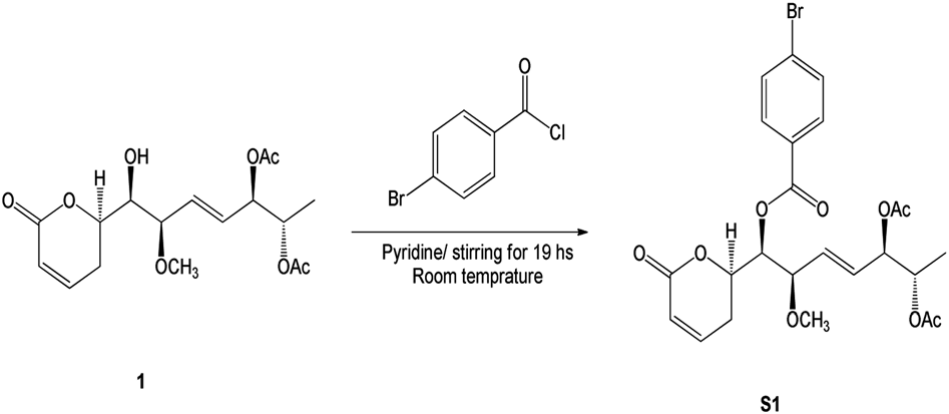
Synthetic of (6*R*,5′*R*,6′*S*,1′*R*,2′*R*)-6-[5′,6′-diacetyloxy-1′-((4-bromobenzoyl)oxy)-2′-methoxy-3*E*-heptenyl]-5,6-dihydro-2*H*-pyran-2-one (S1).

The *p*-bromobenzoyl chloride (13.3 mg, 0.06 mmol) was added to a solution of compound 1 (1.5 mg, 4.3 μmol) in dried pyridine (0.1 mL). After stirring for 19 h, at room temperature, the resulting solution was diluted with ethyl acetate and washed with aqueous KHSO_4_ (1 M) and aqueous saturated NaHCO_3_ (Scheme 1). The resulting organic layer was dried over anhydrous Na_2_SO_4_, filtrated, and concentrated under vacuum. The crude residue was purified by silica gel chromatography to give benzoate S1 (2.3 mg, 4.3 μmol) in quantitative yield as a colourless oil.

### 3.6. X-ray single crystallographic procedure of S1

Single crystals of bromobenzoate derivative of 1 (S1) were obtained by slow evaporation of a hexane and ethyl acetate solution, selected and fitted onto a glass fiber, and measured at −173 °C with a Bruker Apex II ultra diffractometer using MoKα radiation. Data correction and reduction were performed with the crystallographic package Apex II. The structure was solved and refined using the Bruker SHELXTL software package. All non-hydrogen atoms were refined anisotropically, and hydrogen atoms were positioned geometrically. The final anisotropic full-matrix least-squares refinement on *F*^2^ with 311 variables converged at *R*_1_ = 2.51%, for the observed data and w*R*_2_ = 5.92% for all data. The ORTEP plot was obtained by the program PLATON (A. L. Spek, 2009). Crystallographic data (excluding structure factors) for the structures of S1 have been deposited with the Cambridge Crystallographic Data Centre as supplementary publication numbers CCDC 2246696.†

### 3.7. Spectroscopic data of isolates (1–11)

#### (6*R*,5′*R*,6′*S*,1′*R*,2′*R*)-6-[5′,6′-Diacetyloxy-1′-hydroxy-2′-methoxy-3*E*-heptenyl]-5,6-dihydro-2*H*-pyran-2-one (1)

Pale yellow oil; ([*α*]_25_D +16.1; *c* 0.1, MeOH); ECD (MeOH; *c* mg mL^−1^) (Δ*ε*) + 45.1 (265.0 nm); the spectroscopic spectra including LREIMS & TOFESIMS, ^1^H (500 Hz), ^13^C NMR (125 Hz; CD_3_OD) (Fig. S1–S4†), and ECD (Fig. S94†) were inserted in the ESI data file.†

#### (6*R*,5′*R*,6′*S*,1′*R*,2′*R*)-6-[5′,6′-Diacetyloxy-1′-((4-bromobenzoyl)oxy)-2′-methoxy-3*E*-heptenyl]-5,6-dihydro-2*H*-pyran-2-one (S1)

Colorless oil, ^1^H (300 Hz): ^1^H NMR (300 MHz in CDCl_3_) *δ*_H_ 7.91 (m, 2H), 7.61 (m, 2H), 6.88 (ddd, *J* = 9.7, 5.3, 2.9 Hz, 1H), 6.06 (br d, *J* = 9.7 Hz, 1H), 5.78 (dd, *J* = 15.6, 7.1 Hz, 1H), 5.61 (dd, *J* = 15.6, 6.8 Hz, 1H), 5.38 (dd, *J* = 8.2, 2.6 Hz, 1H), 5.23 (dd, *J* = 7.1, 3.5 Hz, 1H), 4.99 (qd, *J* = 6.5, 3.5 Hz, 1H), 4.90 (ddd, *J* = 10.3, 8.2, 5.3 Hz, 1H), 4.25 (dd, *J* = 6.8, 2.6 Hz, 1H), 3.37 (s, 3H), 2.47–2.40 (m, 2H), 2.01 (s, 3H), 1.87 (s, 3H), 1.07 (d, *J* = 6.5 Hz, 3H). Crystal data: C_24_H_27_BrO_9_, MW = 539.36, monoclinic, space group *P*2_1_, *Z* = 2, *a* = 13.5903 (13) Å, *b* = 6.6575 (6) Å, *c* = 13.8833 (13) Å, *β* = 100.1930 (10)°, *V* = 1236.3 (2) Å^3^, Flack parameter = 0.020 (4), GOF = 0.937. The spectroscopic spectra including ^1^H NMR (300 Hz, Fig. S5†) was inserted in the ESI data file.†

#### Ternifolipyron A (2)

Golden yellow oil; ([*α*]^D^_25_ +29.2; *c* 0.1, MeOH); ECD (MeOH; *c* mg mL^−1^) (Δ*ε*) + 109.9 (259.2 nm); ^1^H NMR (500 Hz; CD_3_OD) and ^13^C NMR (125 Hz; CD_3_OD): presented in Tables 1 and 2, CIMS, *m*/*z* (rel. int.): 315 [M + 1]^+^ (22%), 297 (37%), 255 (21%), 233 (54%), 205 (42%), 181 (44%), 163 (34%), 127 (63%); 111 (83%); 99 (43%); 95 (100%); 43 (26%); 41 (59%); HRCIMS *m*/*z* 315.1453 (C_15_H_23_O_7_, calcd 315.1444). Unsaturation indexes: 5. The spectral data involving the LR & HRCIMS, 1D, 2D NMR (Fig. S6–S14†) and ECD (Fig. S94†) were inserted in the ESI data file.†

#### Ternifolipyron B (3)

Pale yellow gummy oil; ([*α*]^D^_25_ +22.7; *c* 0.1, MeOH); ECD (MeOH; *c* mg mL^−1^) (Δ*ε*) + 83.4 (261.8 nm); ^1^H NMR (500 Hz; CD_3_OD) and ^13^C NMR (125 Hz; CD_3_OD): presented in Tables 1 and 2, CIMS, *m*/*z* (rel. int.): 315 [M + 1]^+^ (7%), 297 (22%), 255 (20%), 238 (21%), 223 (46%), 205 (26%), 181 (84%), 163 (41%), 155 (46%), 127 (64%); 111 (65%); 99 (43%); 95 (100%); 69 (26%); 45 (22%); 41 (43%); HRCIMS *m*/*z* 315.1453 (C_15_H_23_O_7_, calcd 315.1444). Unsaturation indexes: 5. The spectral data involving the LR & HRCIMS, 1D, 2D NMR (Fig. S15–S23†) and ECD (Fig. S94†) were inserted in the ESI data file.†

#### Ternifolipyron C (4)

Dark yellow gum; ([*α*]^D^_25_ +22.7; *c* 0.1, MeOH); ECD (MeOH; *c* mg mL^−1^) (Δ*ε*) + 46.2 (258.0 nm); ^1^H NMR (500 Hz; CD_3_OD) and ^13^C NMR (125 Hz; CD_3_OD): presented in Tables 1 and 2, CIMS, *m*/*z* (rel. int.): 301 [M + 1]^+^ (4%), 283 (31%), 223 (55%), 205 (47%), 181 (64%), 163 (44%), 141 (36%), 128 (68%), 113 (47%); 97 (92%); 95 (100%); 86 (46%); 81 (38%); 69 (48%); 43 (45%); 41 (48%); HRCIMS *m*/*z* 301.1265 (C_14_H_21_O_7_, calcd 301.1209). Unsaturation indexes: 4. The spectral data involving the LR & HRCIMS, 1D, 2D NMR (Fig. S24–S32†) and ECD (Fig. S94†) were inserted in the ESI data file.†

#### Ternifolipyron D (5)

Dark yellow oil; ([*α*]^D^_25_ +29.4; *c* 0.1, MeOH); ECD (MeOH; *c* mg mL^−1^) (Δ*ε*) + 31.8 (257.4 nm); ^1^H NMR (500 Hz; CD_3_OD) and ^13^C NMR (125 Hz; CD_3_OD): presented in Tables 1 and 2, CIMS, *m*/*z* (rel. int.): 301 [M + 1]^+^ (17%), 283 (56%), 223 (100%), 205 (45%), 198 (58%), 181 (67%), 127 (100%), 111 (71%), 97 (90%); 95 (97%); 86 (54%); 81 (51%); 69 (45%); 43 (53%); 41 (98%); HRCIMS *m*/*z* 301.1269 (C_14_H_21_O_7_, calcd 301.1287). Unsaturation indexes: 4. The spectral data involving the LR & HRCIMS, 1D, 2D NMR (Fig. S33–S41†) and ECD (Fig. S94†) were inserted in the ESI data file.†

#### Ternifolipyron E (6)

Pale brown gum; ([*α*]^D^_25_ +20.0; *c* 0.1, MeOH); ECD (MeOH; *c* mg mL^−1^) (Δ*ε*) + 41.8 (258.6 nm); ^1^H NMR (500 Hz; CD_3_OD) and ^13^C NMR (125 Hz; CD_3_OD): presented in Tables 1 and 2, CIMS, *m*/*z* (rel. int.): 343 [M + 1]^+^ (18%), 325 (45%), 297 (27%), 255 (42%), 223 (62%), 183 (63%), 181 (100%), 169 (68%), 163 (49%), 127 (89%), 111 (85%), 99 (41%); 95 (86%); 81 (30%); 71 (64%); 43 (57%); 41 (28%); HRCIMS *m*/*z* 343.1755 (C_17_H_27_O_7_, calcd 343.1757). Unsaturation indexes: 5. The spectral data involving the LR & HRCIMS, 1D, 2D NMR (Fig. S42–S50†) and ECD (Fig. S94†) were inserted in the ESI data file.†

#### Ternifolipyron F (7)

Pale brown gum; ([*α*]^D^_25_ +20.5; *c* 0.1, MeOH); ECD (MeOH; *c* mg mL^−1^) (Δ*ε*) + 94.7 (259.2 nm); ^1^H NMR (500 Hz; CD_3_OD) and ^13^C NMR (125 Hz; CD_3_OD): presented in Tables 1 and 2, CIMS, *m*/*z* (rel. int.): 371 [M + 1]^+^ (6%), 353 (17%), 311 (100%), 223 (51%), 205 (38%), 183 (43%), 155 (45%), 141 (38%), 128 (64%), 113 (46%), 97 (65%), 95 (100%); 81 (41%); 71 (95%); 43 (39%); 41 (67%); HRCIMS *m*/*z* 371.1705 (C_17_H_28_O_8_, calcd 371.1706). Unsaturation indexes: 3. The spectral data involving the LR & HRCIMS, ECD, 1D, 2D NMR (Fig. S51–S59†) and ECD (Fig. S94†) were inserted in the ESI data file.†

#### Ternifolipyron G (8)

Yellow gum; ([*α*]^D^_25_ +22.7; *c* 0.1, MeOH); ECD (Me OH; *c* mg mL^−1^) (Δ*ε*) + 104.8 (257.8 nm); ^1^H NMR (500 Hz; CD_3_OD) and ^13^C NMR (125 Hz; CD_3_OD): presented in Tables 1 and 2, HRTOFESIMS *m*/*z* 407.1678 (C_19_H_28_O_8_Na, calcd 407.1682). Unsaturation indexes: 5. The spectral data involving the TOFESIMS, ECD, 1D, 2D NMR (Fig. S60–S67†) and ECD (Fig. S94†) were inserted in the ESI data file.†

#### Ternifolipyron H (9)

Dark yellow oil; ([*α*]^D^_25_ +20.5; *c* 0.1, MeOH); ECD (MeOH; *c* mg mL^−1^) (Δ*ε*) + 35.3 (264.0 nm); ^1^H NMR (500 Hz; CD_3_OD) and ^13^C NMR (125 Hz; CD_3_OD): presented in Table 3, CIMS, *m*/*z* (rel. int.): 267 [M]^+^ (5%), 249 (65%), 189 (100%), 180 (47%), 171 (36%), 162 (74%), 147 (42%), 133 (58%), 121 (20%), 105 (24%), 95 (25%), 81 (34%); 43 (66%); 41 (62%); HRCIMS *m*/*z* 267.1236 (C_14_H_19_O_5_, calcd 267.1232). Unsaturation indexes: 5. The spectral data involving the LR & HRCIMS, 1D, 2D NMR (Fig. S68–S76†) and ECD (Fig. S94†) were inserted in the ESI data file.†

#### Ternifolipyron I (10)

Dark yellow oil; ([*α*]^D^_25_ +24.3; *c* 0.1, MeOH); ECD (MeOH; *c* mg mL^−1^) (Δ*ε*) + 76.9 (264.0 nm); ^1^H NMR (500 Hz; CD_3_OD) and ^13^C NMR (125 Hz; CD_3_OD): presented in Table 3, HRTOFESIMS *m*/*z* 359.1465 (C_18_H_24_O_6_Na, calcd 359.1471). Unsaturation indexes: 5. The spectral data involving the TOFESIMS, 1D, 2D NMR (Fig. S77–S84†) and ECD (Fig. S94†) were inserted in the ESI data file.†

#### Ternifolipyron J (11)

Golden yellow oil; ([*α*]^D^_25_ +32.1; *c* 0.1, MeOH); ECD (MeOH; *c* mg mL^−1^) (Δ*ε*) + 91.1 (257.8 nm); ^1^H NMR (500 Hz; CD_3_OD) and ^13^C NMR (125 Hz; CD_3_OD): presented in Table 3, CIMS, *m*/*z* (rel. int.): 397 [M + 1]^+^ (10%), 337 (100%), 329 (24%), 277 (64%), 249 (19%), 239 (43%), 207 (57%), 189 (66%), 179 (18%), 171 (22%), 162 (24%), 151 (22%), 71 (26%), 45 (23%), 43 (19%); 41 (69%); HRCIMS *m*/*z* 397.1871 (C_20_H_29_O_8_, calcd 397.1862). Unsaturation indexes: 6. The spectral data involving the LR & HRCIMS, 1D, 2D NMR (Fig. S85–S93†) and ECD (Fig. S94†) were inserted in the ESI data file.†

### 3.8. Tumor cell lines

In RPMI 1640 medium supplemented with 10% fetal bovine serum (FBS) and 1% penicillin (100 U mL^−1^)–streptomycin (100 μg mL^−1^), CCRF-CEM leukaemia cells were grown. The MDA-MB-231-pcDNA3 and MCF-7 human breast cancer cell lines were grown in DMEM media with 10% FBS and 1% penicillin/streptomycin supplementation. All cell lines were maintained at 37 °C in a humid environment with 5% CO_2_.

### 3.9. Resazurin cell viability assay

Living cells convert the inactive dye resazurin into the fluorescent dye resorufin through a metabolic process.^25^ Suspension cells (1 × 10^4^ cells per well) and/or adherent cells (5 × 10^3^ cells per well, incubated for overnight to allow attachment) were seeded in 96-wells plate in a volume of 100 μL. Three cancer cell lines were screened using a single concentration (30 μm), and different concentrations of test substances were added to create a total volume of 200 μL for the creation of dose–response curves. After 72 h, 20 μL 0.01% w/v resazurin (Sigma-Aldrich) was added to each well. Cells were incubated for 4 h at 37 °C. Fluorescence at the excitation wavelength of 544 nm and emission at 590 nm was measured using Infinite M2000 Pro™ plate reader (Tecan, Crailsheim, Germany). Three independently performed assays for each set of six replicates were performed. Fifty percent inhibitory concentration (IC_50_) values were calculated using the concentration–response curve fit to the non-linear regression model using GraphPad Prism® v8.0 software (GraphPad Software Inc., San Diego, CA, USA). All IC_50_ values are expressed as mean ± standard deviation (SD). Previously, this protocol had been described.^26,27^

## 4. Conclusions

Ternifolipyrons A–J, ten new α-pyrone derivatives, and nine known metabolites have been isolated from the CH_2_Cl_2_–MeOH (1 : 1) extract of *I. ternifolius* roots. In addition to the ECD, X-ray signal crystal diffraction was used to determine the isolates' absolute stereochemistry. Ternifolipyron A, among of all isolated compounds, exhibited the most significant inhibitory effect on the growth of the MDA-MB-231 triple-negative breast cancer cell line, the MCF7 breast cancer cell line, and the CCRF-CEM leukaemia cell line.

## Author contributions

Conceptualization, A. I. E., M.-E. F. H. and A. U.; formal analysis, A. I. E., T. A. M., H. I., M.-E. F. H. and A. U.; investigation, A. I. E., T. A. M., N. S., H. I., M.-E. F. H. and A. U.; writing—original draft preparation, A. I. E., T. A. M., H. I., M.-E. F. H. and Y. K.; writing—review and editing, A. I. E., T. A. M., N. S., Y. K., T. E., H. I., M.-E. F. H. and A. U.; funding acquisition, A. I. E. All authors have read and agreed to the published version of the manuscript.

## Conflicts of interest

The authors declare no conflict of interest.

## Supplementary Material

RA-013-D3RA03146B-s001

RA-013-D3RA03146B-s002
