# Supplementary material for: Ternifolipyrons A–J: new cytotoxic α-pyrones from Isodon ternifolius (D. Don) Kudô
Source: RSC Adv. 2023 Jun 29;13(29):19710–20. doi: 10.1039/d3ra03146b (PMC10309080; doi:10.1039/d3ra03146b)
Supplement: RA-013-D3RA03146B-s001 [file RA-013-D3RA03146B-s001.pdf]

Electronic Supplementary Material (ESI) for RSC Advances.
This journal is © The Royal Society of Chemistry 2023

Supporting data

Ternifolipyrons A-J: New cytotoxic α-pyrones from *Isodon ternifolius* (D. Don) Kudo

Abdelsamed I. Elshamy [a,*], Tarik A. Mohamed [c], Ningombam Swapana [b,d], Yusuke Kasai[b], Masaaki Noji [b], Thomas Efferth [e], Hiroshi Imagawa [c], Mohamed-Elamir F. Hegazy [c,e,**], Akemi Umeyama [b,*]

Data	Page
**S1**: LREIMS of **1**	4
**S2**: TOESIMS of **1**	5
**S3**: ^{1}H NMR of **1**	6
**S4**: ^{13}C NMR of **1**	7
**S5**: ^{1}H NMR of (6*R*, 5\`*R*, 6\`*S*, 1\`*R*, 2\`*R*)-6-[5\`,6\`-diacetyloxy-1\`-((4-bromobenzoyl)oxy)-2\`-methoxy-3*E*-heptenyl]-5,6-dihydro-2*H*-pyran-2-one	8
**S6**: LRCIMS of **2**	9
**S7**: HRCIMS of **2**	9
**S8**: ^{1}H NMR of **2**	10
**S9**: ^{13}C NMR of **2**	11
**S10**: DEPT-135 of **2**	12
**S11**: HSQC of **2**	13
**S12**: HMBC of **2**	14
**S13**: ^{1}H ^{1}H COSY of **2**	15
**S14**: NOESY of **2**	16
**S15**: LRCIMS of **3**	17
**S16**: HRCIMS of **3**	17
**S17**: ^{1}H NMR of **3**	18
**S18**: ^{13}C NMR of **3**	19
**S19**: DEPT-135 of **3**	20
**S20**: HSQC of **3**	21
**S21**: HMBC of **3**	22
**S22**: ^{1}H ^{1}H COSY of **3**	23
**S23**: NOESY of **3**	24
**S24**: LRCIMS of **4**	25
**S25**: HRCIMS of **4**	26
**S26**: ^{1}H NMR of **4**	26
**S27**: ^{13}C NMR of **4**	27
**S28**: DEPT-135 of **4**	28
**S29**: HSQC of **4**	29
**S30**: HMBC of **4**	30
**S31**: ^{1}H ^{1}H COSY of **4**	31
**S32**: NOESY of **4**	32
**S33**: LRCIMS of **5**	33
**S34**: HRCIMS of **5**	33
**S35**: ^{1}H NMR of **5**	34

**S36**: ^{13}C NMR of **5**	35
**S37**: DEPT-135 of **5**	36
**S38**: HSQC of **5**	37
**S39**: HMBC of **5**	38
**S40**: ^{1}H ^{1}H COSY of **5**	39
**S41**: NOESY of **5**	40
**S42**: LRCIMS of **6**	41
**S43**: HRCIMS of **6**	41
**S44**: ^{1}H NMR of **6**	42
**S45**: ^{13}C NMR of **6**	43
**S46**: DEPT-135 of **6**	44
**S47**: HSQC of **6**	45
**S48**: HMBC of **6**	46
**S49**: ^{1}H ^{1}H COSY of **6**	47
**S50**: NOESY of **6**	48
**S51**: LRCIMS of **7**	49
**S52**: HRCIMS of **7**	49
**S53**: ^{1}H NMR of **7**	50
**S54**: ^{13}C NMR of **7**	51
**S55**: DEPT-135 of **7**	52
**S56**: HSQC of **7**	53
**S57**: HMBC of **7**	54
**S58**: ^{1}H ^{1}H COSY of **7**	55
**S59**: NOESY of **7**	56
**S60**: TOFESIMS of **8**	57
**S61**: ^{1}H NMR of **8**	58
**S62**: ^{13}C NMR of **8**	59
**S63**: DEPT-135 of **8**	60
**S64**: HSQC of **8**	61
**S65**: HMBC of **8**	62
**S66**: ^{1}H ^{1}H COSY of **8**	63
**S67**: NOESY of **8**	64
**S68**: LRCIMS of **9**	65
**S69**: HRCIMS of **9**	65
**S70**: ^{1}H NMR of **9**	66
**S71**: ^{13}C NMR of **9**	67
**S72**: DEPT-135 of **9**	68
**S73**: HSQC of **9**	69
**S74**: HMBC of **9**	70
**S75**: ^{1}H ^{1}H COSY of **9**	71
**S76**: NOESY of **9**	72
**S77**: TOFESIMS of **10**	73
**S78**: ^{1}H NMR of **10**	74
**S79**: ^{13}C NMR of **10**	75
**S80**: DEPT-135 of **10**	76

**S81**: HSQC of **10**	77
**S82**: HMBC of **10**	78
**S83**: ^{1}H ^{1}H COSY of **10**	79
**S84**: NOESY of **10**	80
**S85**: LRCIMS of **11**	81
**S86**: HRCIMS of **11**	81
**S87**: ^{1}H NMR of **11**	82
**S88**: ^{13}C NMR of **11**	83
**S89**: DEPT-135 of **11**	84
**S90**: HSQC of **11**	85
**S91**: HMBC of **11**	86
**S92**: ^{1}H ^{1}H COSY of **11**	87
**S93**: NOESY of **11**	88
**S94**: Experimental ECD of isolates **1-11**	89

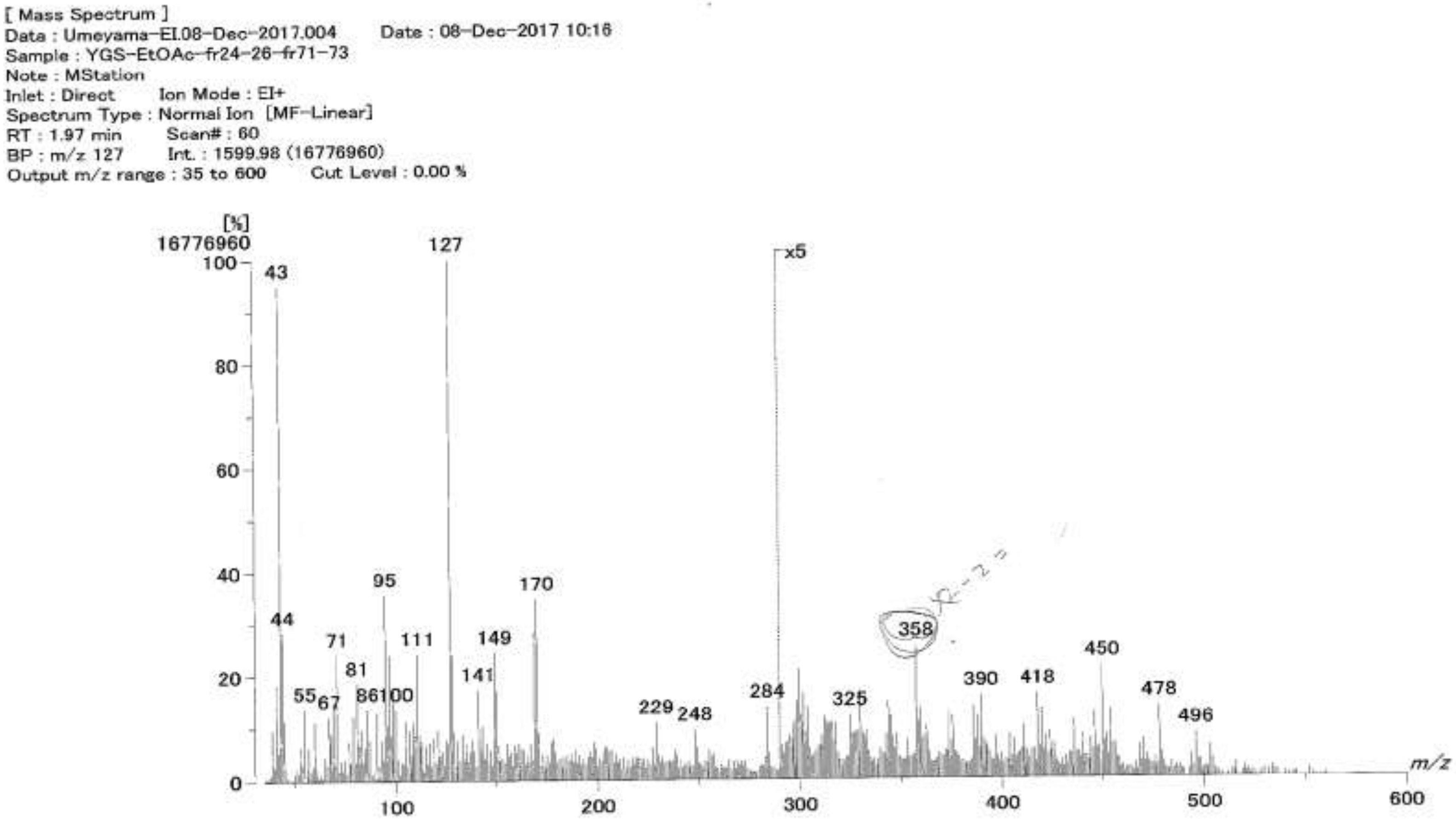

S1: LREIMS of **1**

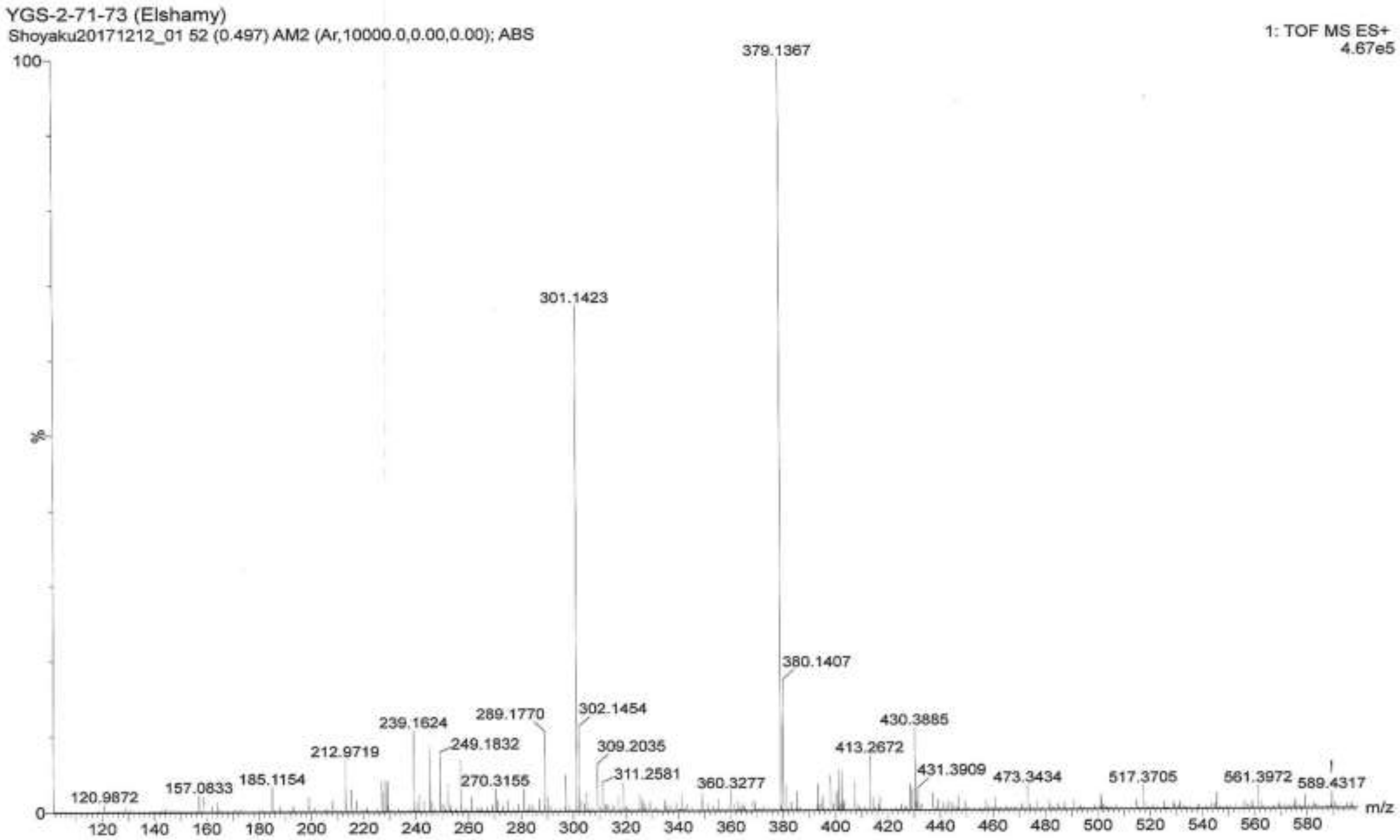

S2: TOESIMS of **1**

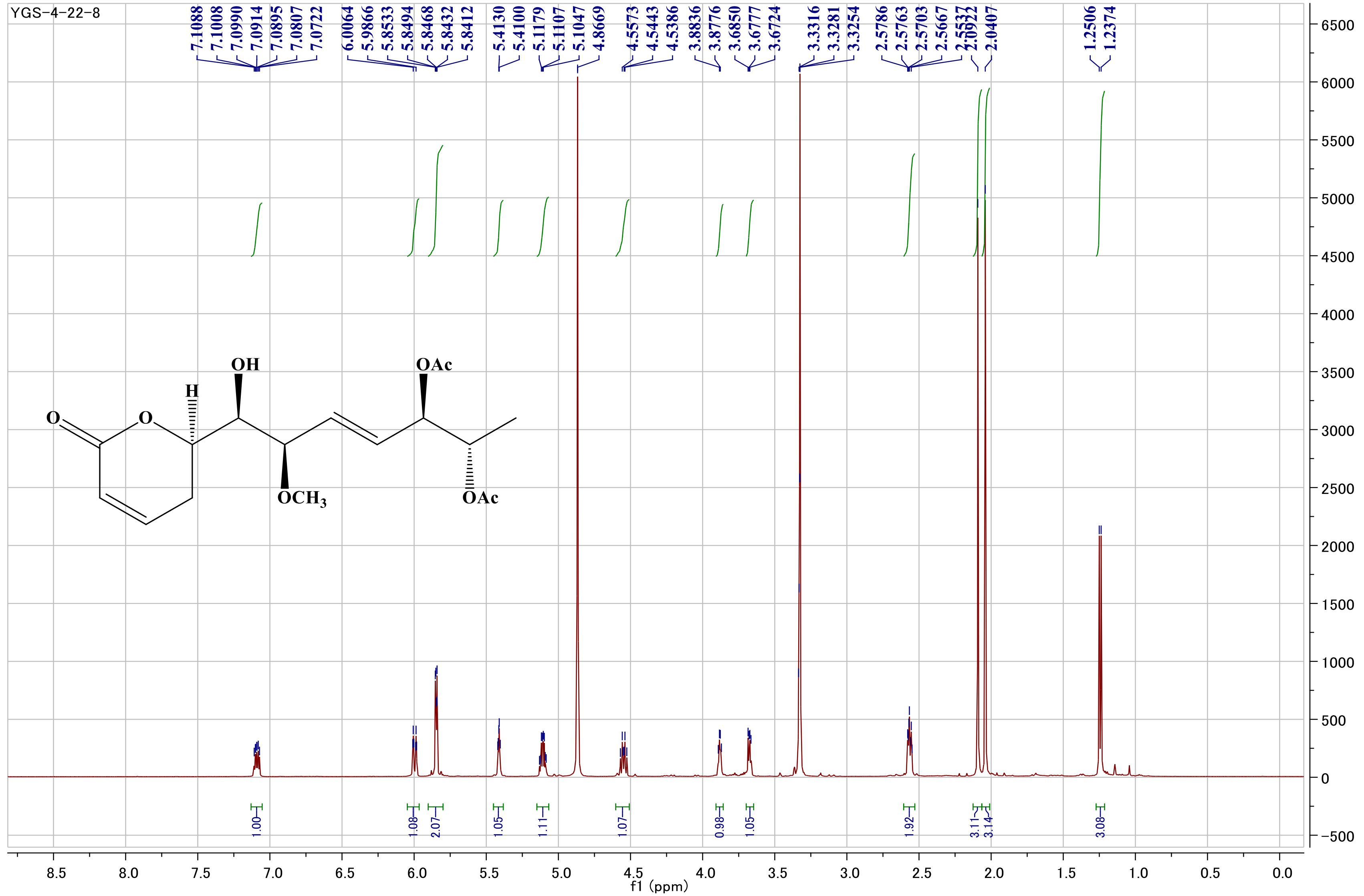

S3: ^{1}H NMR of **1**

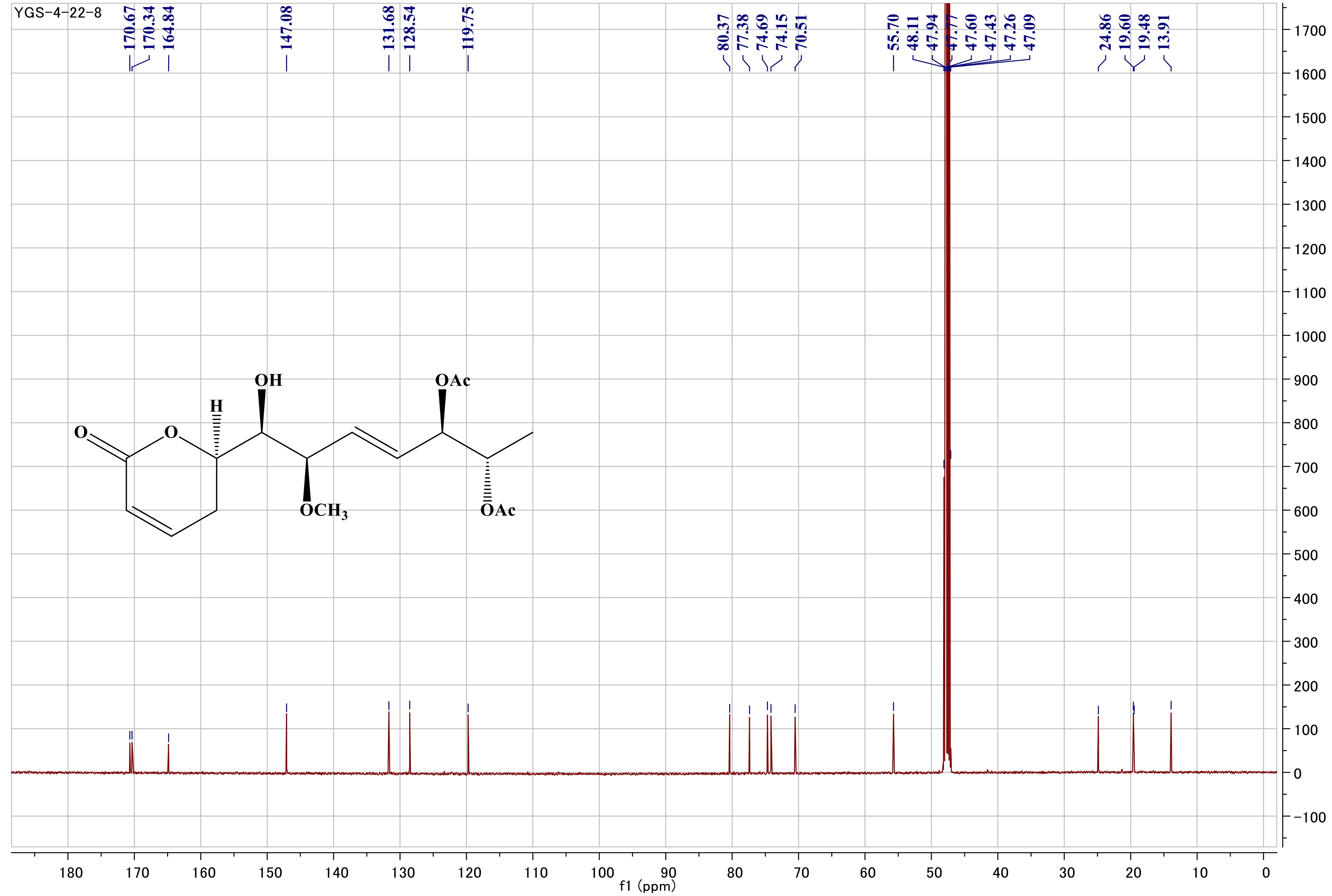

S4: ^{13}C NMR of **1**

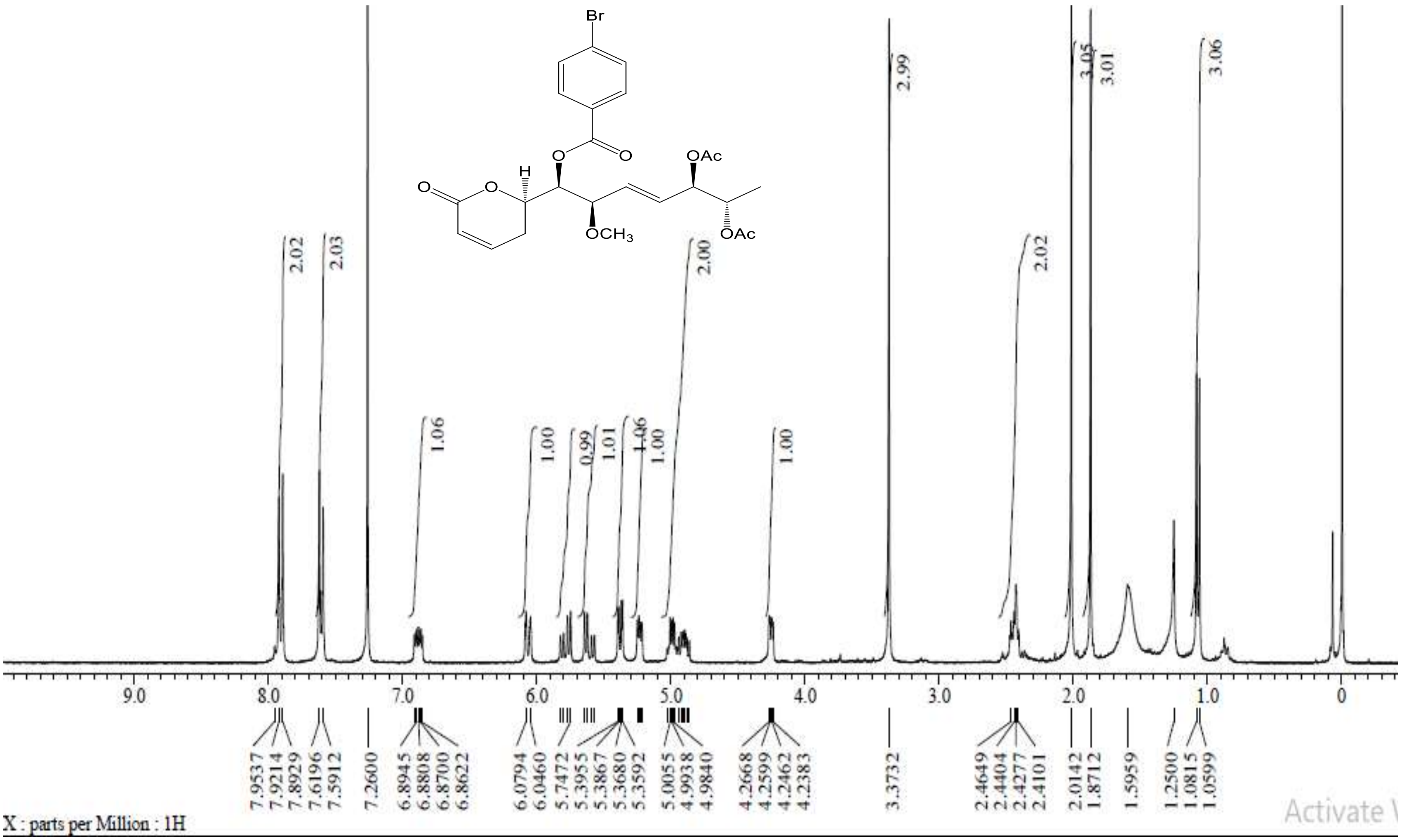

S5: ^{1}H NMR of (6*R*, 5\`*R*, 6\`*S*, 1\`*R*, 2\`*R*)-6-[5\`,6\`-diacetyloxy-1\`-((4-bromobenzoyl)oxy)-2\`-methoxy-3*E*-heptenyl]-5,6-dihydro-2*H*-pyran-2-one

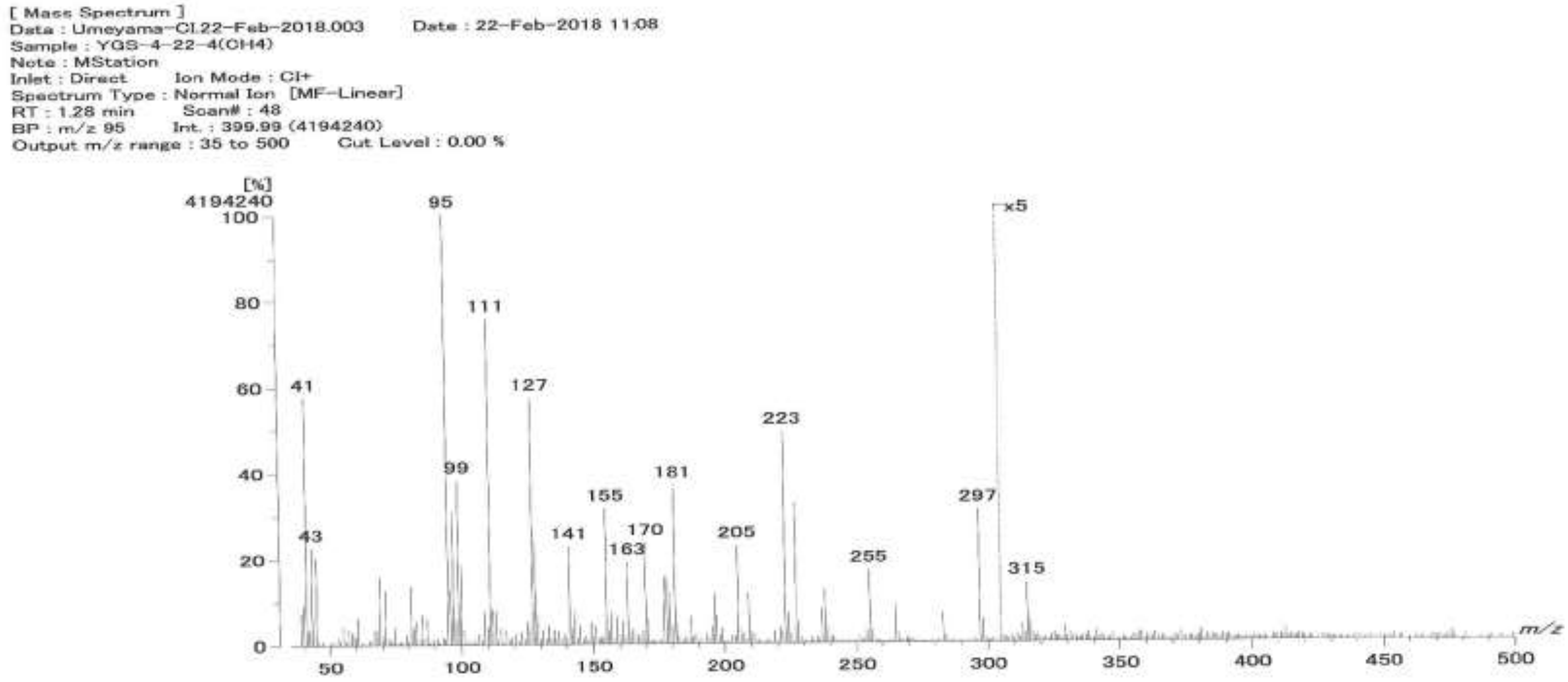

S6: LRCIMS of **2**

Data : Umeyama-CIHR.22-Feb-2018.001 Date : 22-Feb-2018 19:02
Instrument : MStation
Sample : YGS-4-22-4
Note : MStation
Inlet : Direct Ion Mode : CI+
RT : 1.17 min Scan# : 29
Elements : C 150/0, H 250/0, O 50/0
Mass Tolerance : 5mmu
Unsaturation (U.S.) : 0.0 – 15.0

	Observed m/z	Int%	Err[ppm / mmu]	U.S.	Composition
1	315.1453	10.33	+2.9 / +0.9	4.5	C15 H23 O7

S7: HRCIMS of **2**

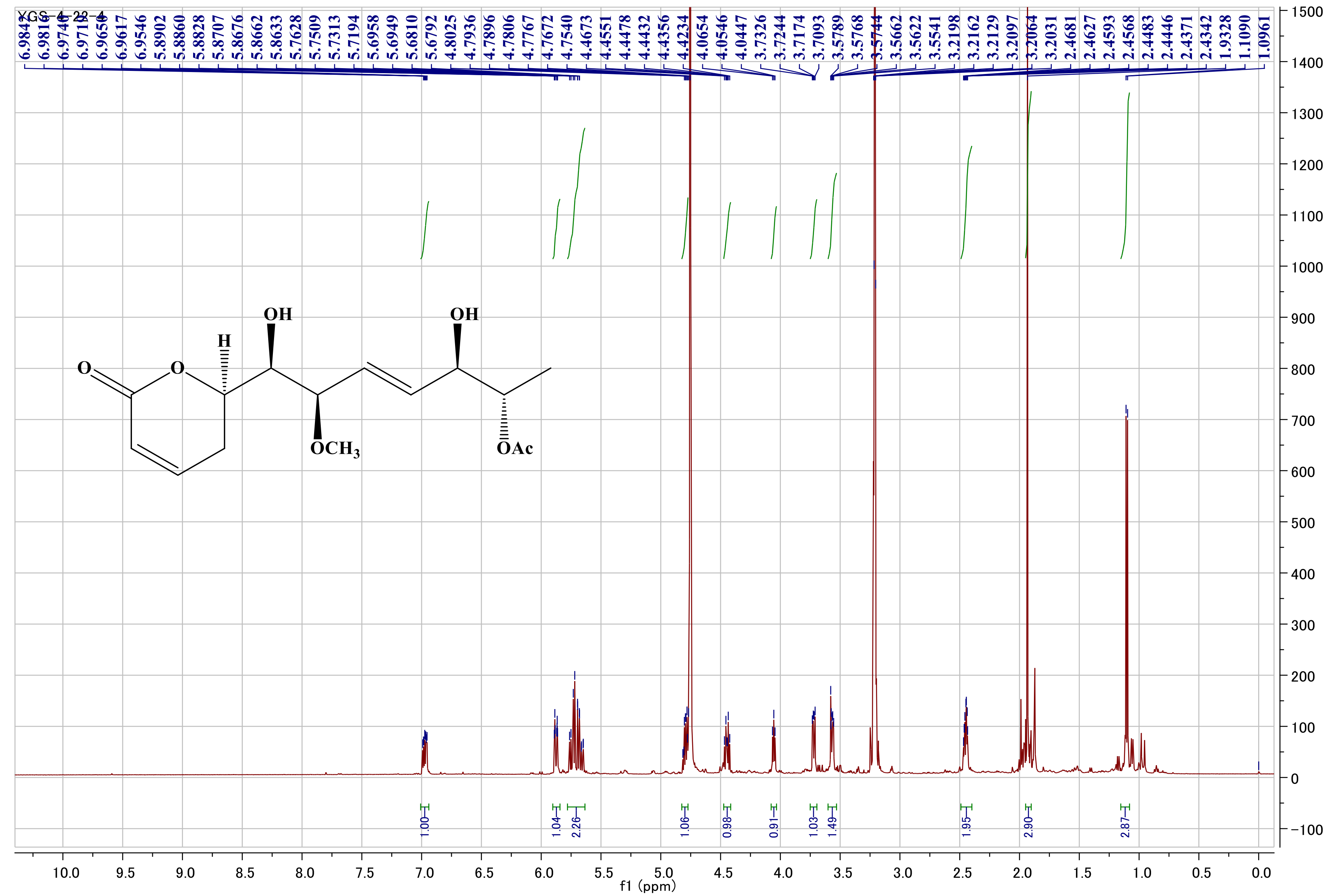

S8: ^{1}H NMR of **2**

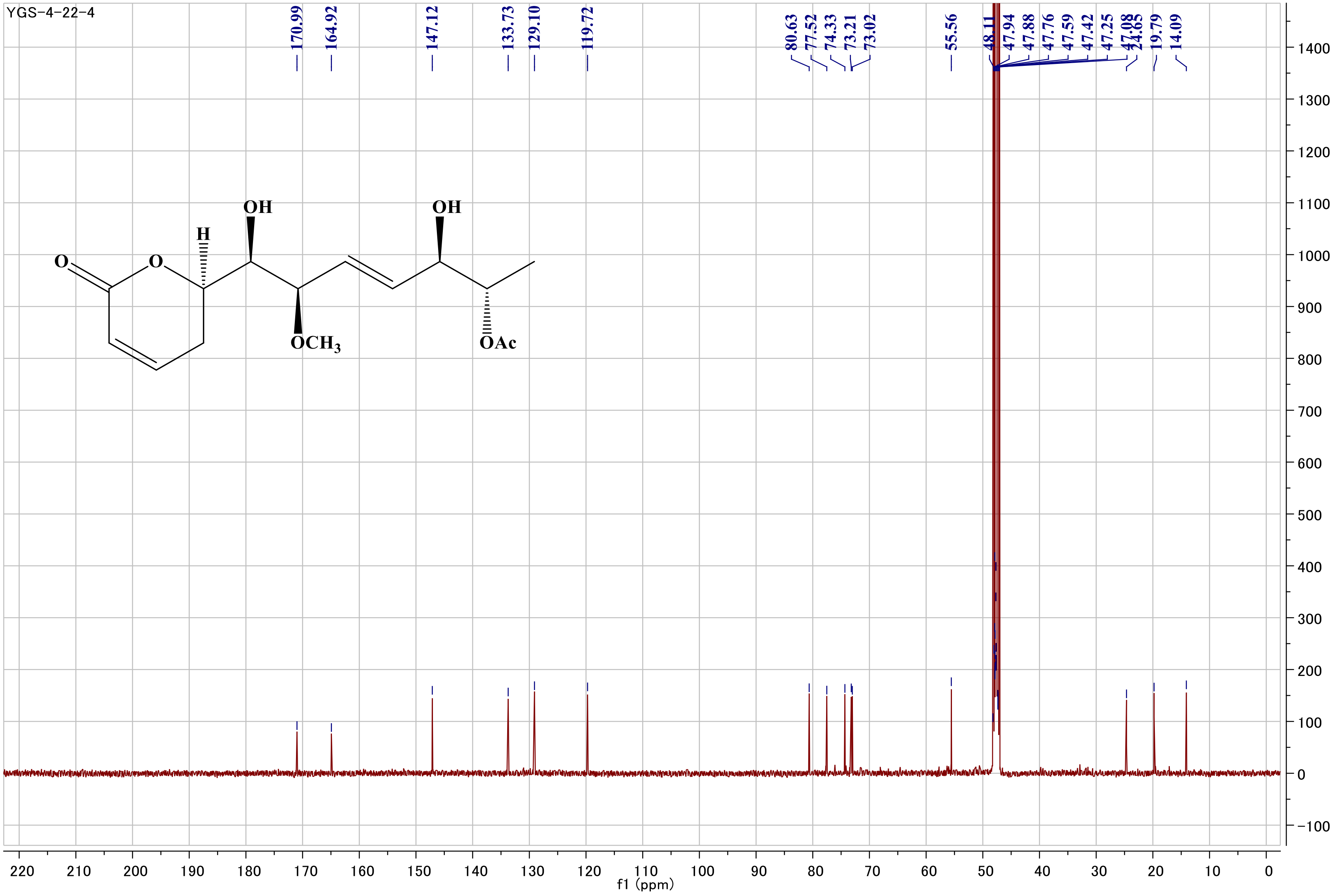

S9: ^{13}C NMR of **2**

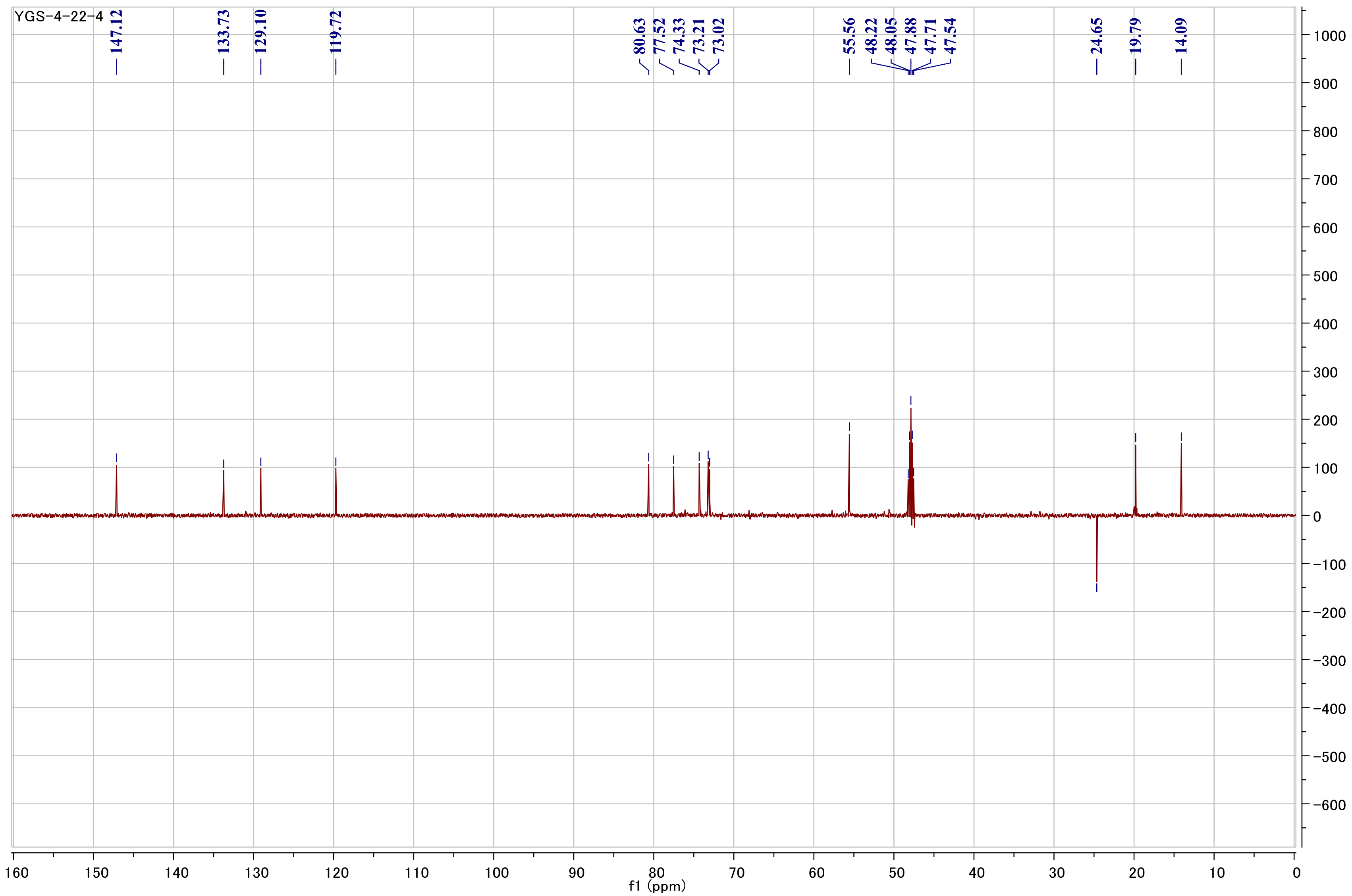

S10: DEPT-135 of **2**

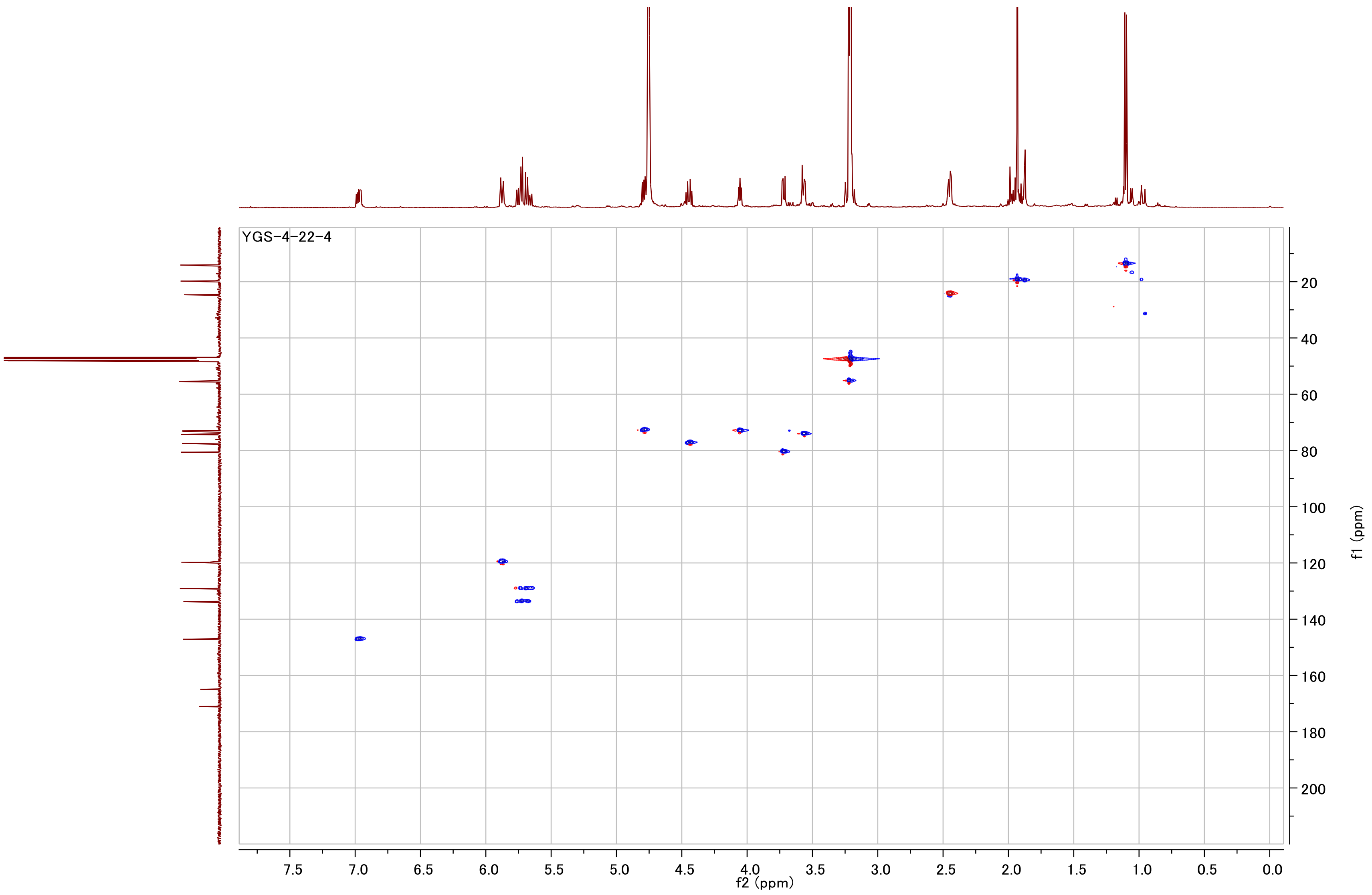

YGS-4-22-4
7.5
7.0
6.5
6.0
5.5
5.0
4.5
4.0
3.5
3.0
2.5
2.0
1.5
1.0
0.5
0.0
f2 (ppm)
20
40
60
80
100
120
140
160
180
200
f1 (ppm)

S11: HSQC of **2**

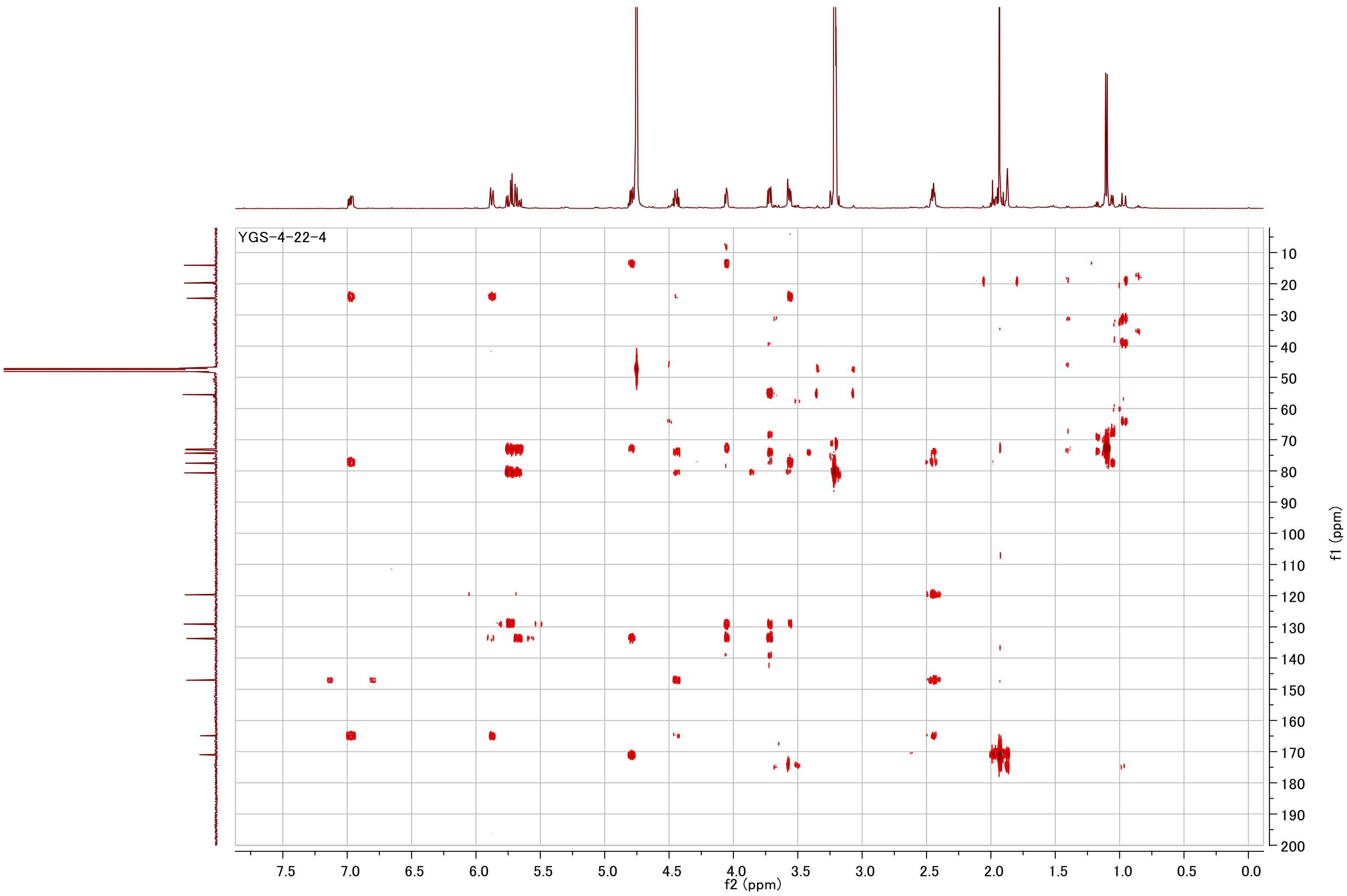
YGS-4-22-4
7.5
7.0
6.5
6.0
5.5
5.0
4.5
4.0
3.5
3.0
2.5
2.0
1.5
1.0
0.5
0.0
f2 (ppm)
10
20
30
40
50
60
70
80
90
100
110
120
130
140
150
160
170
180
190
200
f1 (ppm)

S12: HMBC of **2**

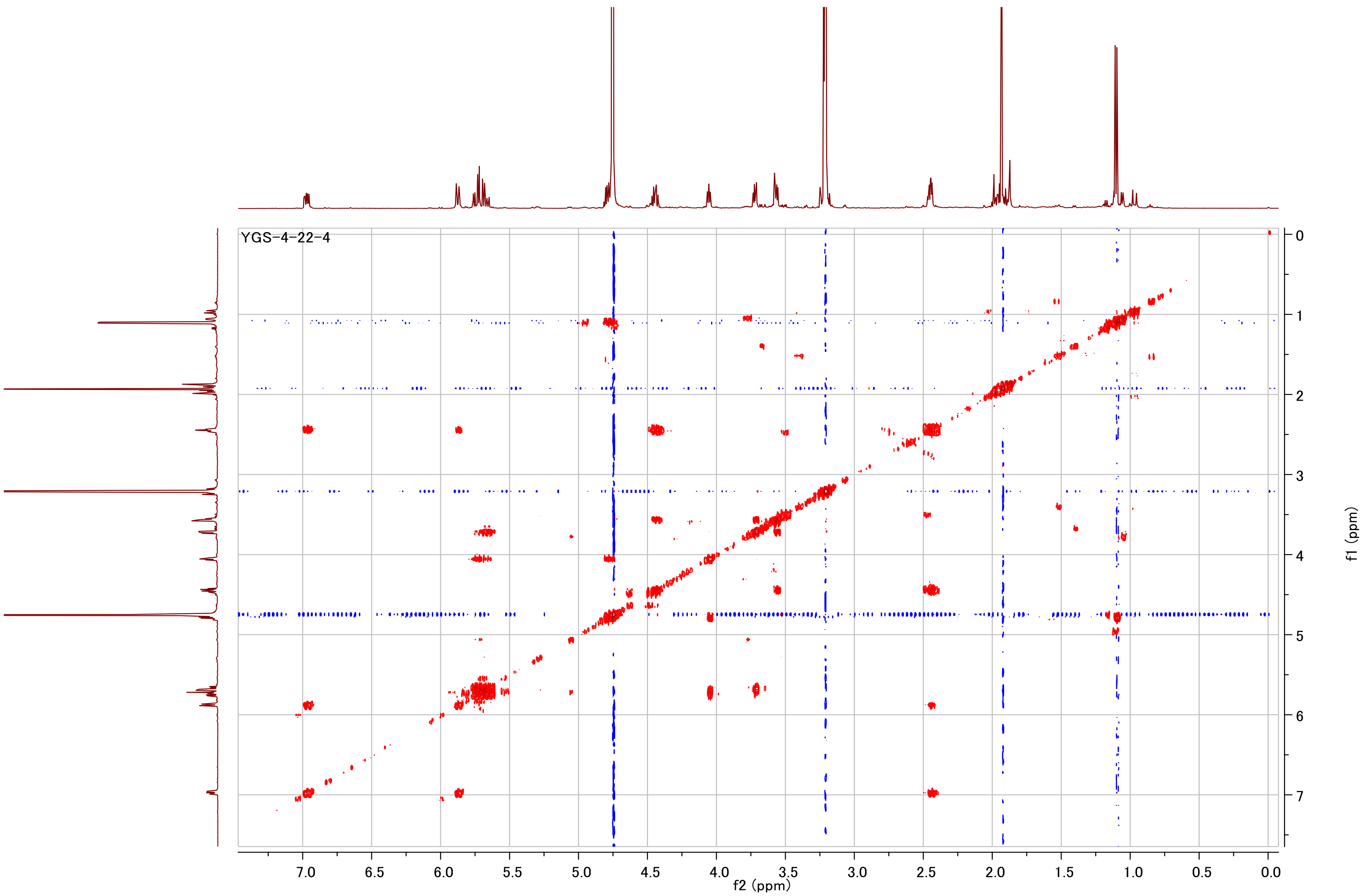

S13: ^{1}H ^{1}H COSY of **2**

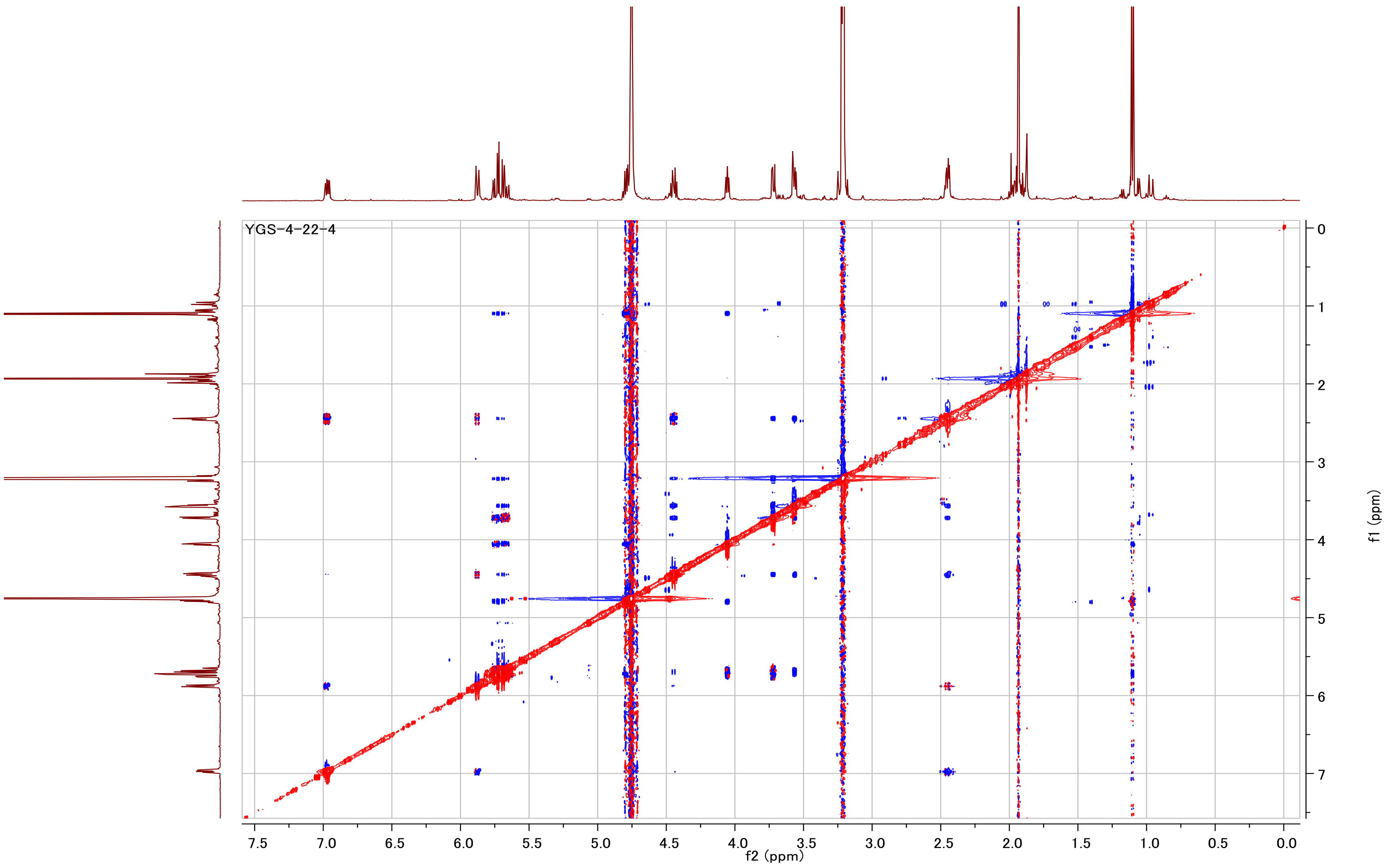

S14: NOESY of **2**

[Mass Spectrum]
Data : Umeyama-CI.22-Feb-2018.004 Date : 22-Feb-2018 18:21
Sample : YGS-4-22-3(CH4)
Note : MStation
Inlet : Direct Ion Mode : CI+
Spectrum Type : Normal Ion [MF-Linear]
RT : 1.01 min Scan# : 38
BP : m/z 95 Int. : 293.97 (3082496)
Output m/z range : 35 to 500 Cut Level : 0.00 %

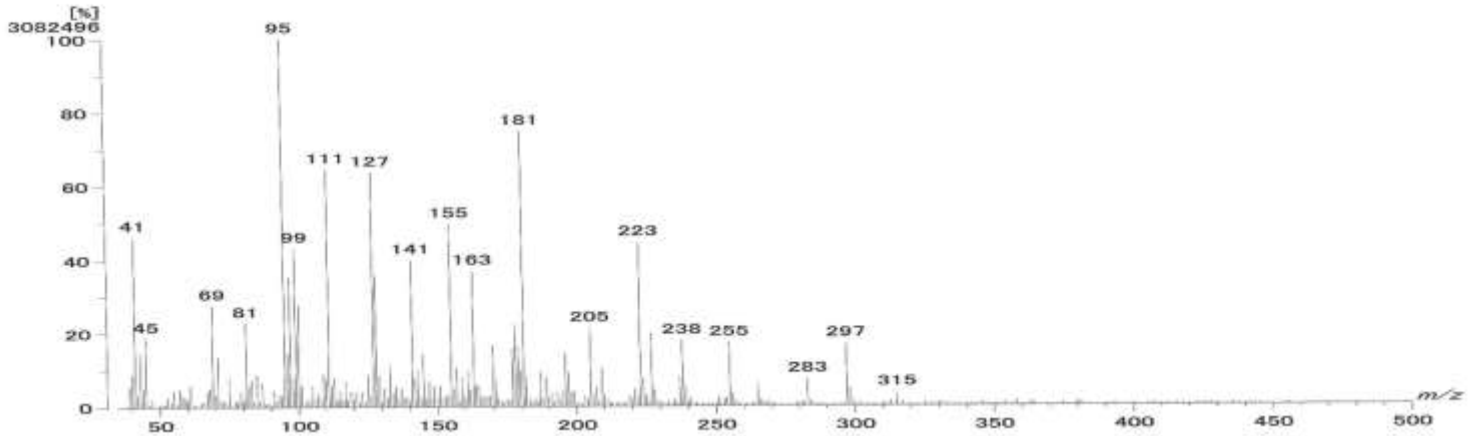

S15: LRCIMS of **3**

Data : Umeyama-CIHR.26-Feb-2018.003 Date : 26-Feb-2018 15:41
Instrument : MStation
Sample : YGS-4-22-3
Note : MStation
Inlet : Direct Ion Mode : CI+
RT : 0.96 min Scan# : 24
Elements : C 150/0, H 250/0, O 50/0
Mass Tolerance : 5mmu
Unsaturation (U.S.) : 0.0 – 15.0

	Observed m/z	Int%	Err[ppm / mmu]	U.S.	Composition
1	315.1439	5.87	−1.5 / −0.5	4.5	C15 H23 O7

S16: HRCIMS of **3**

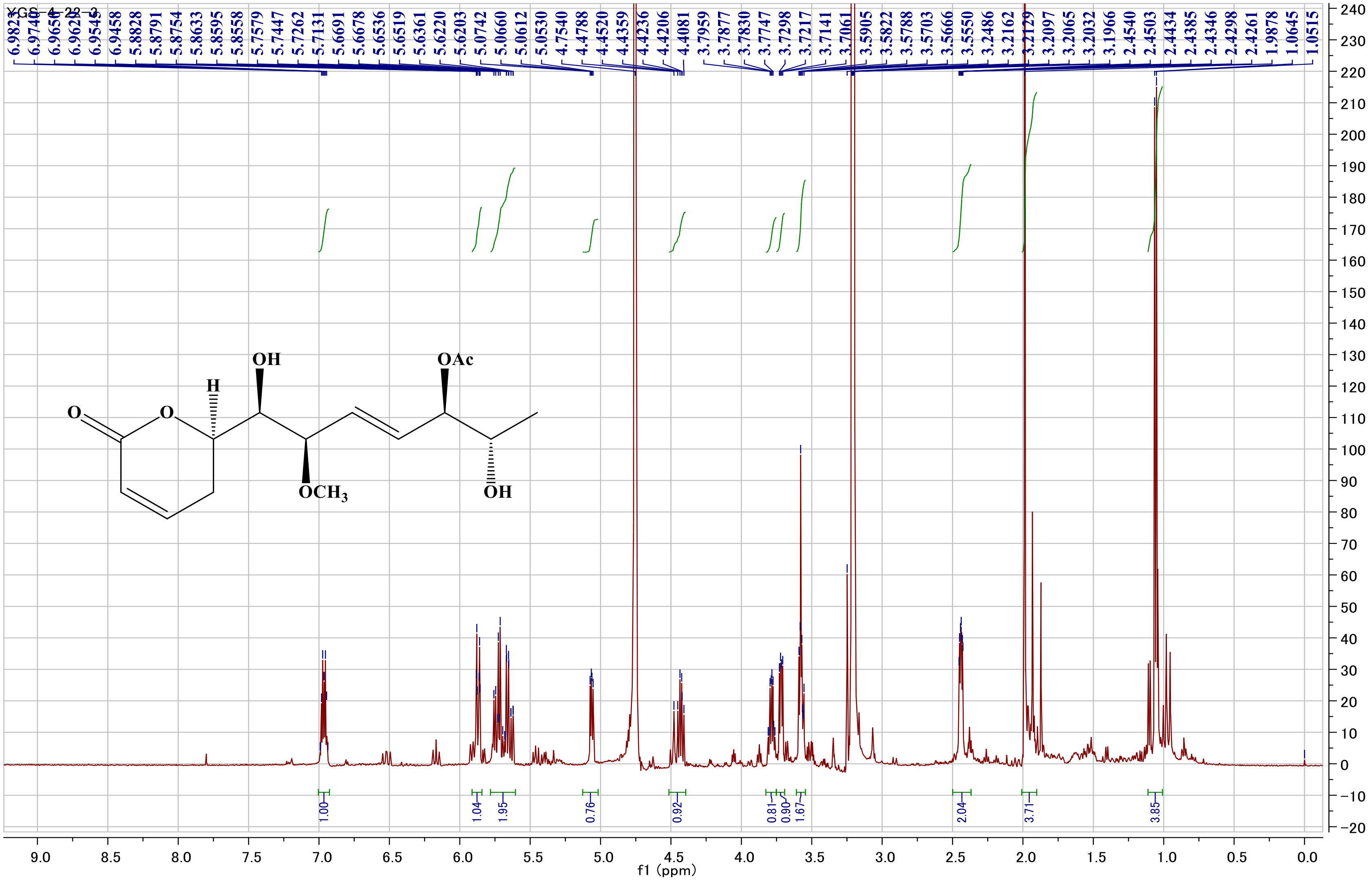

S17: ^{1}H NMR of **3**

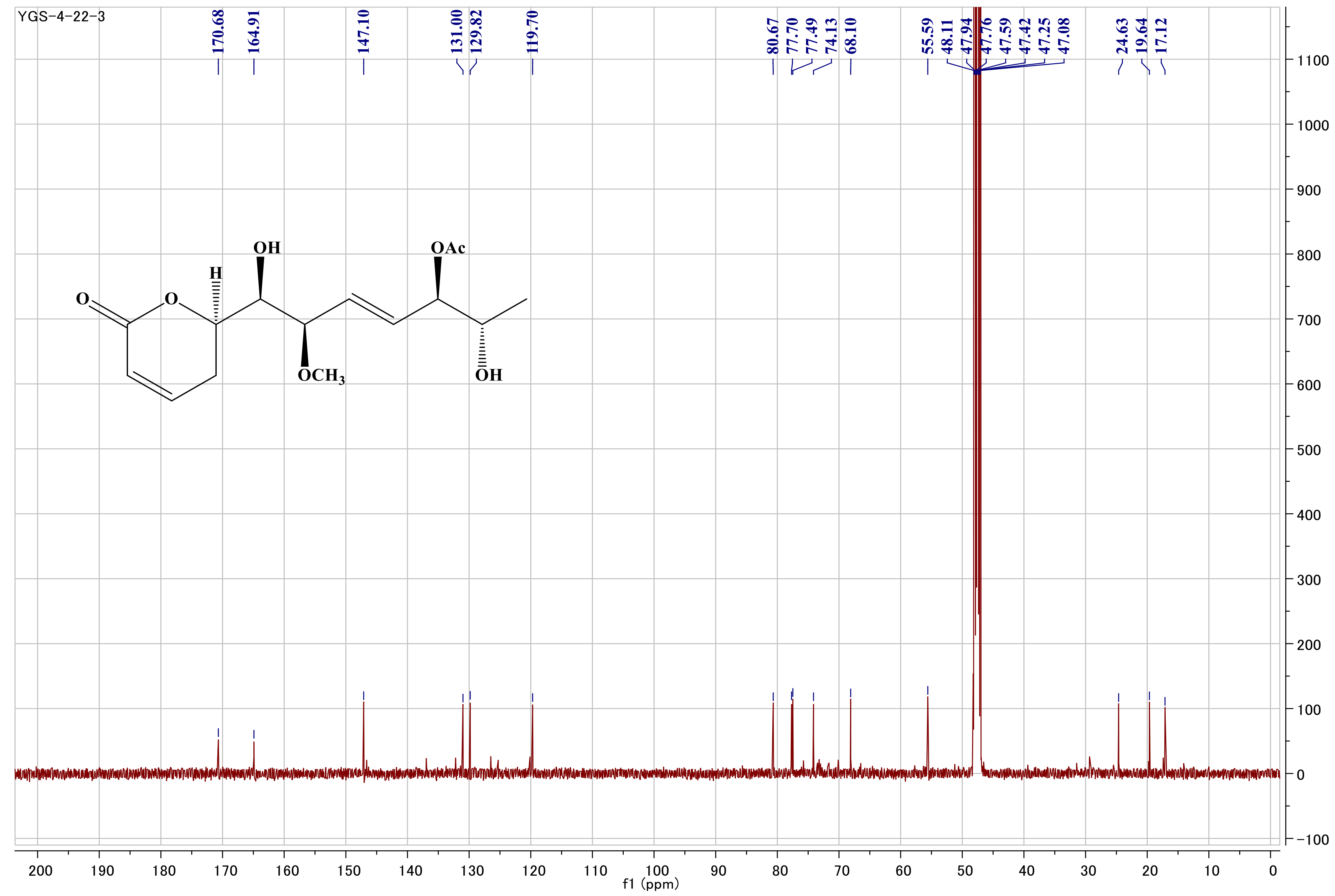

S18: ^{13}C NMR of **3**

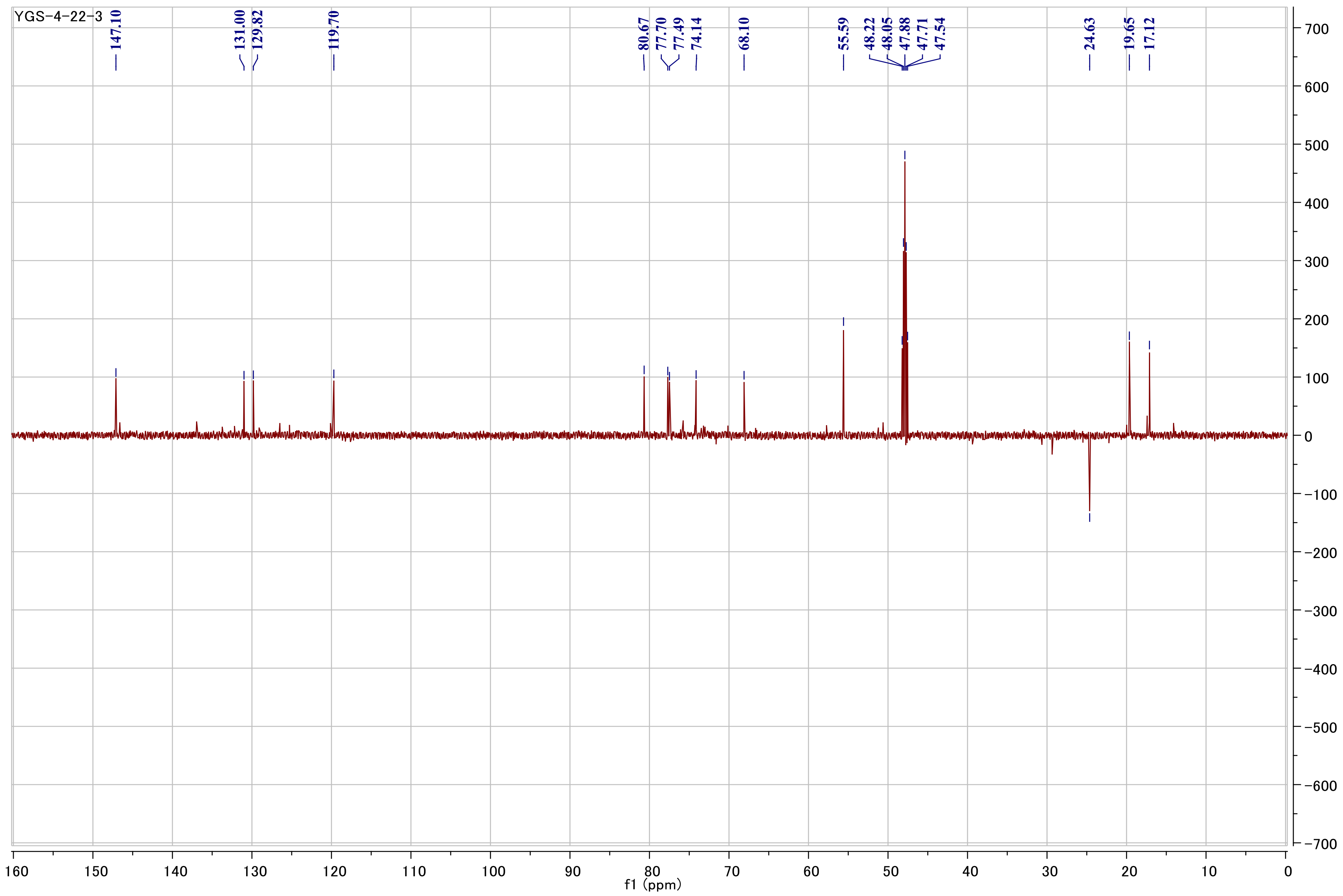

S19: DEPT-135 of **3**

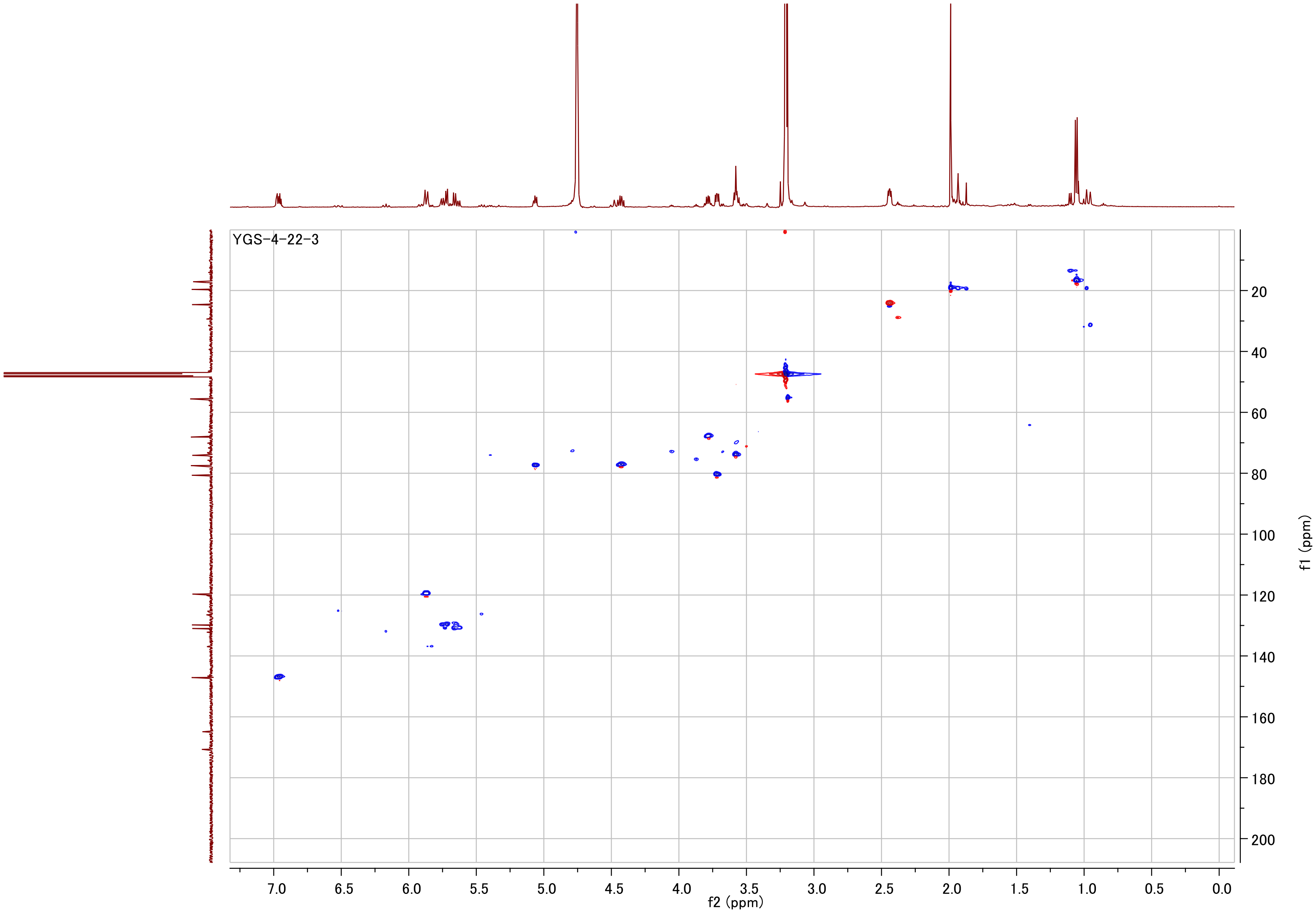

S20: HSQC of **3**

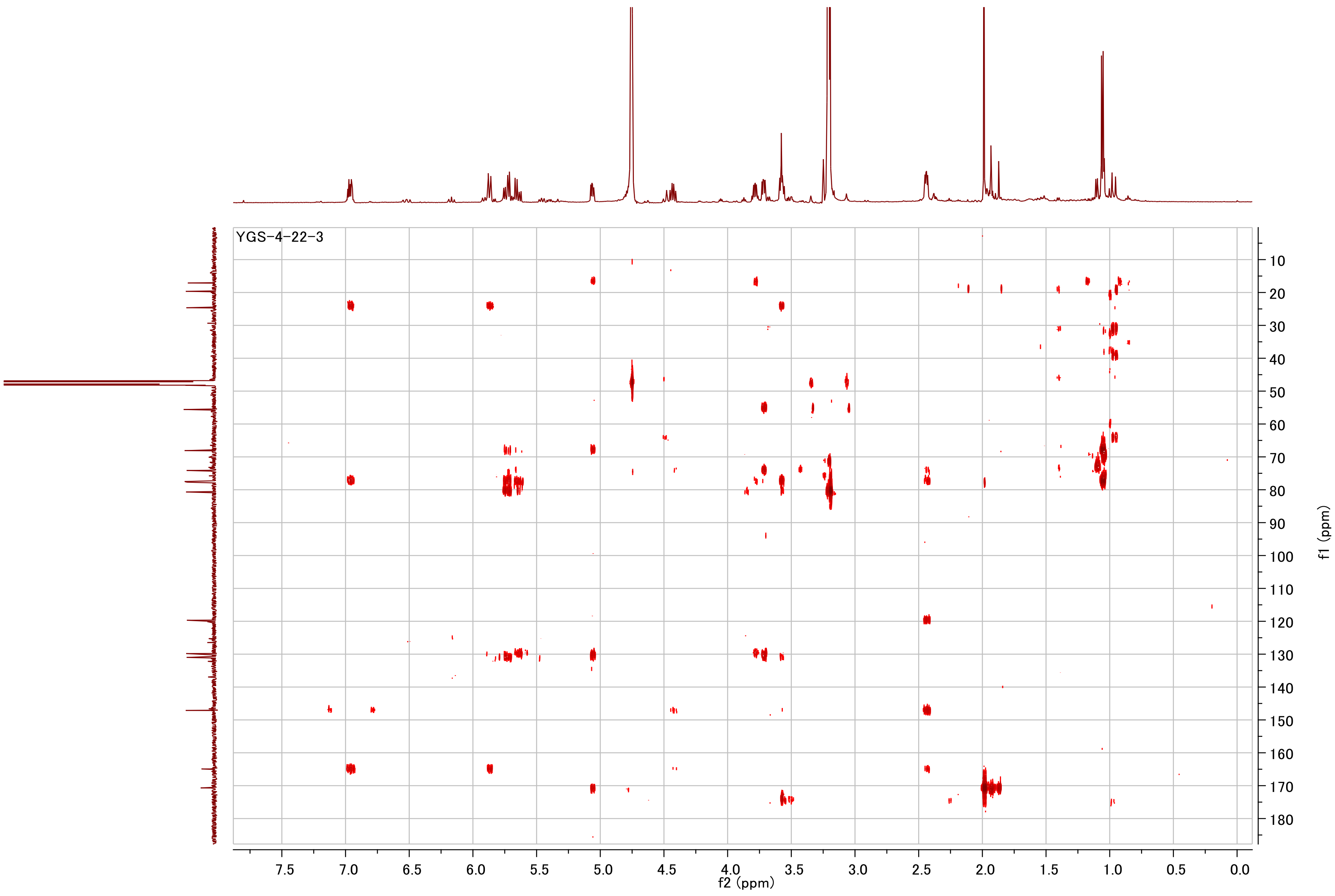
YGS-4-22-3
7.5
7.0
6.5
6.0
5.5
5.0
4.5
4.0
3.5
3.0
2.5
2.0
1.5
1.0
0.5
0.0
f2 (ppm)
10
20
30
40
50
60
70
80
90
100
110
120
130
140
150
160
170
180
f1 (ppm)

S21: HMBC of **3**

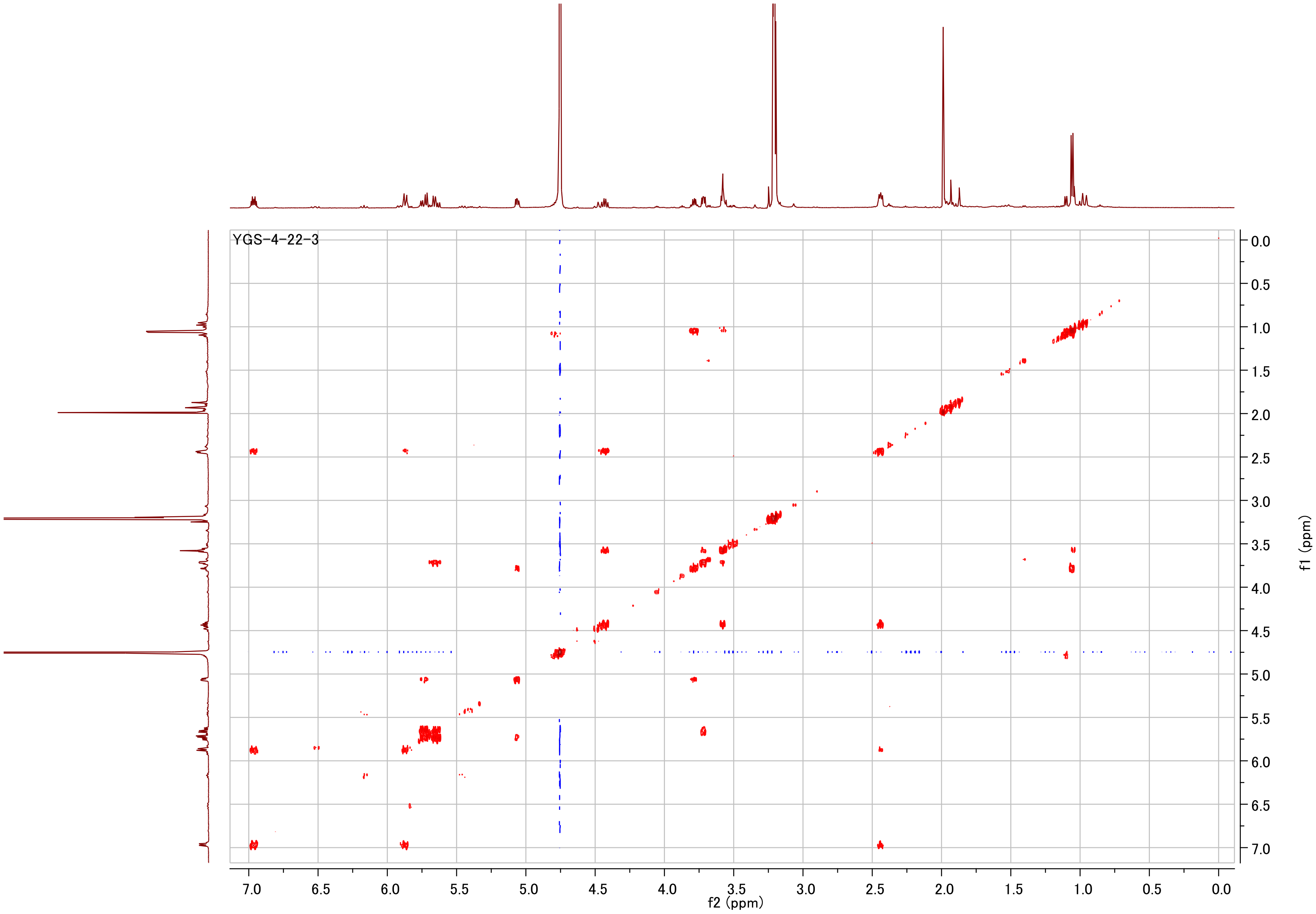

S22: ^{1}H ^{1}H COSY of **3**

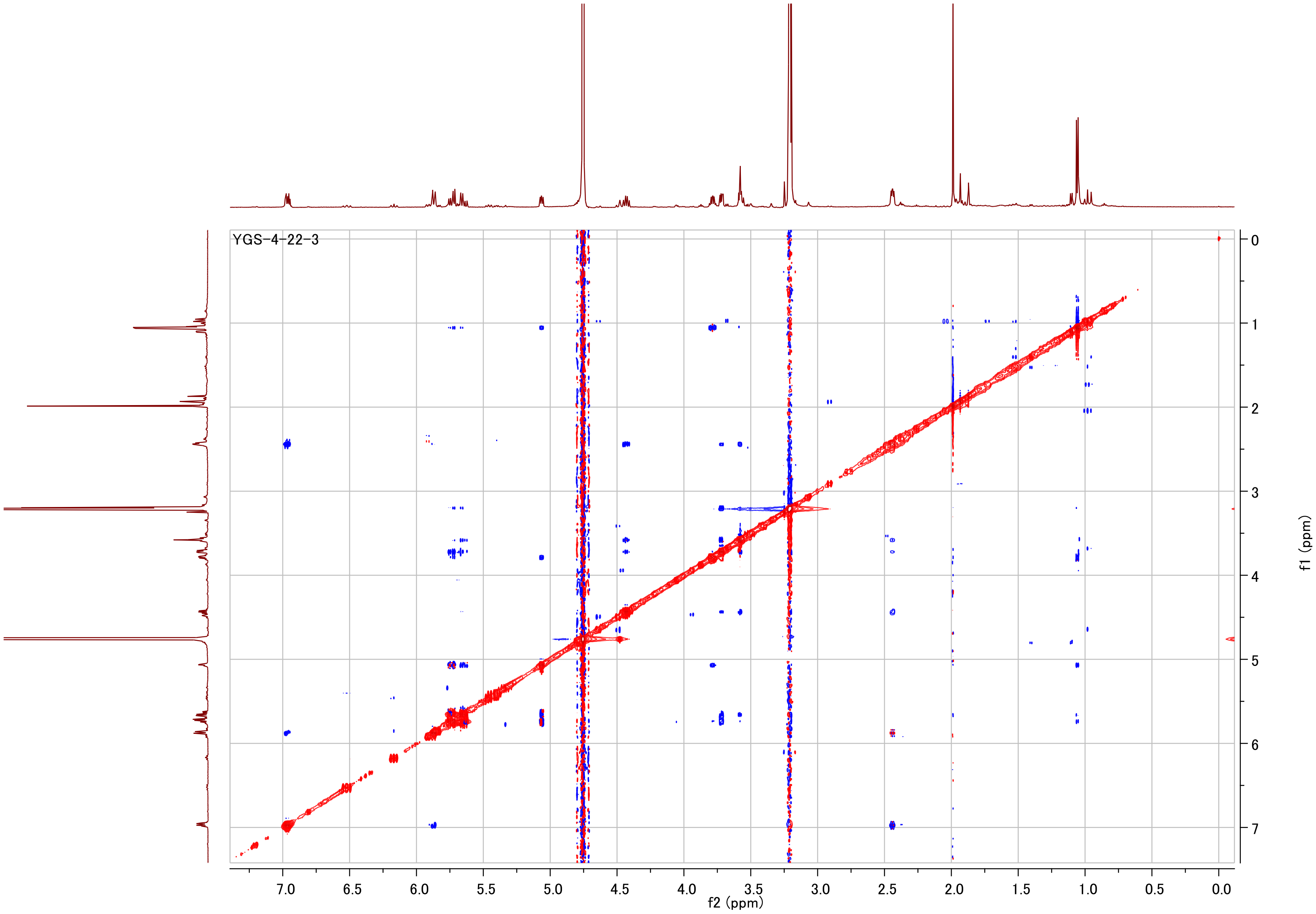

S23: NOESY of **3**

[Mass Spectrum]
Data : Umeyama-CI.26-Feb-2018.001 Date : 26-Feb-2018 13:33
Sample : YGS-4-22-2(CH4)
Note : MStation
Inlet : Direct Ion Mode : CI+
Spectrum Type : Normal Ion [MF-Linear]
RT : 1.56 min Scan# : 58-k(12)[k=1.0]
BP : m/z 95 Int. : 250.04 (2621856)
Output m/z range : 35 to 500 Cut Level : 0.00 %

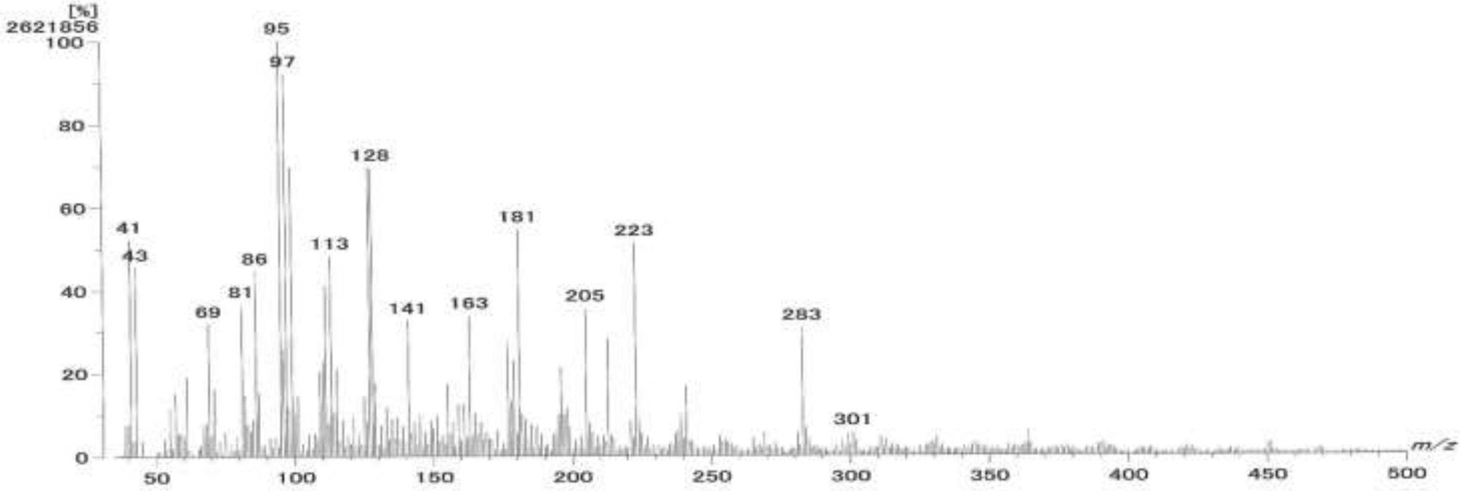

S24: LRCIMS of **4**

Data : Umeyama-CIHR.26-Feb-2018.002 Date : 26-Feb-2018 15:31
Instrument : MStation
Sample : YGS-4-22-2
Note : MStation
Inlet : Direct Ion Mode : CI+
RT : 1.00 min Scan# : 25
Elements : C 150/0, H 250/0, O 50/0
Mass Tolerance : 5mmu
Unsaturation (U.S.) : 0.0 – 15.0

	Observed m/z	Int%	Err[ppm / mmu]	U.S.	Composition
1	301.1265	2.59	+12.1 / +3.6	13.5	C21 H17 O2
2			-7.4 / -2.2	4.5	C14 H21 O7

S25: HRCIMS of **4**

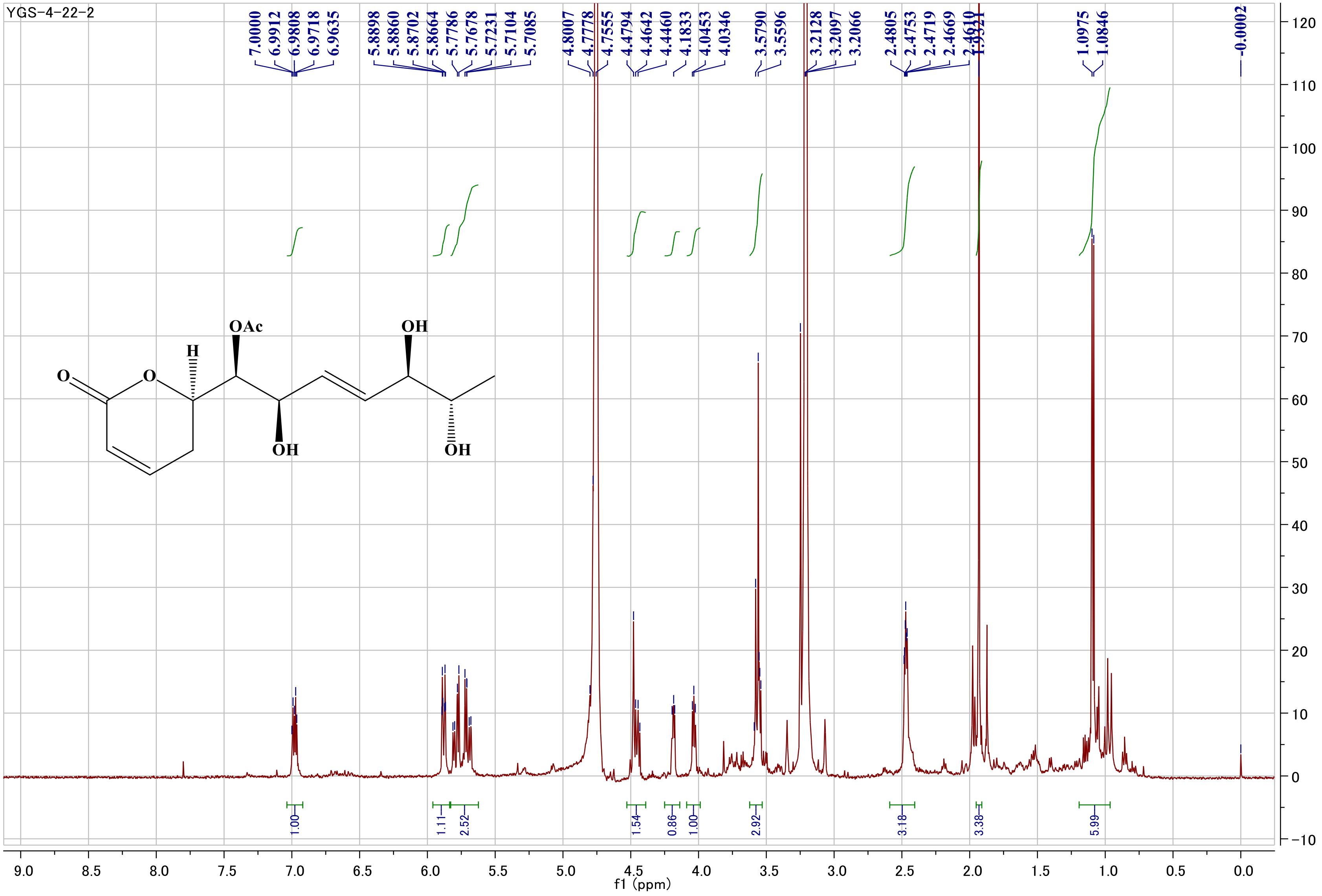

S26: ^{1}H NMR of **4**

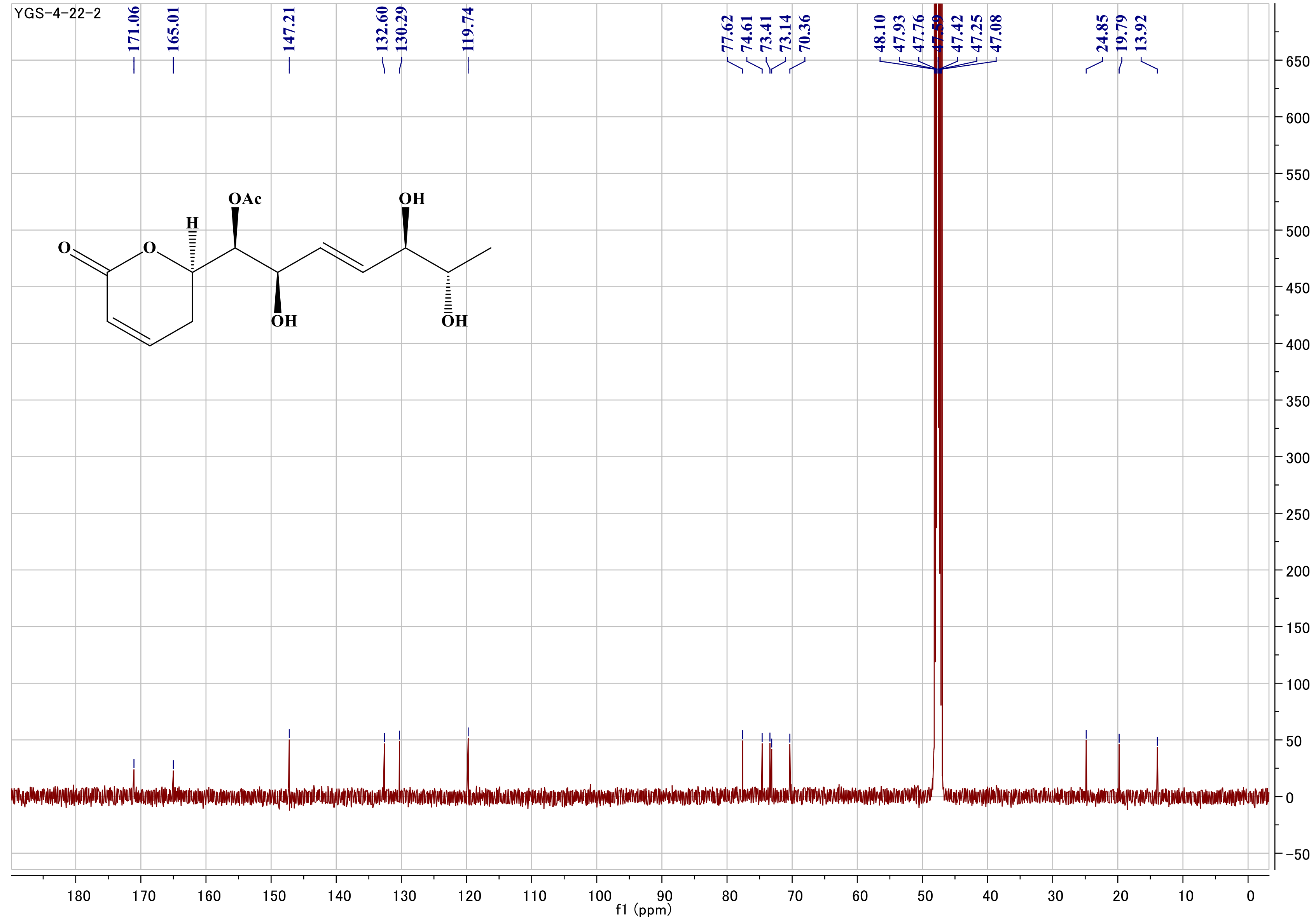

S27: ^{13}C NMR of **4**

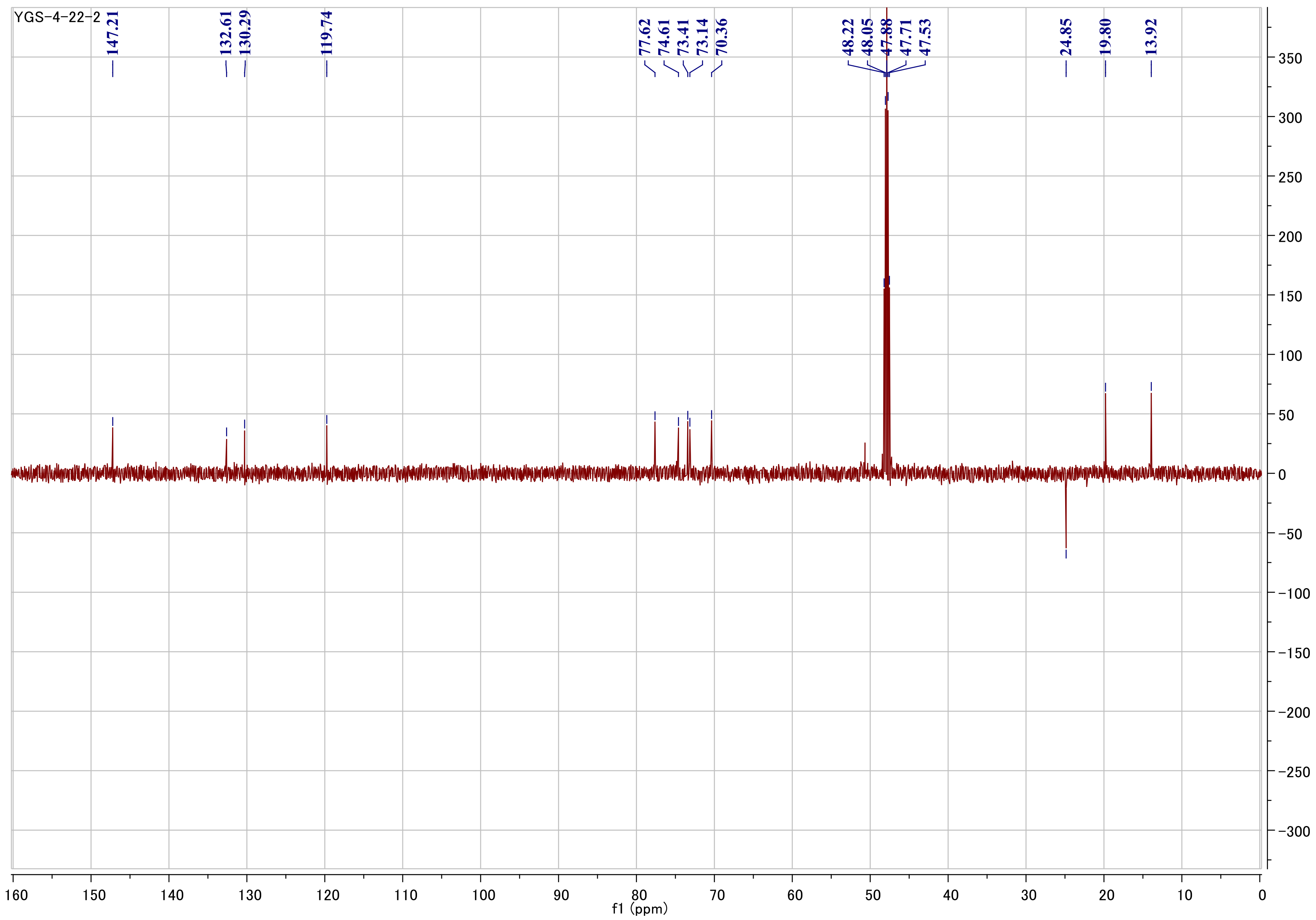

S28: DEPT-135 of **4**

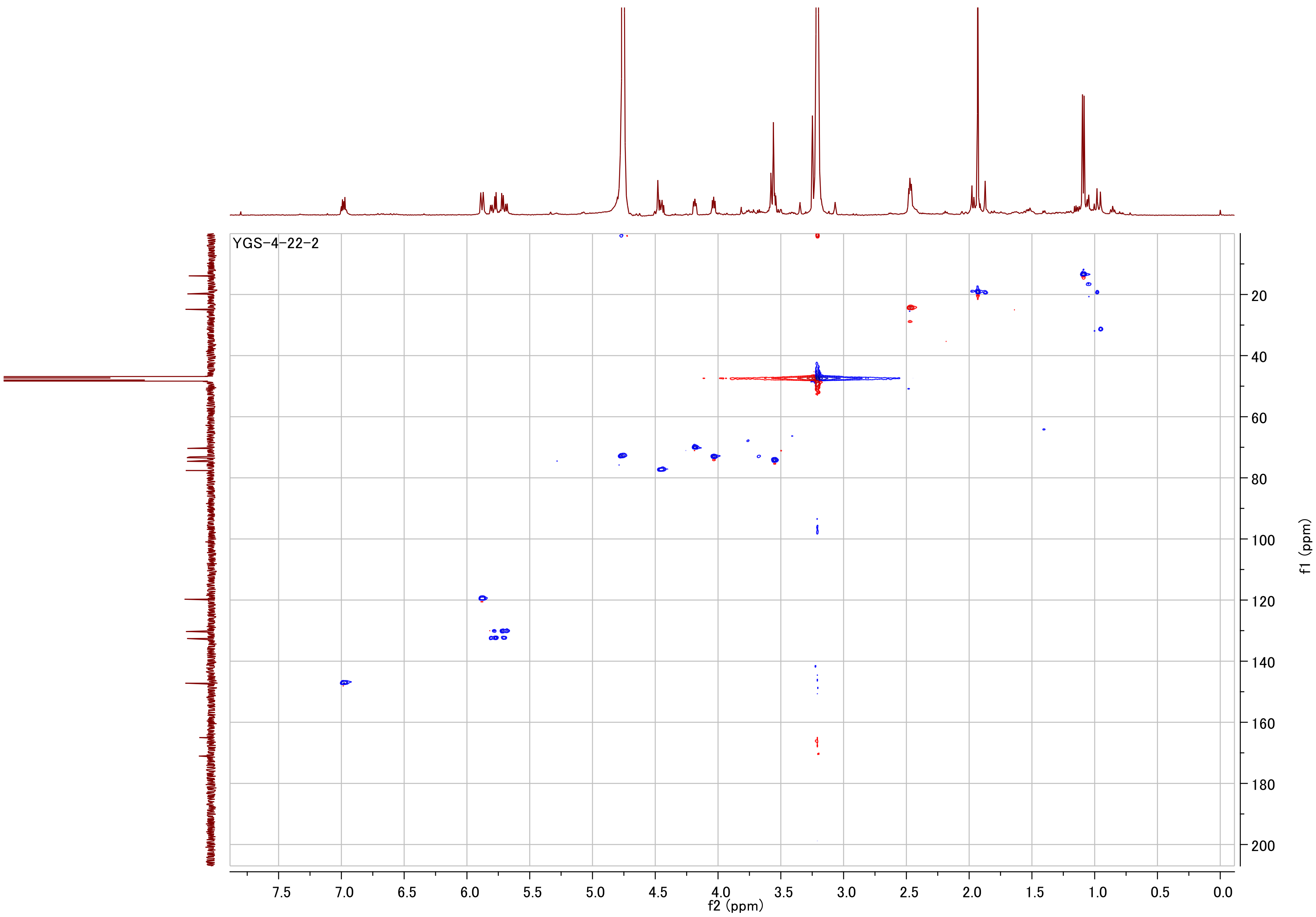

S29: HSQC of **4**

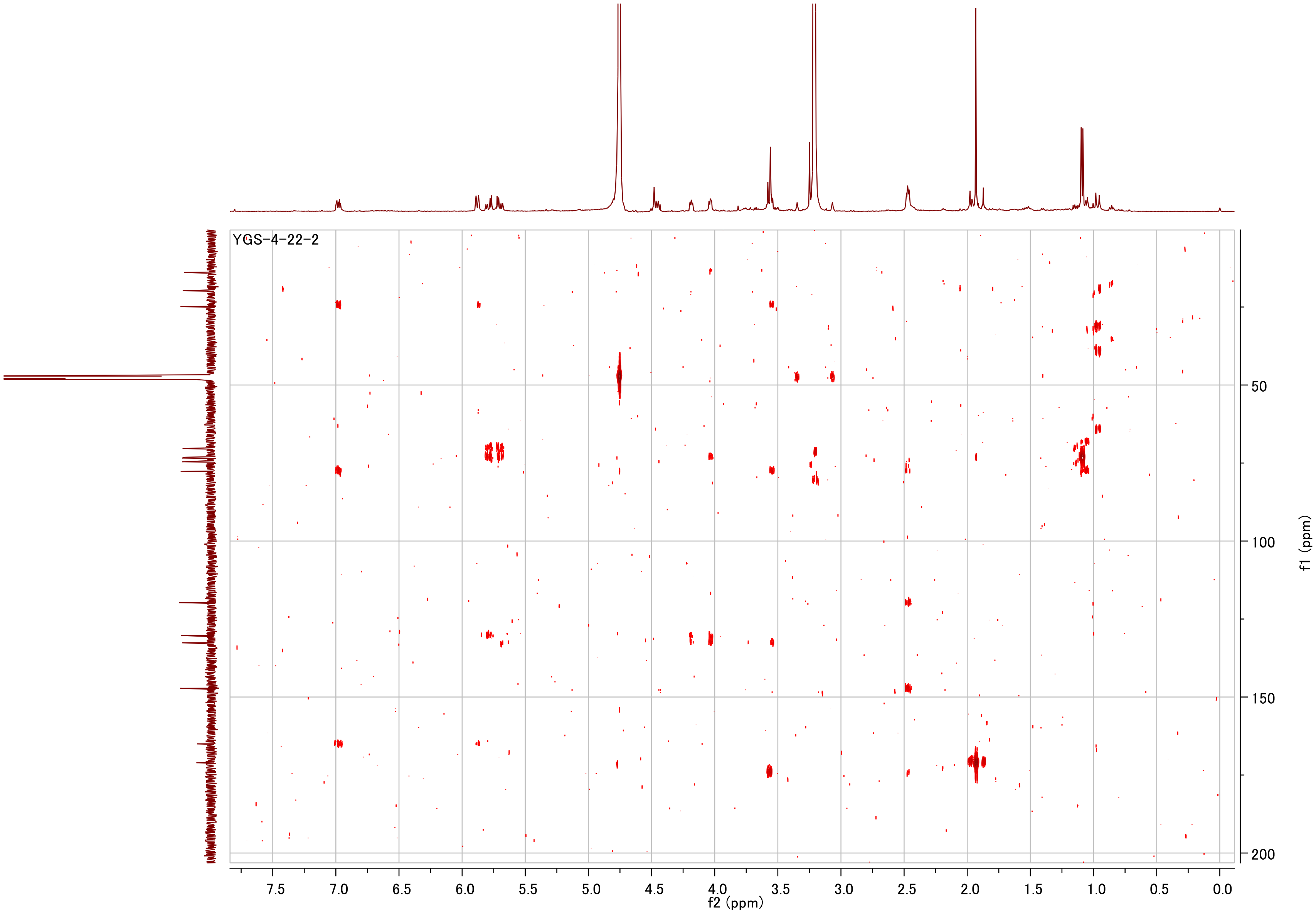
YGS-4-22-2
7.5
7.0
6.5
6.0
5.5
5.0
4.5
4.0
3.5
3.0
2.5
2.0
1.5
1.0
0.5
0.0
f2 (ppm)
50
100
150
200
f1 (ppm)

S30: HMBC of **4**

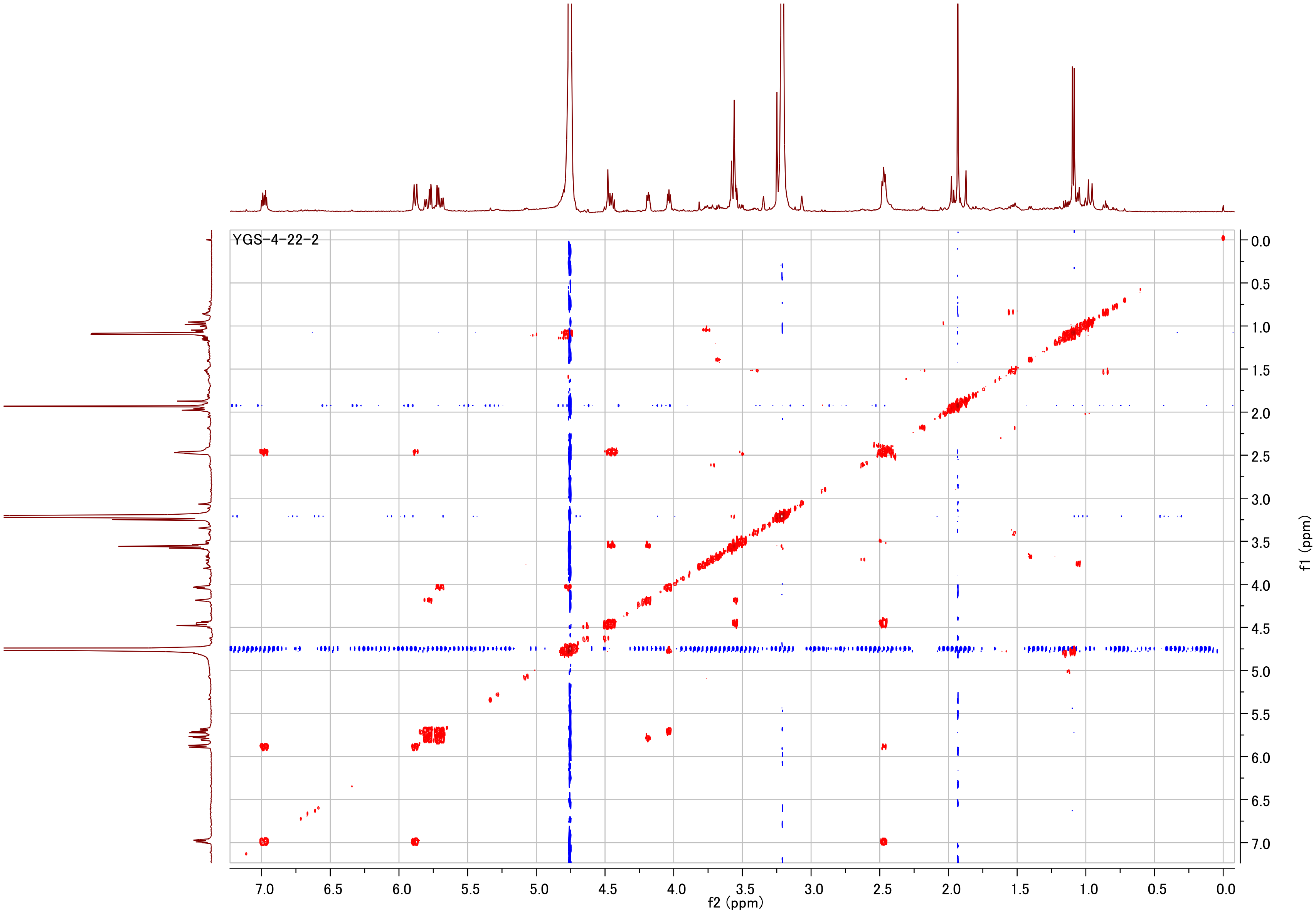

S31: ^{1}H ^{1}H COSY of **4**

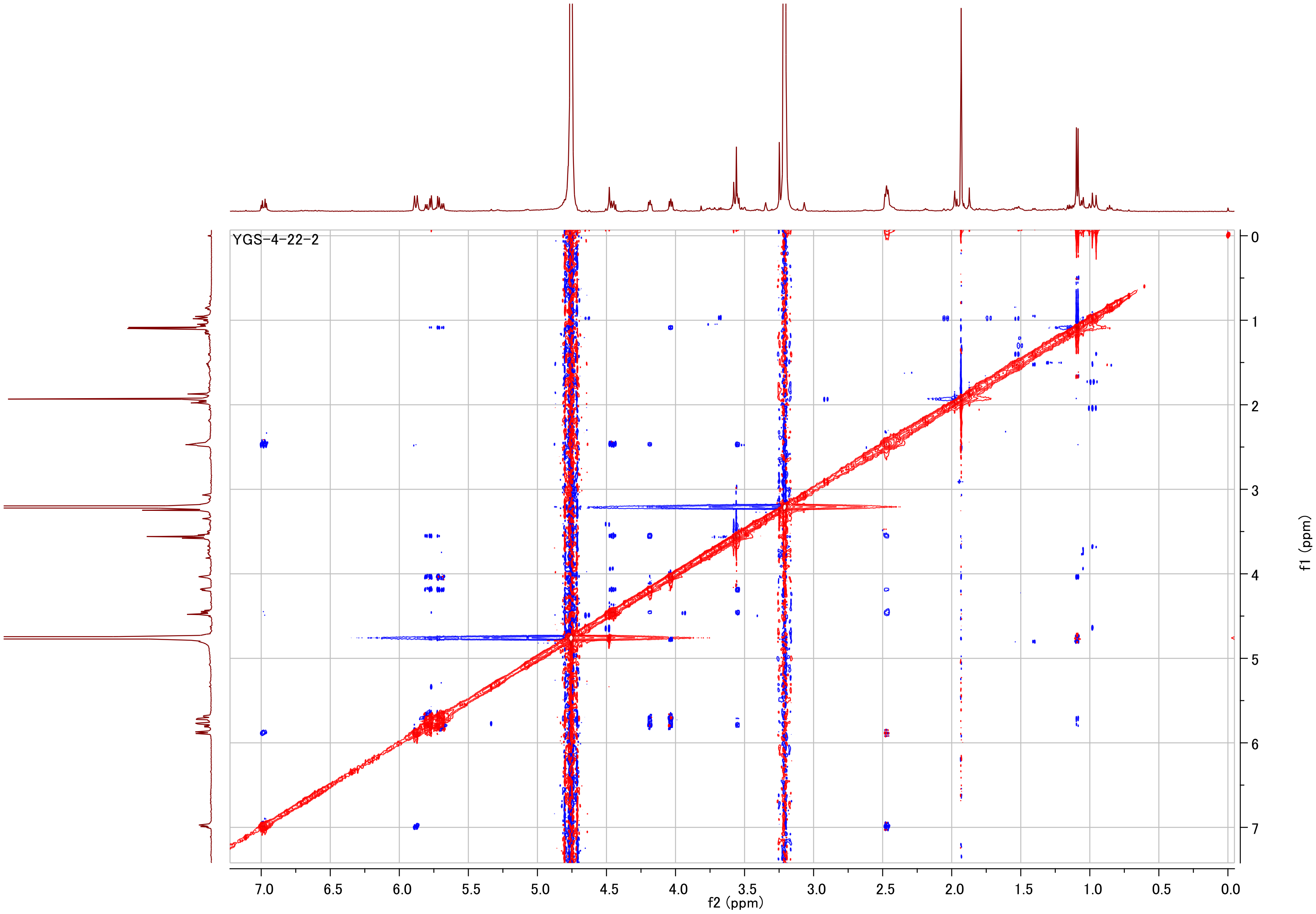

S32: NOESY of **4**

[Mass Spectrum]
Data : Umeyama-CI.07-Mar-2018.001 Date : 07-Mar-2018 11:41
Sample : YGS-4-22-1(CH4)
Note : MStation
Inlet : Direct Ion Mode : CI+
Spectrum Type : Normal Ion [MF-Linear]
RT : 1.45 min Scan# : 54
BP : m/z 127 Int. : 230.99 (2422144)
Output m/z range : 35 to 500 Cut Level : 0.00 %

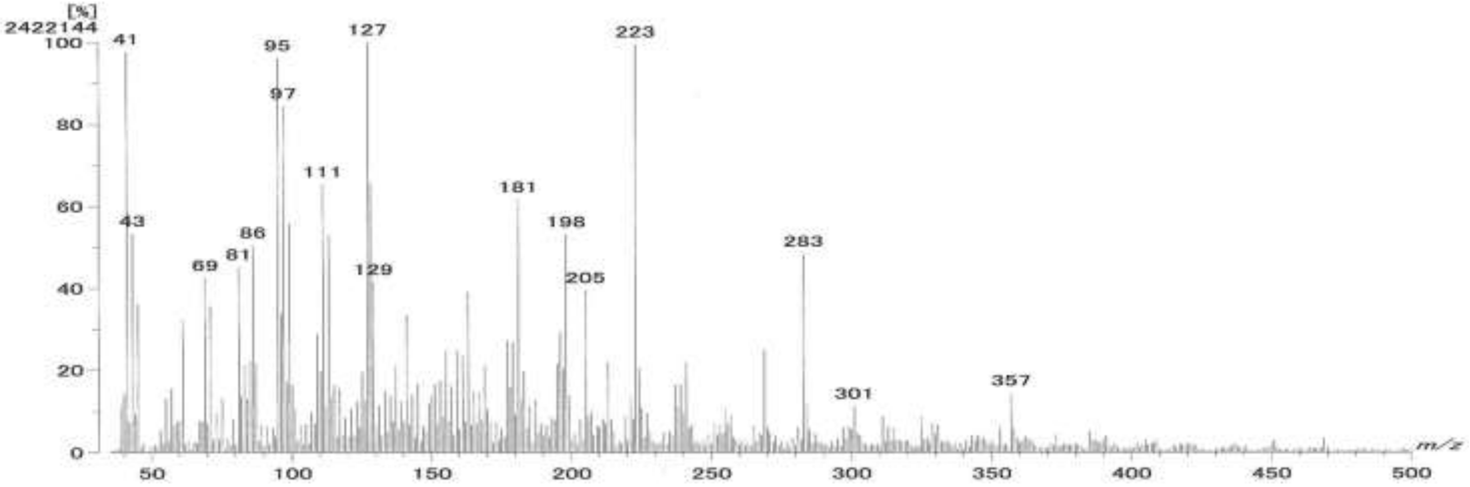

S33: LRCIMS of **5**

Data : Umeyama-CIHR.07-Mar-2018.002 Date : 07-Mar-2018 13:07
Instrument : MStation
Sample : YGS-4-22-1
Note : MStation
Inlet : Direct Ion Mode : CI+
RT : 1.54 min Scan# : 38
Elements : C 150/0, H 250/0, O 50/0
Mass Tolerance : 5mmu
Unsaturation (U.S.) : 0.0 - 15.0

	Observed m/z	Int%	Err[ppm / mmu]	U.S.	Composition
1	301.1269	13.63	+13.4 / +4.0	13.5	C21 H17 O2
2			-6.1 / -1.8	4.5	C14 H21 O7

S34: HRCIMS of **5**

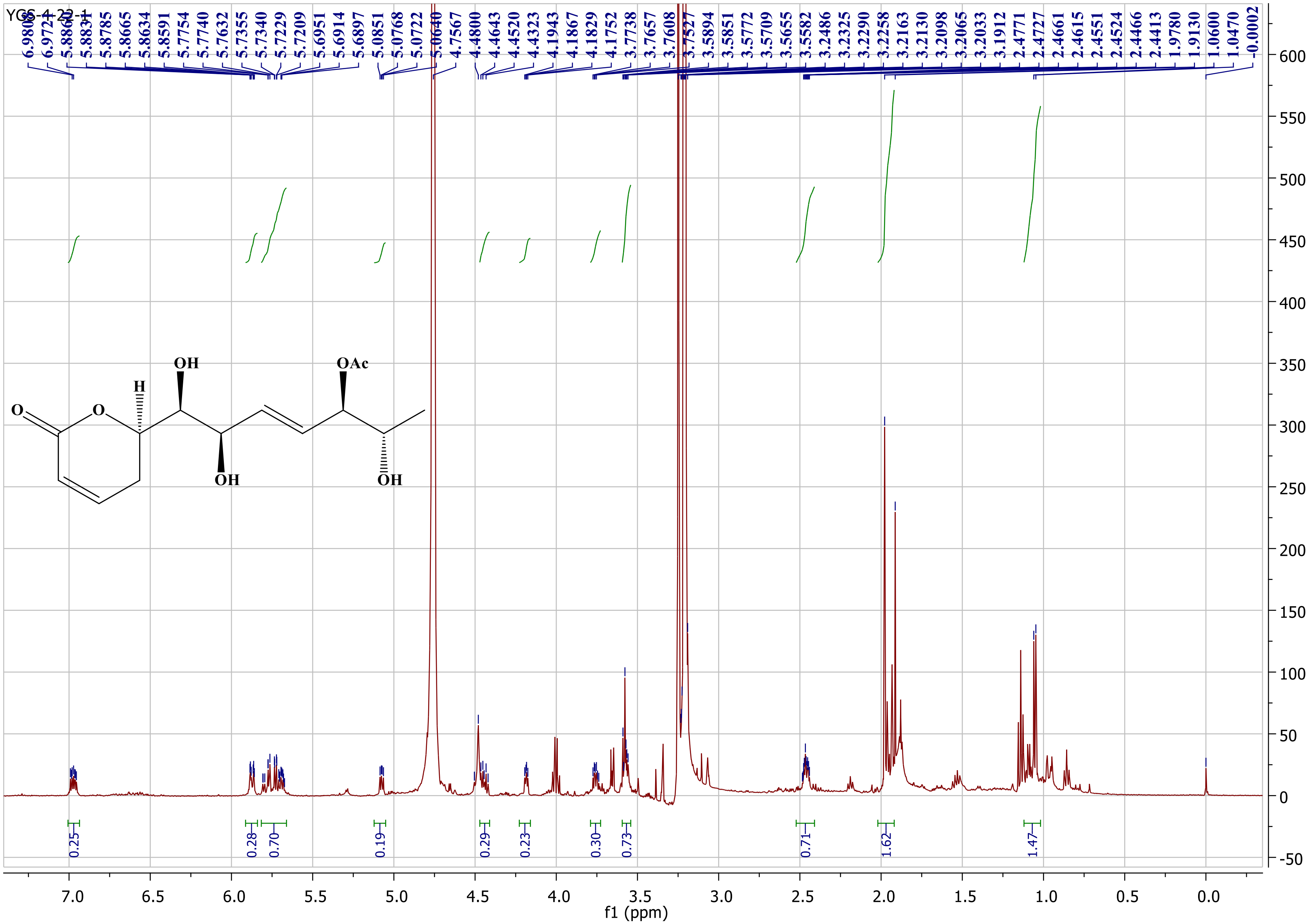

S35: ¹H NMR of **5**

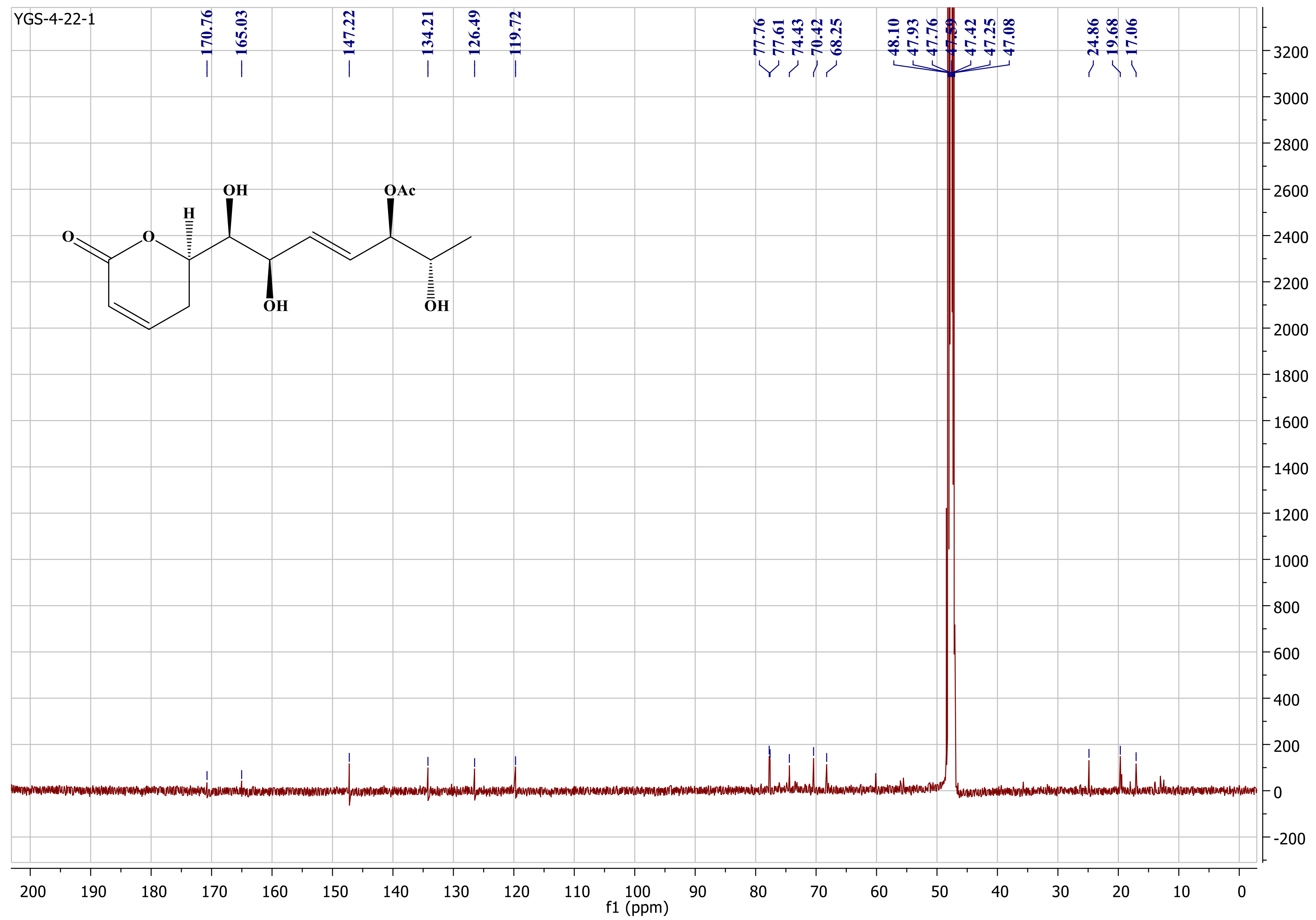

S36: ^{13}C NMR of **5**

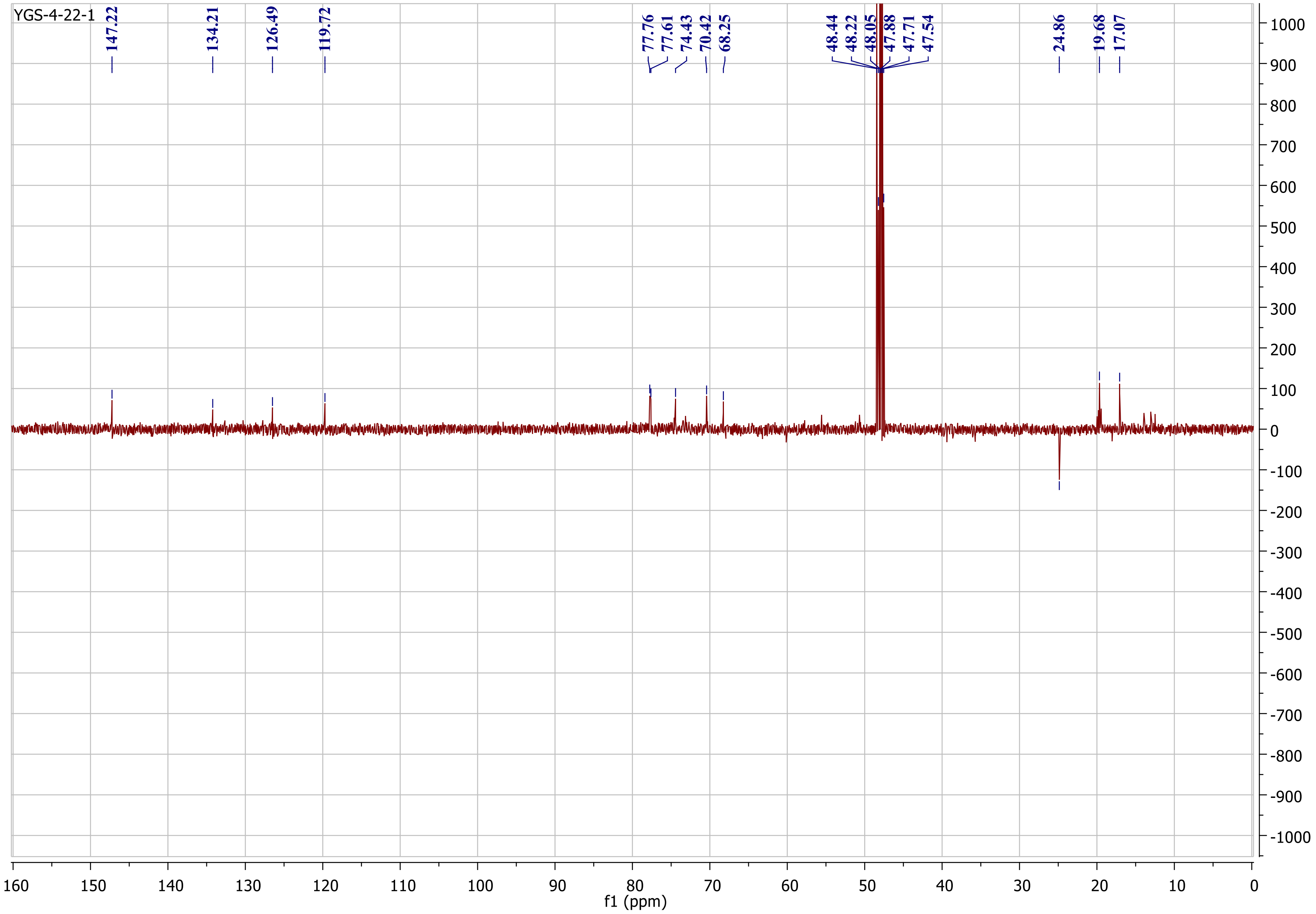

S37: DEPT-135 of **5**

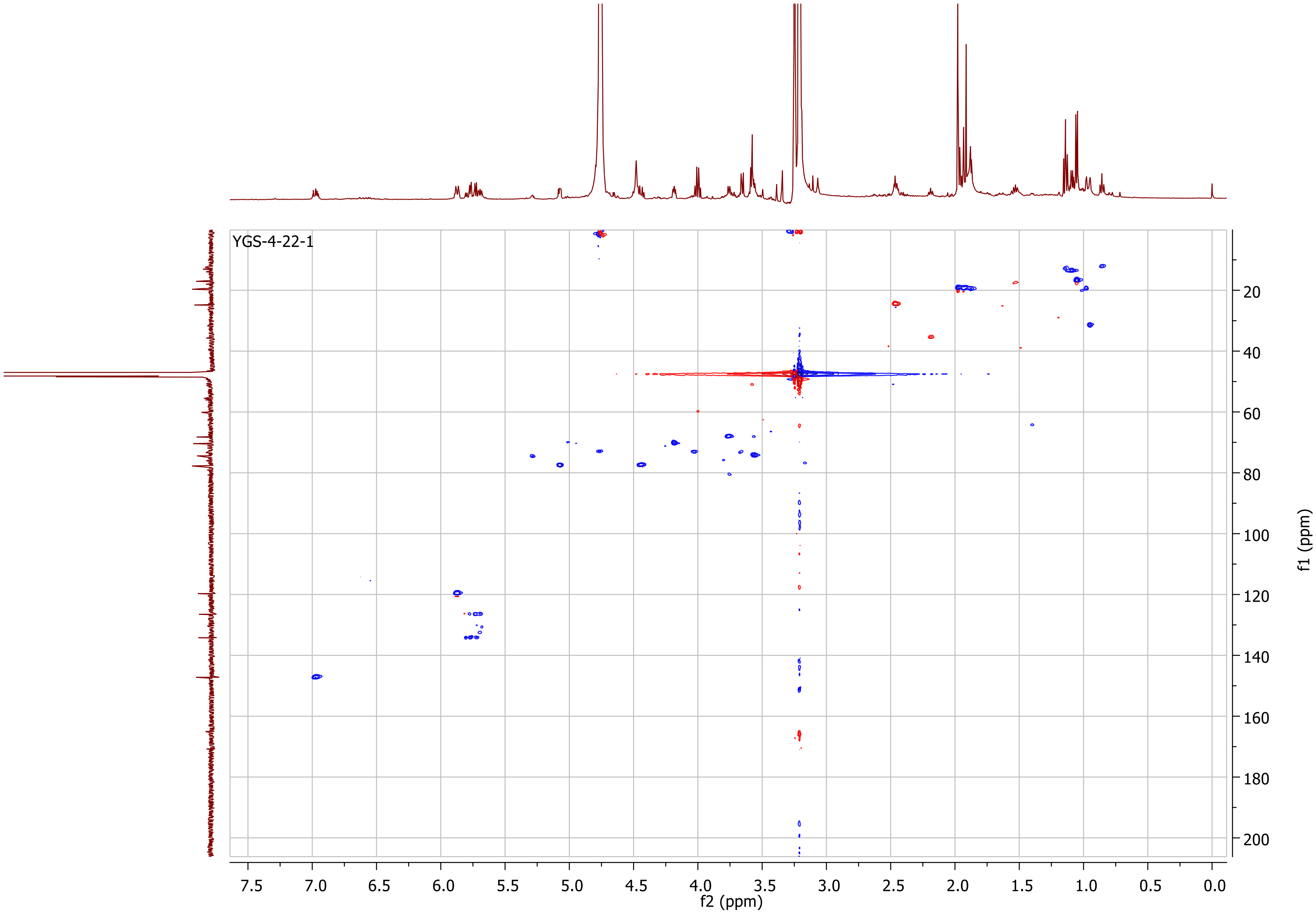
YGS-4-22-1
7.5
7.0
6.5
6.0
5.5
5.0
4.5
4.0
3.5
3.0
2.5
2.0
1.5
1.0
0.5
0.0
f2 (ppm)
20
40
60
80
100
120
140
160
180
200
f1 (ppm)

S38: HSQC of **5**

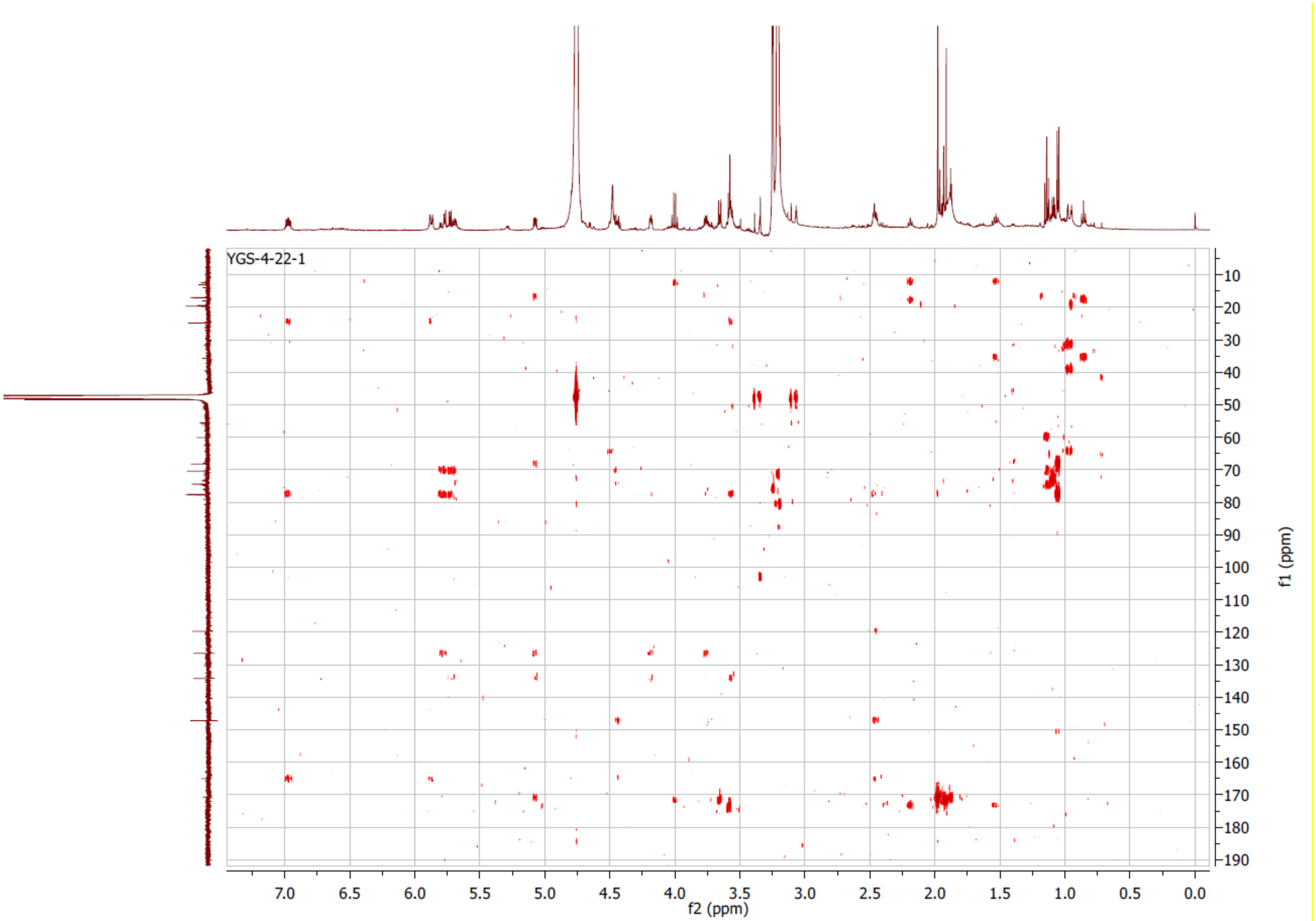

S39: HMBC of **5**

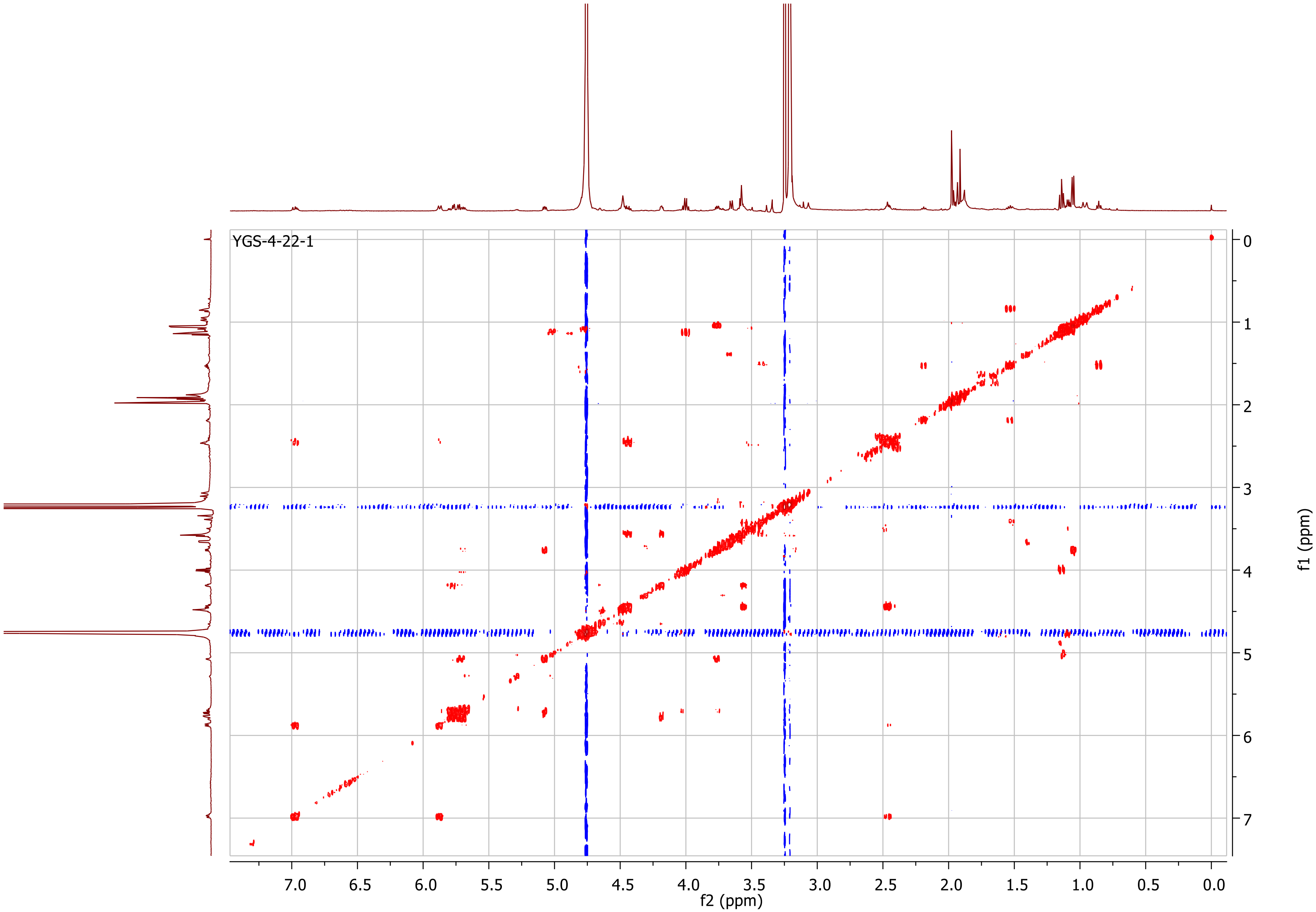

S40: ^{1}H ^{1}H COSY of **5**

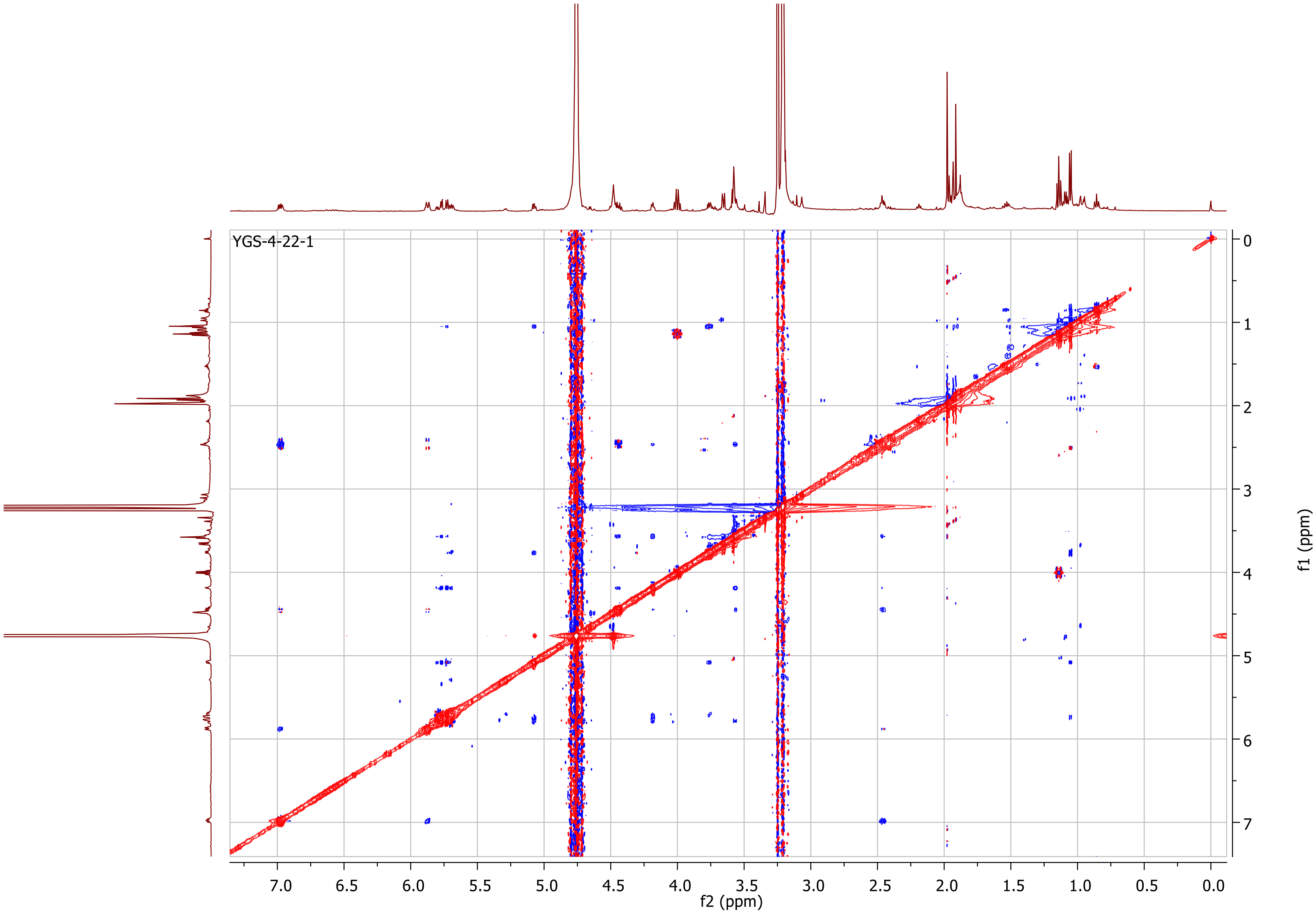

S41: NOESY of **5**

[Mass Spectrum]
Data : Umeyama-CL26-Feb-2018.003 Date : 26-Feb-2018 13:56
Sample : YGS-4-22-9(CH4)
Note : MStation
Inlet : Direct Ion Mode : CI+
Spectrum Type : Normal Ion [MF-Linear]
RT : 1.20 min Scan# : 45-k(13)[k=1.0]
BP : m/z 181 Int. : 399.60 (4190144)
Output m/z range : 35 to 500 Cut Level : 0.00 %

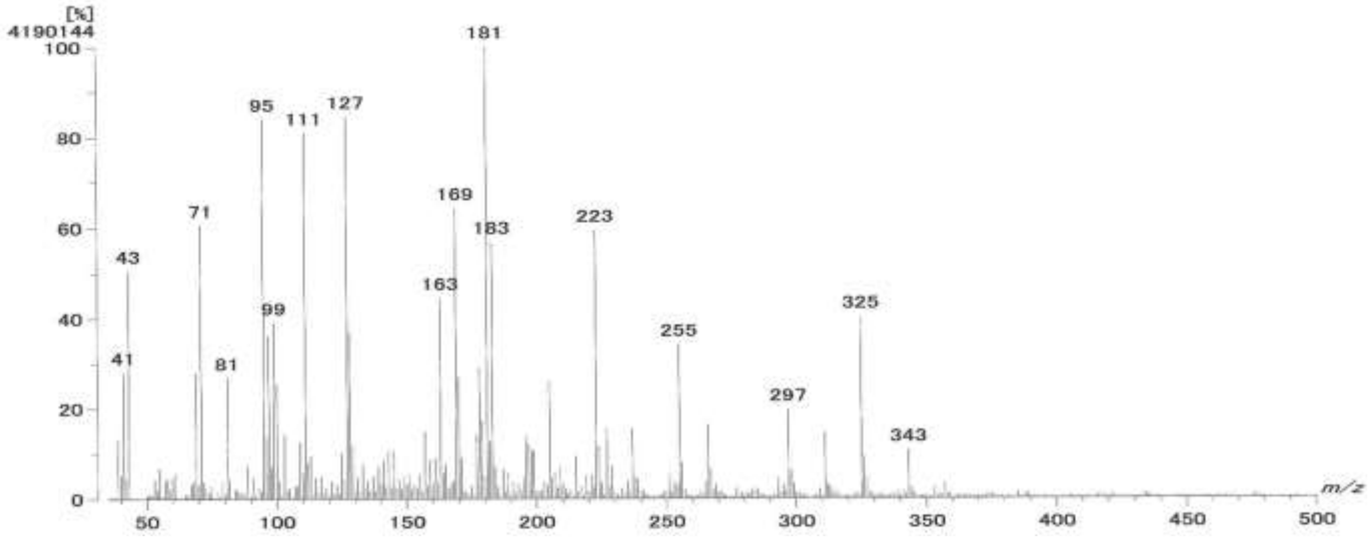

S42: LRCIMS of **6**

Data : Umeyama-CIHR.26-Feb-2018.004 Date : 26-Feb-2018 15:47
Instrument : MStation
Sample : YGS-4-22-9
Note : MStation
Inlet : Direct Ion Mode : CI+
RT : 1.04 min Scan# : 27
Elements : C 150/0, H 250/0, O 50/0
Mass Tolerance : 5mmu
Unsaturation (U.S.) : 0.0 – 15.0

	Observed m/z	Int%	Err[ppm / mmu]	U.S.	Composition
1	343.1755	12.81	-0.5 / -0.2	4.5	C17 H27 O7

S43: HRCIMS of **6**

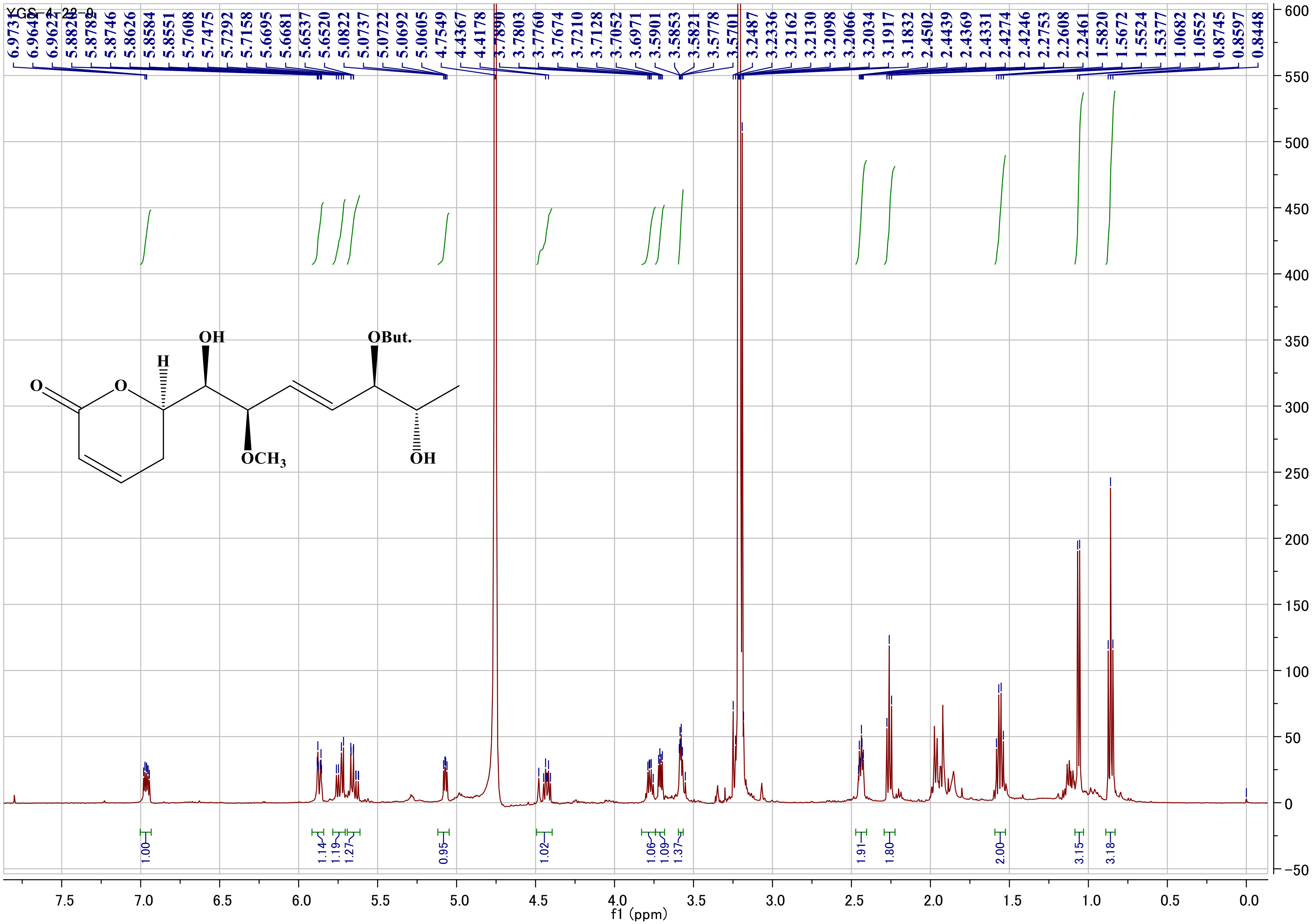

S44: ^{1}H NMR of **6**

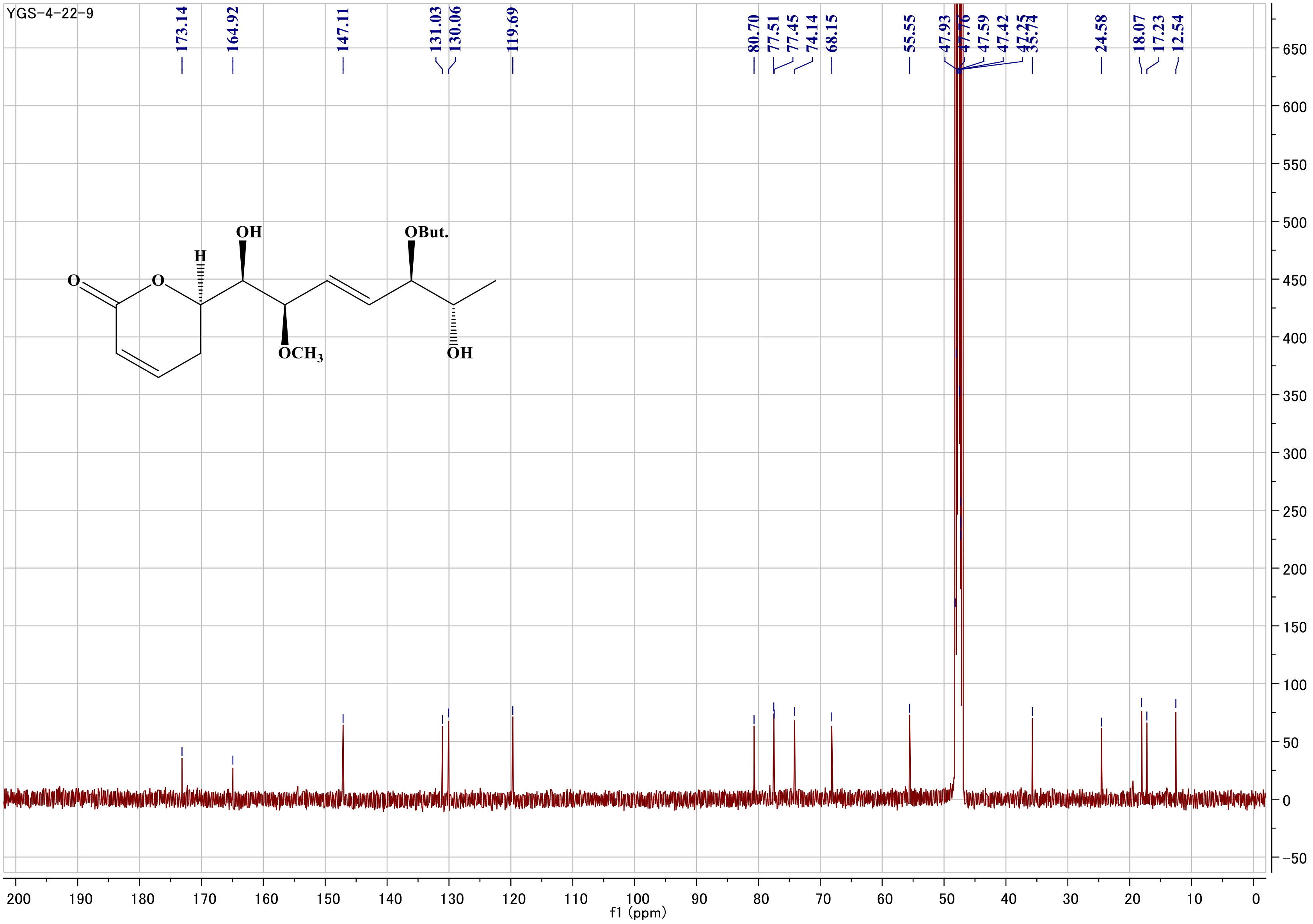

S45: ^{13}C NMR of **6**

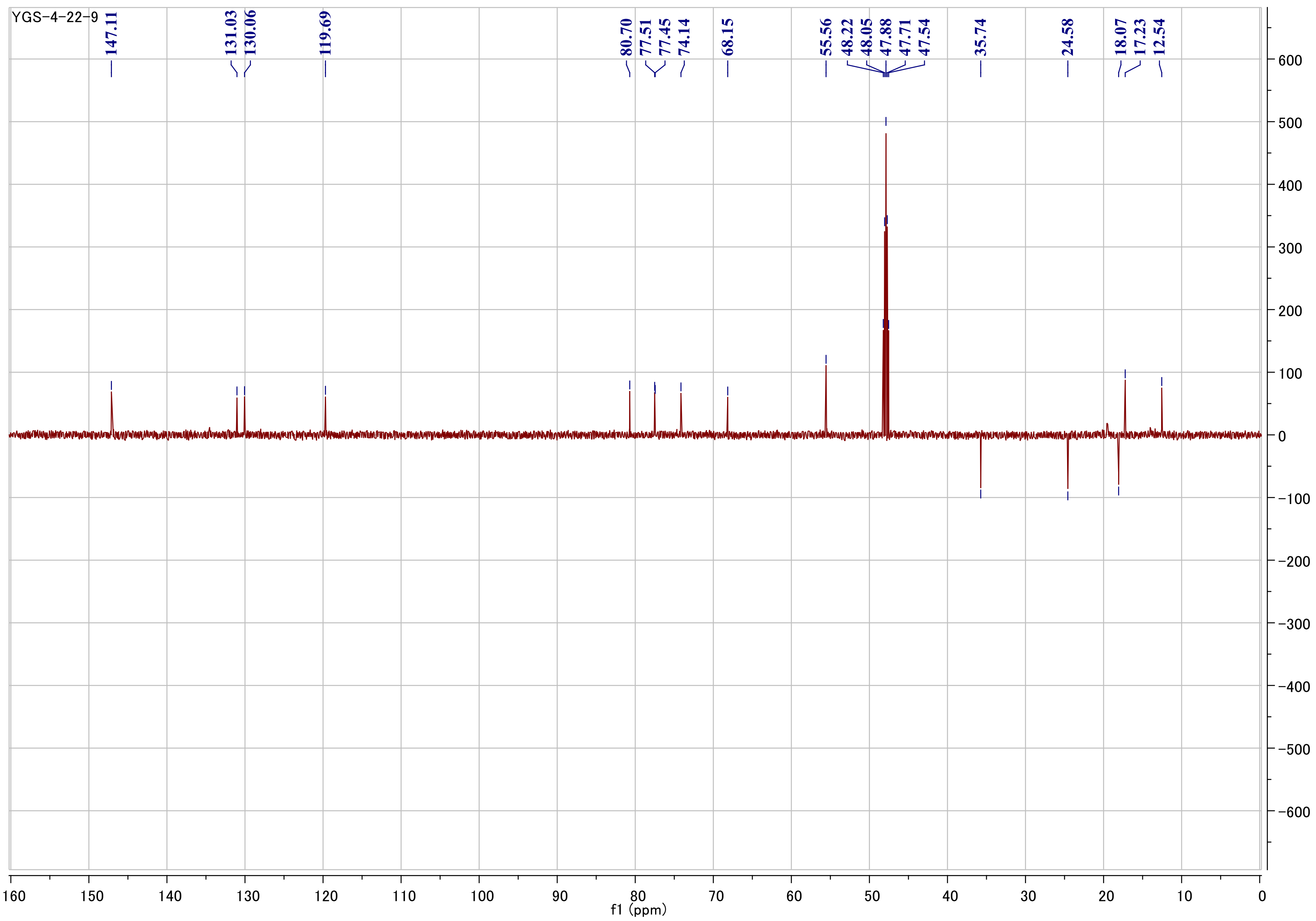

S46: DEPT-135 of **6**

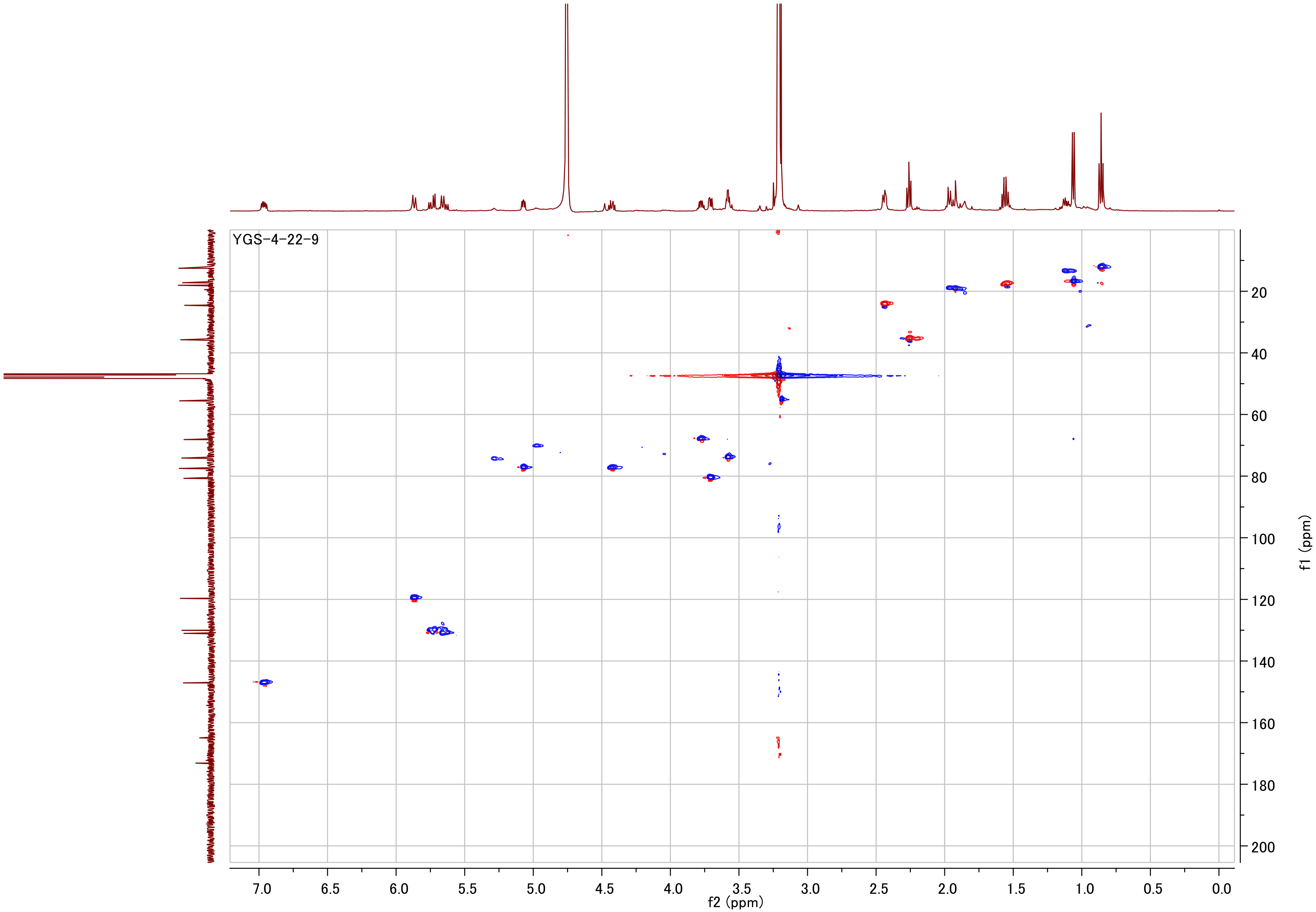

YGS-4-22-9
7.0
6.5
6.0
5.5
5.0
4.5
4.0
3.5
3.0
2.5
2.0
1.5
1.0
0.5
0.0
f2 (ppm)
20
40
60
80
100
120
140
160
180
200
f1 (ppm)

S47: HSQC of **6**

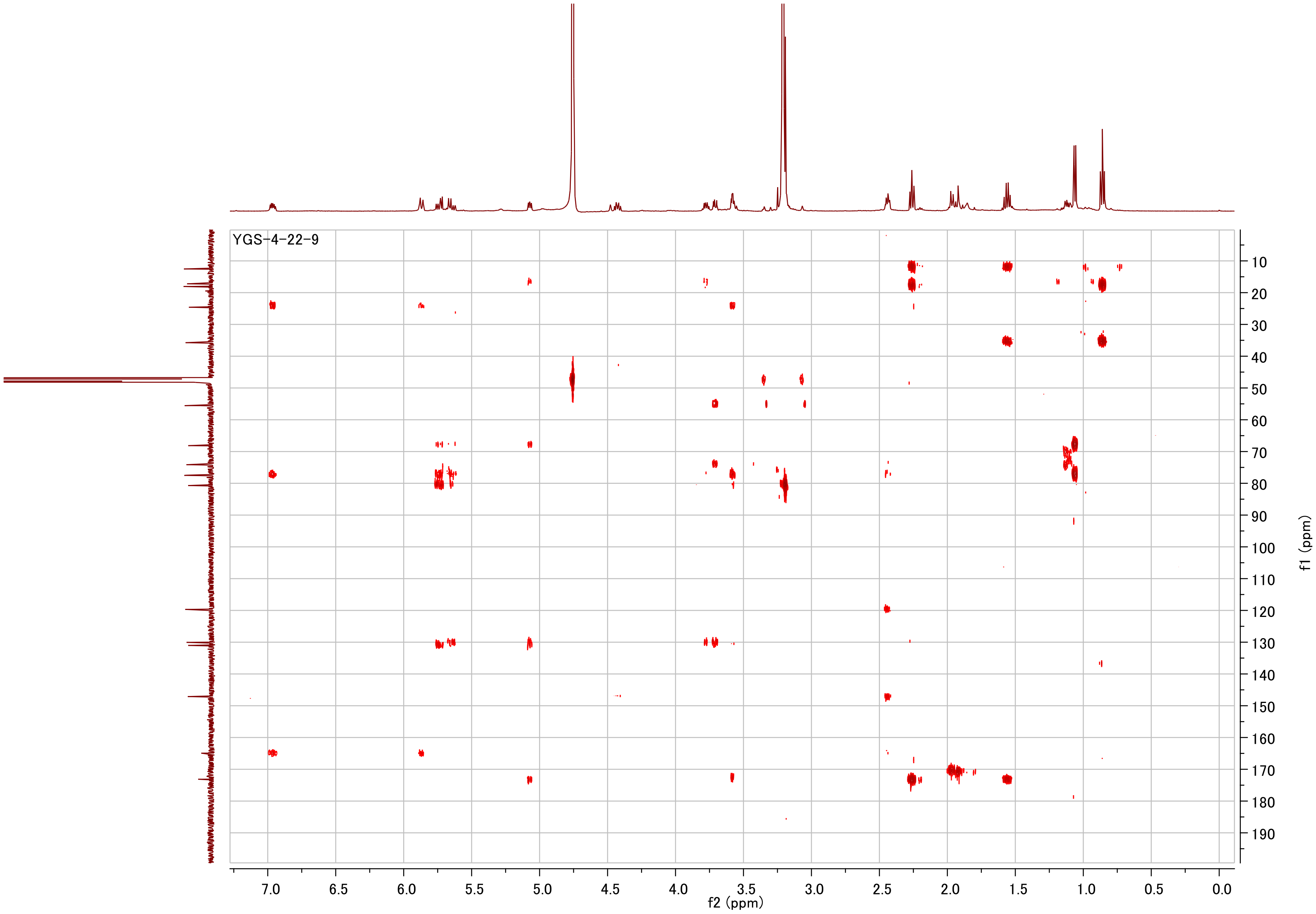
YGS-4-22-9
f2 (ppm)
f1 (ppm)
7.0
6.5
6.0
5.5
5.0
4.5
4.0
3.5
3.0
2.5
2.0
1.5
1.0
0.5
0.0
10
20
30
40
50
60
70
80
90
100
110
120
130
140
150
160
170
180
190

S48: HMBC of **6**

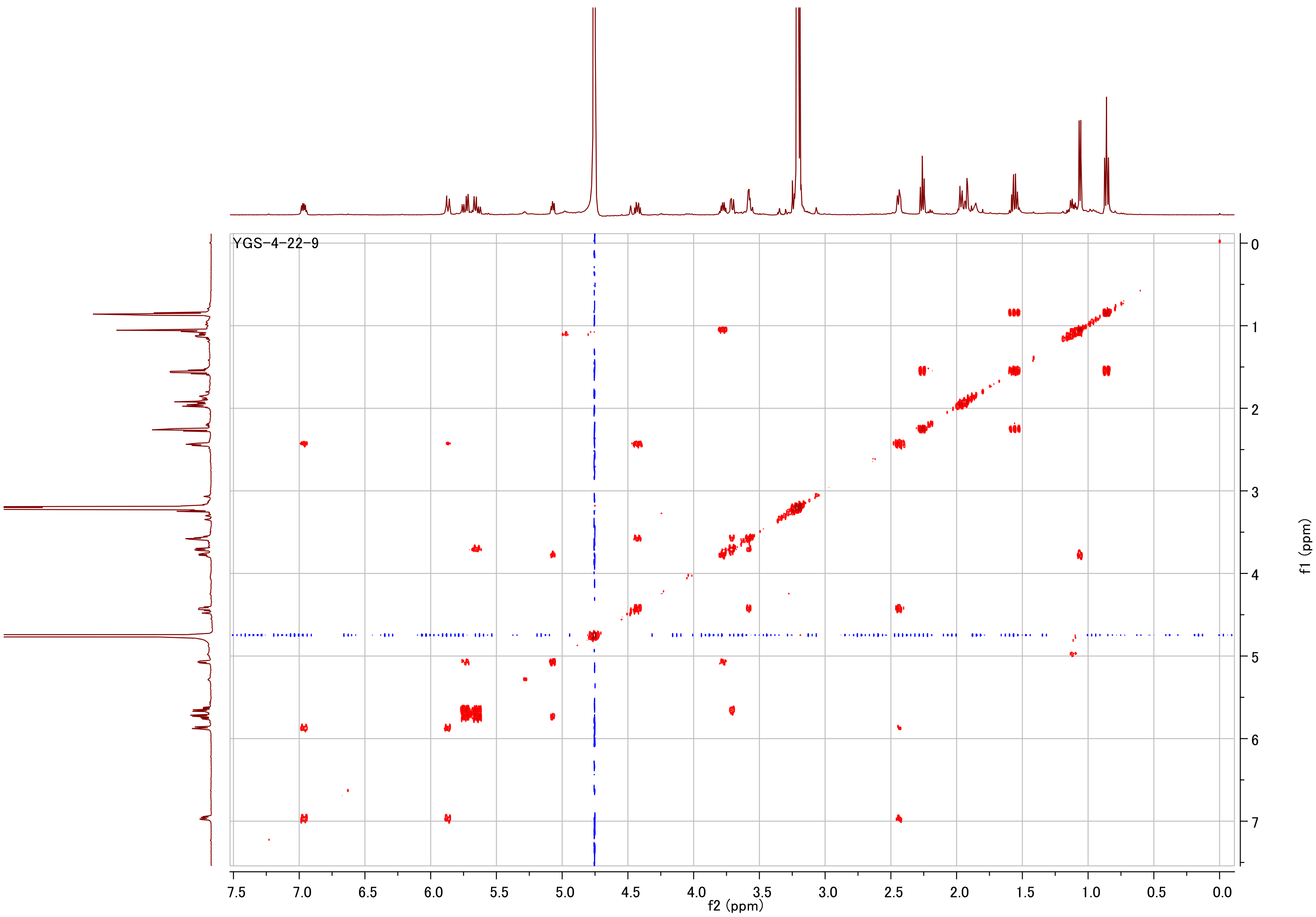

S49: ^{1}H ^{1}H COSY of **6**

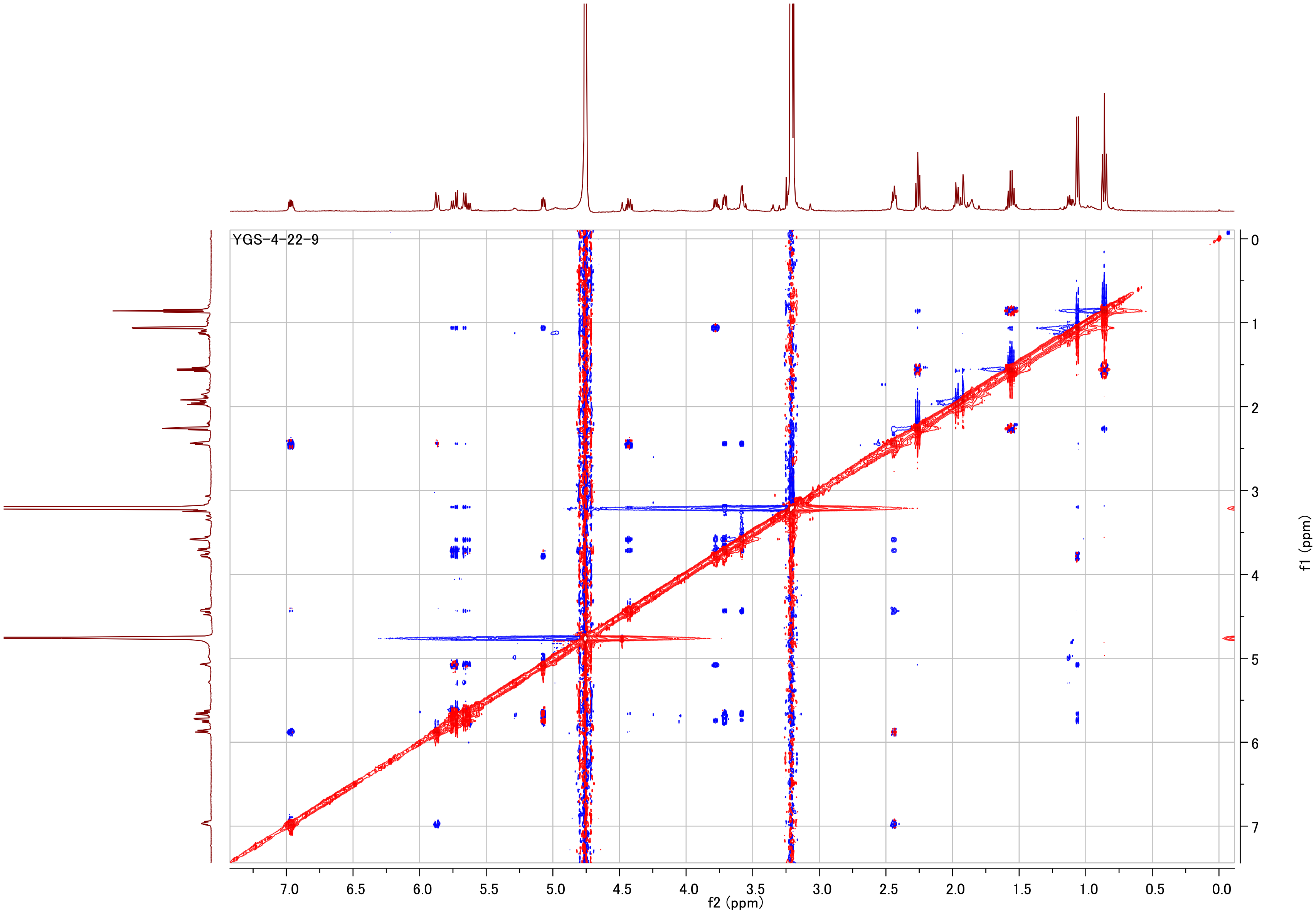

S50: NOESY of **6**

[Mass Spectrum]
Data : Umeyama-CI.15-Feb-2018.001 Date : 15-Feb-2018 10:36
Sample : YGS-4-22-14(CH4)
Note : MStation
Inlet : Direct Ion Mode : CI+
Spectrum Type : Normal Ion [MF-Linear]
RT : 1.48 min Scan# : 55
BP : m/z 311 Int. : 399.99 (4194240)
Output m/z range : 35 to 500 Cut Level : 0.00 %

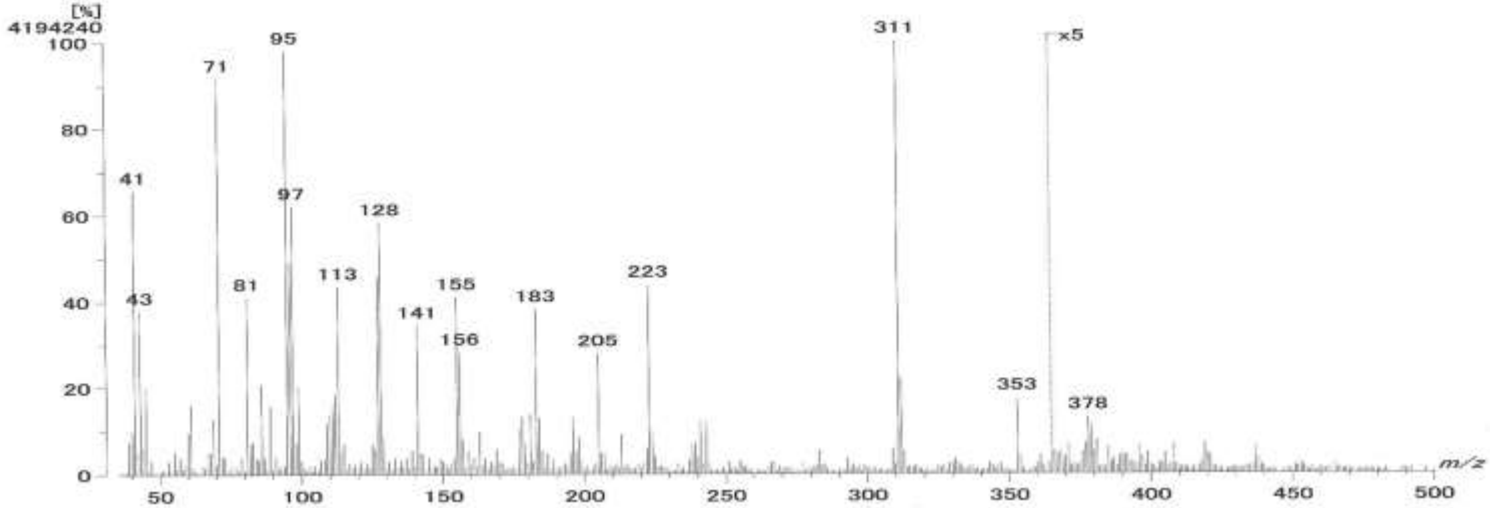

S51: LRCIMS of **7**

Data : Umeyama-CIHR.16-Feb-2018.001 Date : 16-Feb-2018 10:51
Instrument : MStation
Sample : YGS-4-22-14
Note : MStation
Inlet : Direct Ion Mode : CI+
RT : 1.80 min Scan# : 46
Elements : C 150/0, H 250/0, O 50/0
Mass Tolerance : 5mmu
Unsaturation (U.S.) : 0.0 – 15.0

	Observed m/z	Int%	Err[ppm / mmu]	U.S.	Composition
1	371.1705	13.98	-0.3 / -0.1	5.5	C18 H27 O8

S52: HRCIMS of **7**

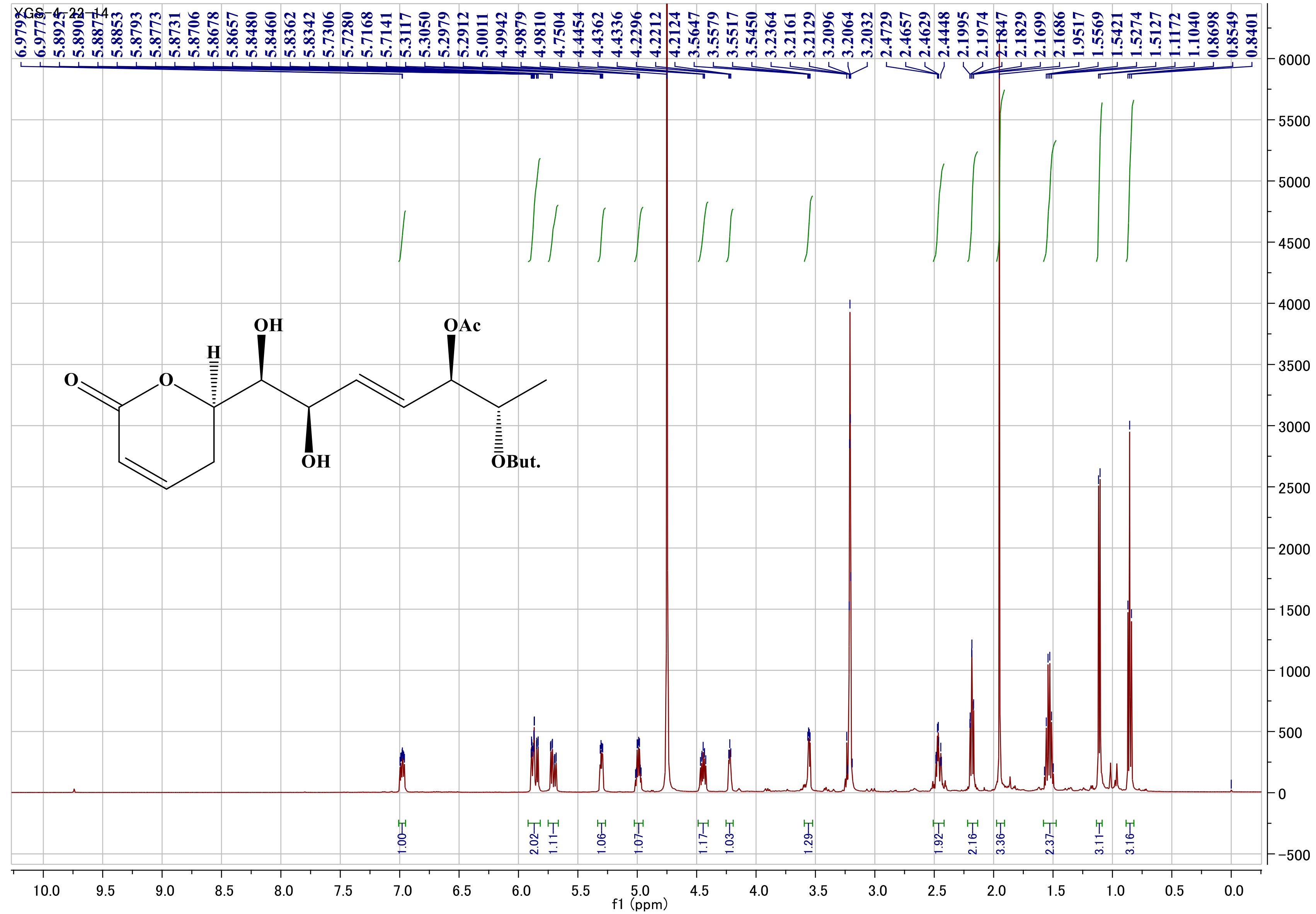

S53: ^{1}H NMR of **7**

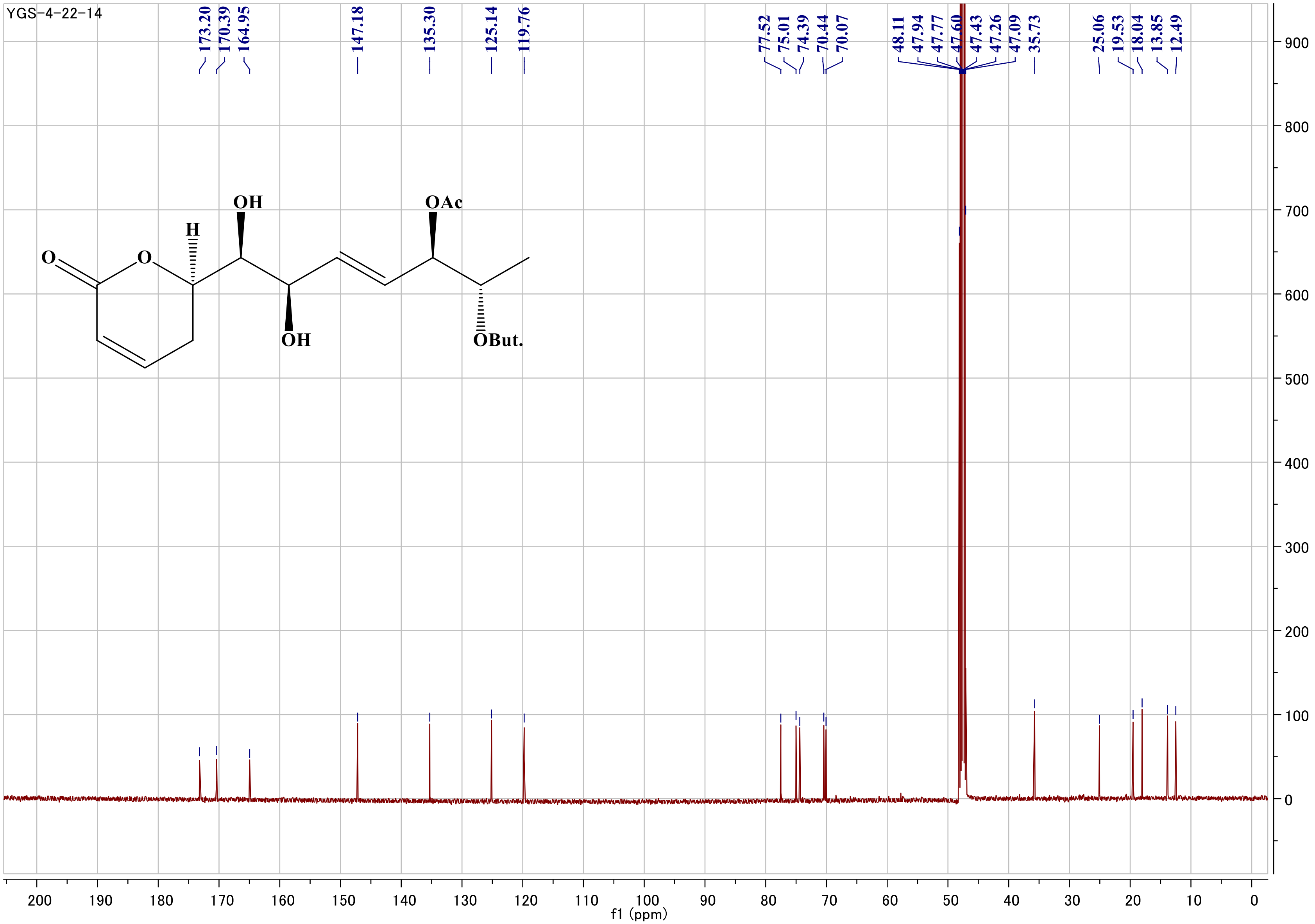

S54: ^{13}C NMR of **7**

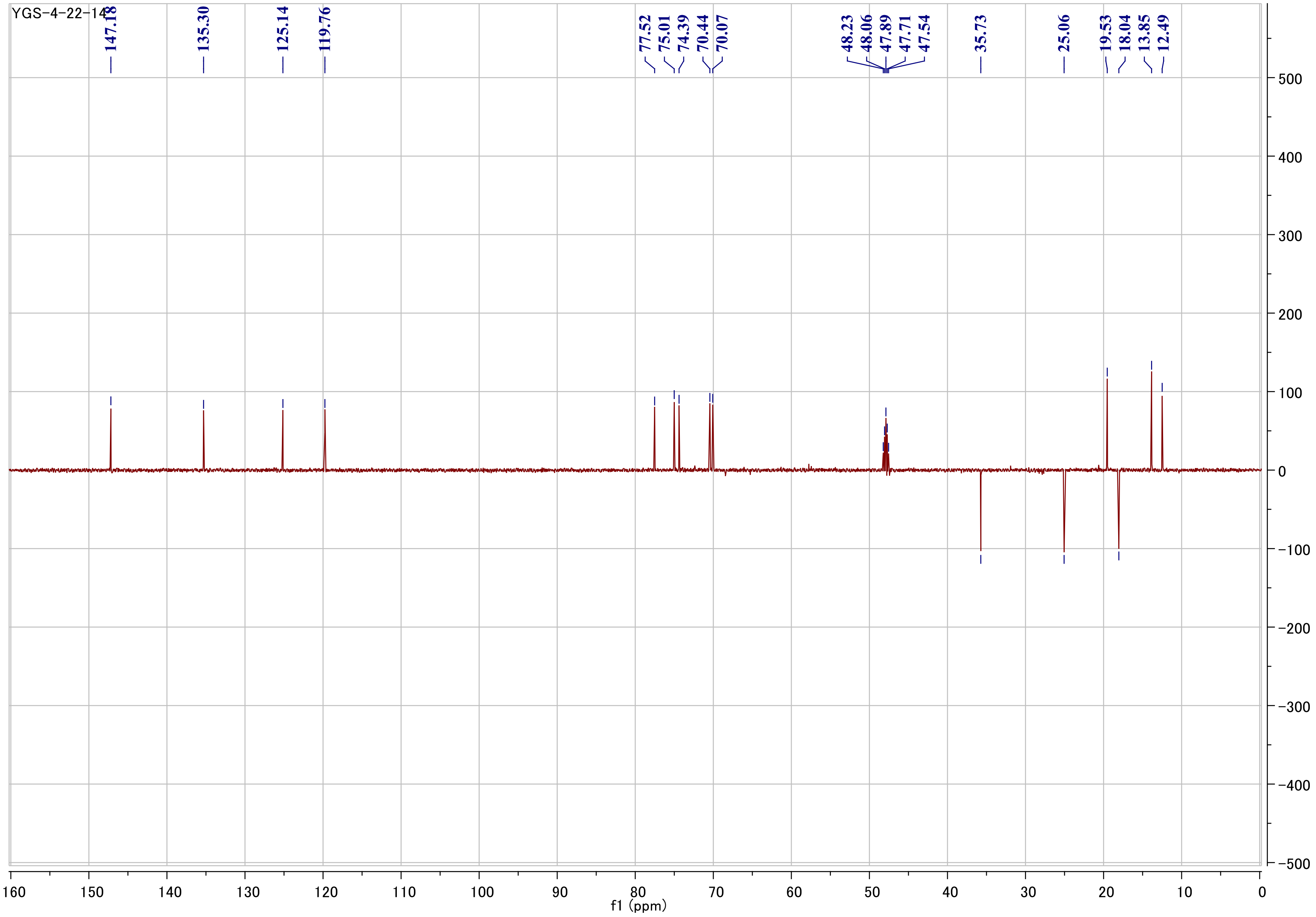

S55: DEPT-135 of **7**

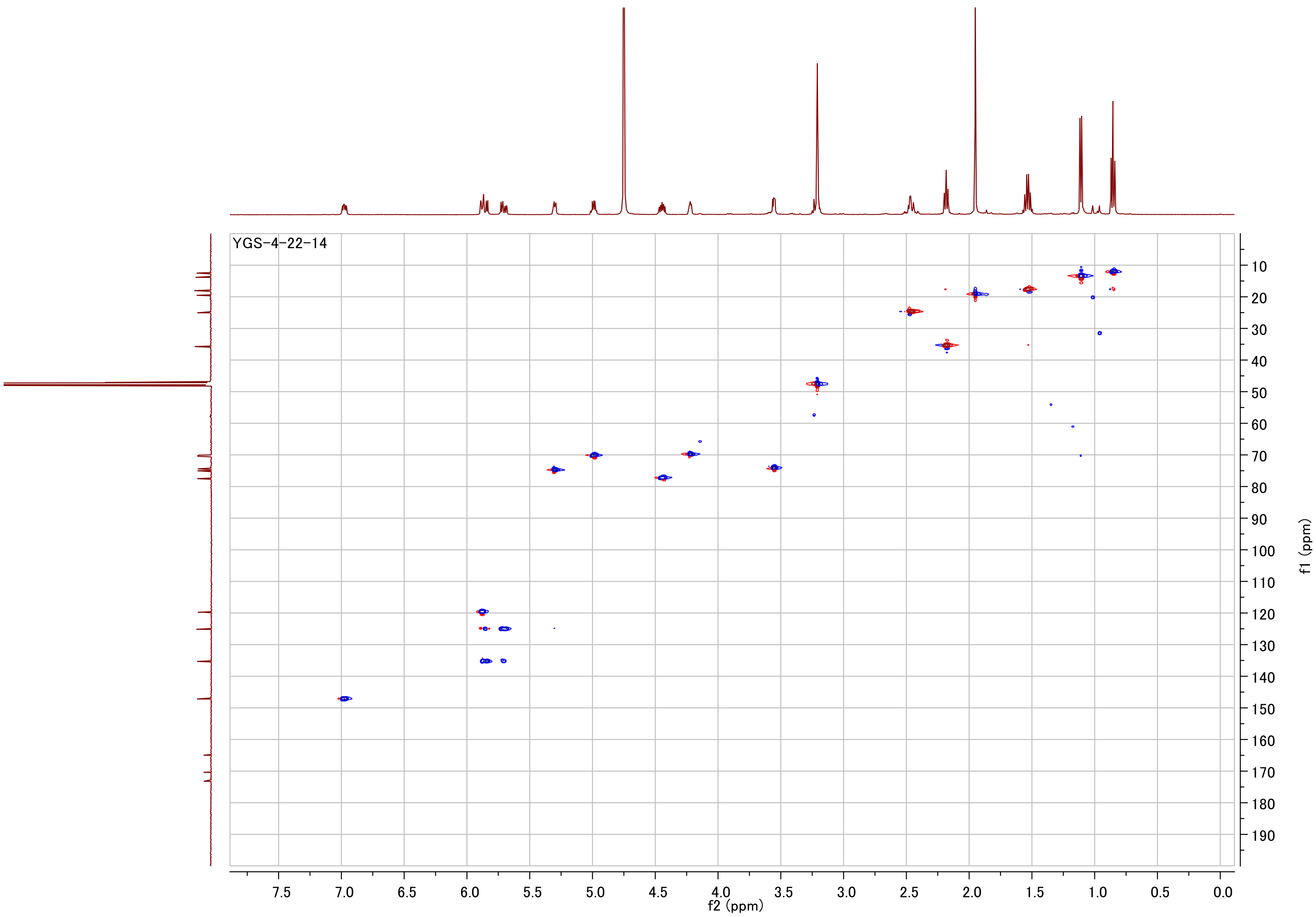
YGS-4-22-14
f2 (ppm)
f1 (ppm)
7.5
7.0
6.5
6.0
5.5
5.0
4.5
4.0
3.5
3.0
2.5
2.0
1.5
1.0
0.5
0.0
10
20
30
40
50
60
70
80
90
100
110
120
130
140
150
160
170
180
190

S56: HSQC of **7**

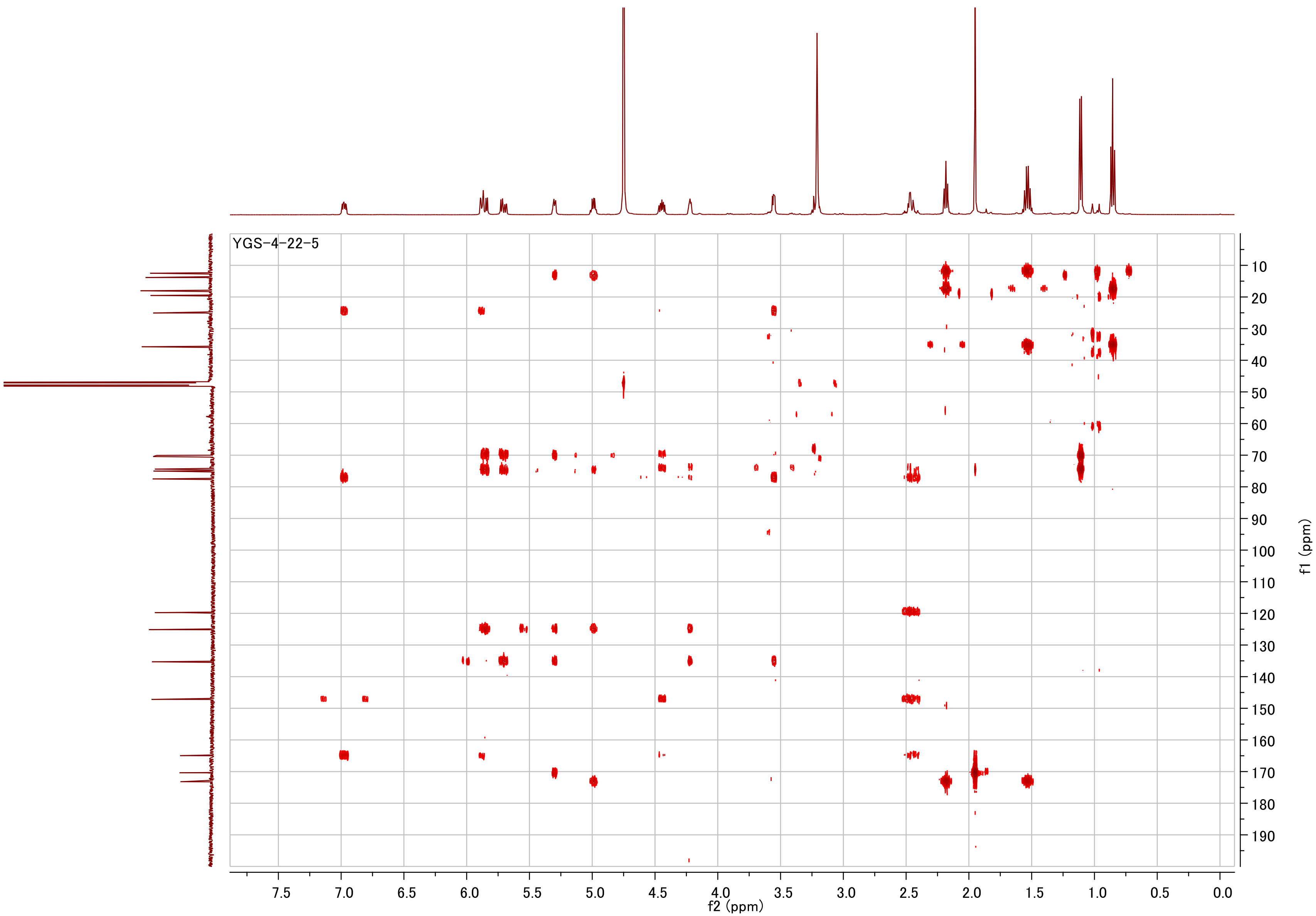
YGS-4-22-5
7.5
7.0
6.5
6.0
5.5
5.0
4.5
4.0
3.5
3.0
2.5
2.0
1.5
1.0
0.5
0.0
f2 (ppm)
10
20
30
40
50
60
70
80
90
100
110
120
130
140
150
160
170
180
190
f1 (ppm)

S57: HMBC of **7**

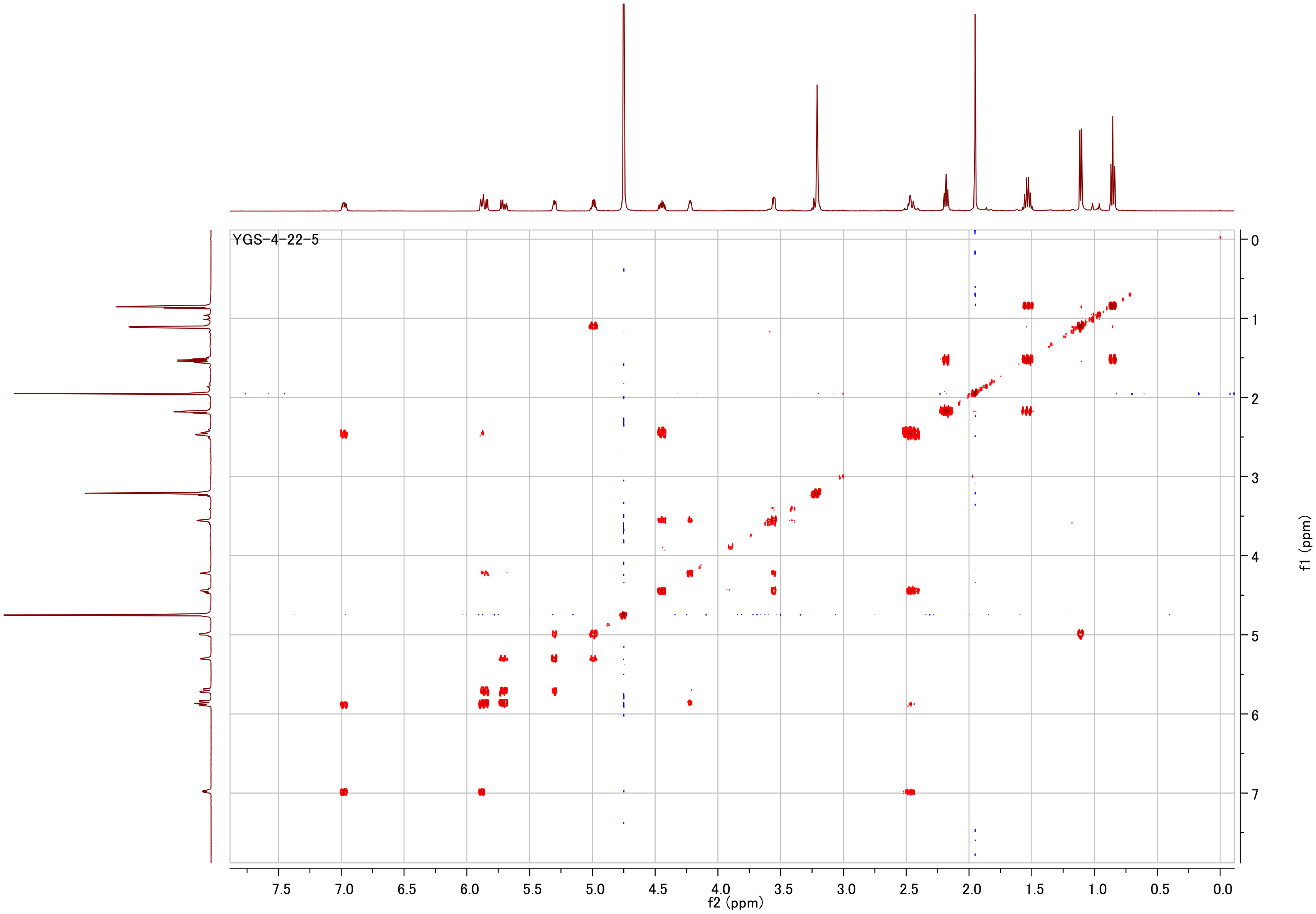

S58: ^{1}H ^{1}H COSY of **7**

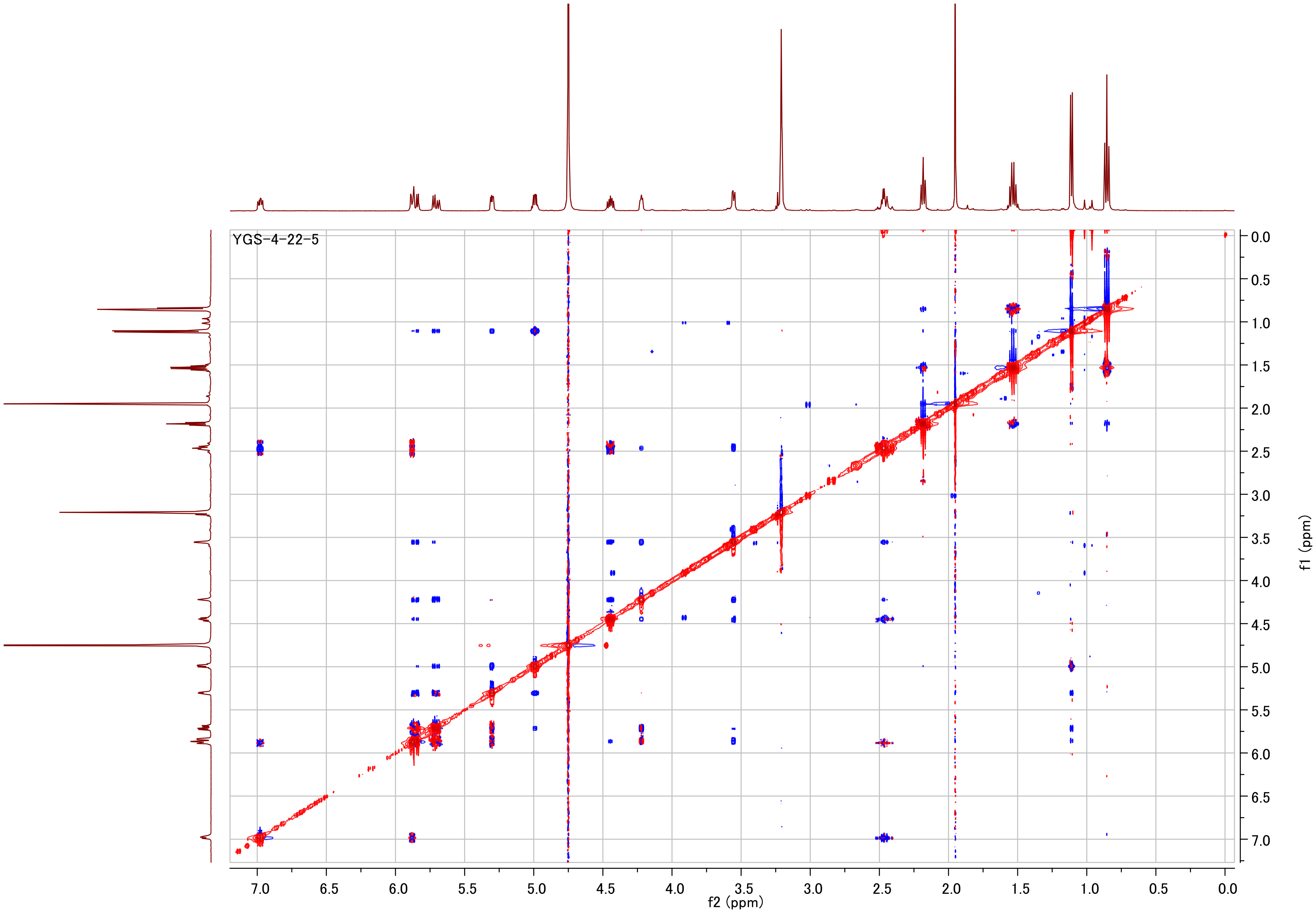

S59: NOESY of **7**

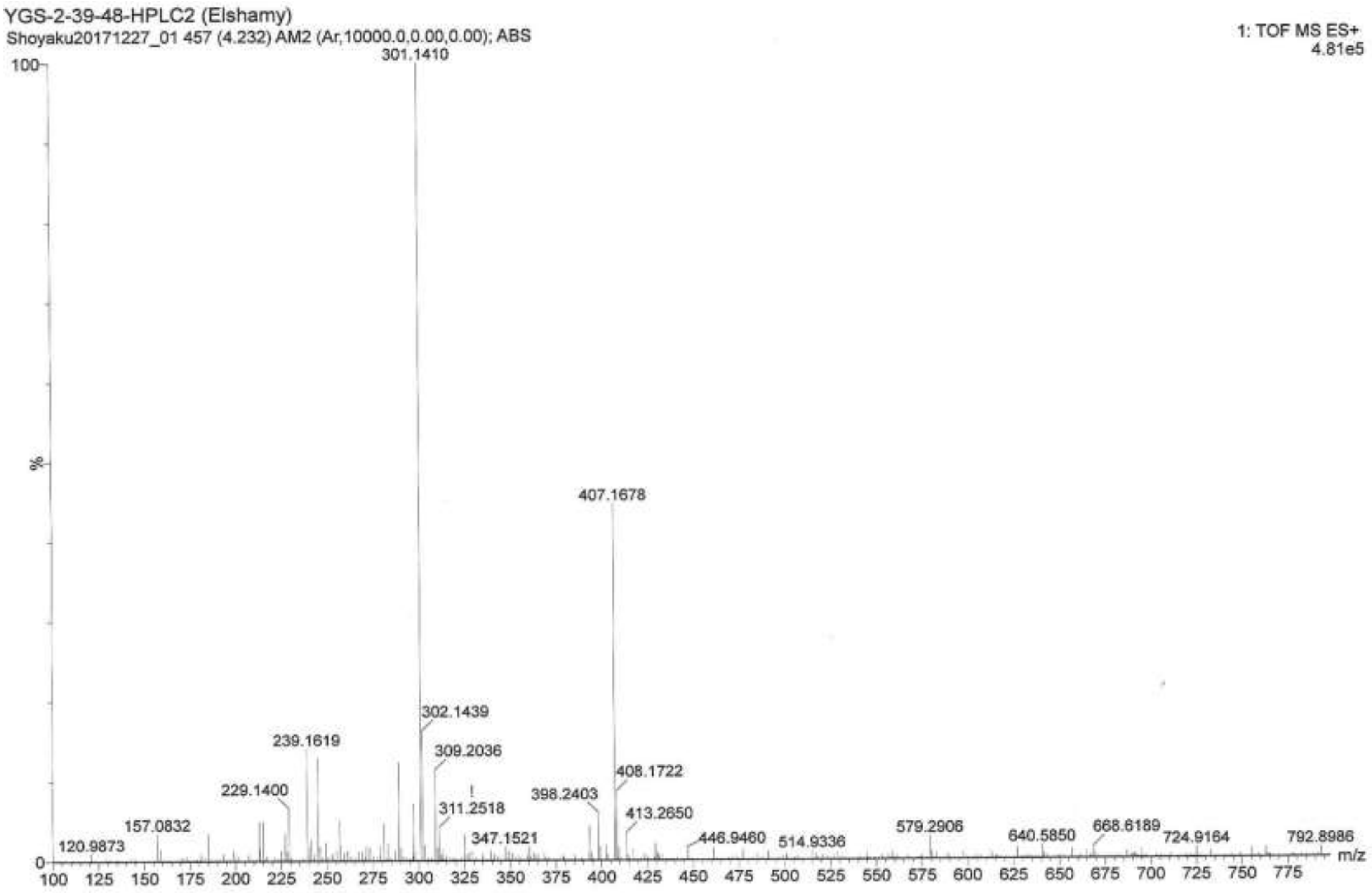

S60: TOFESIMS of **8**

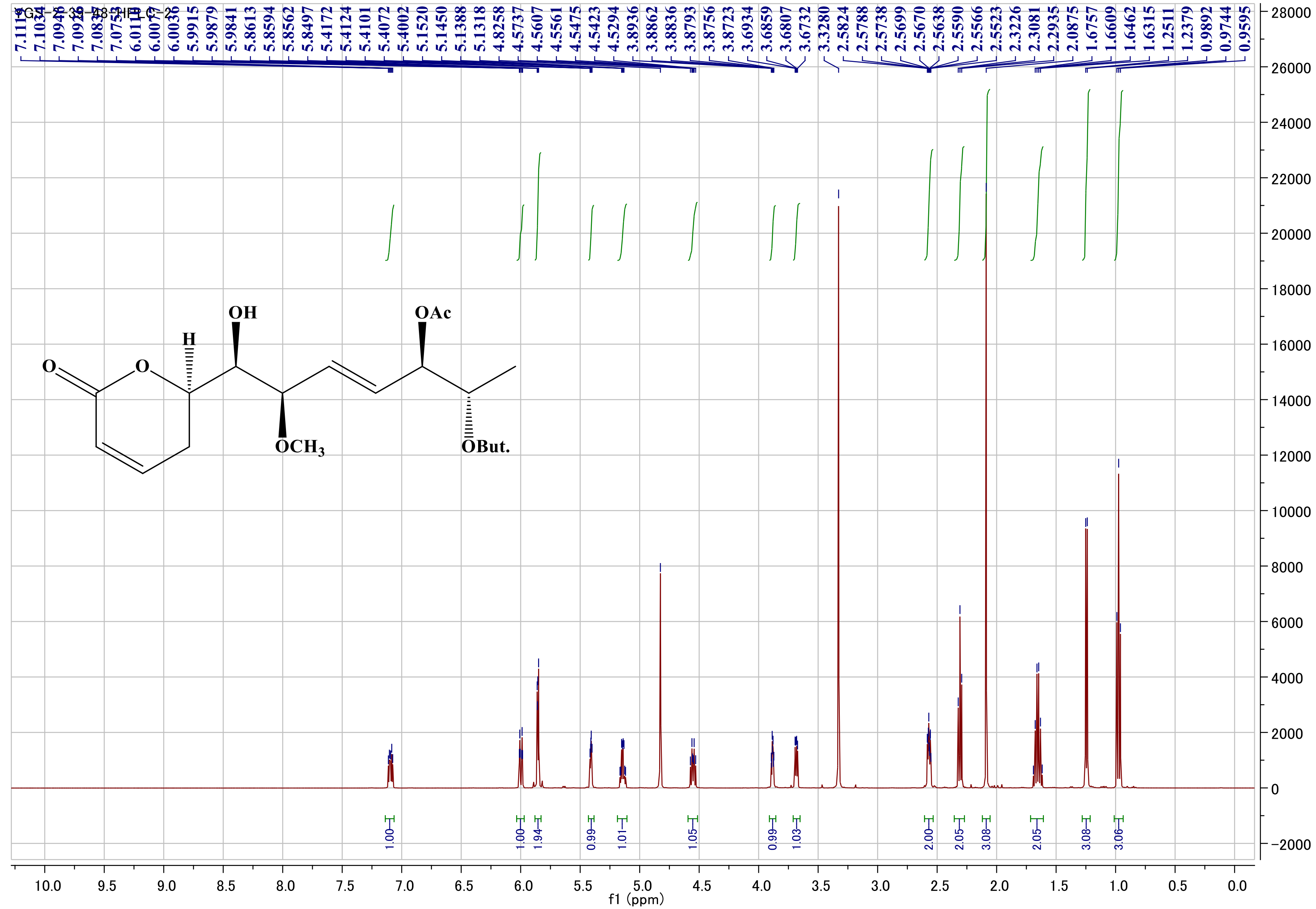

S61: ^{1}H NMR of **8**

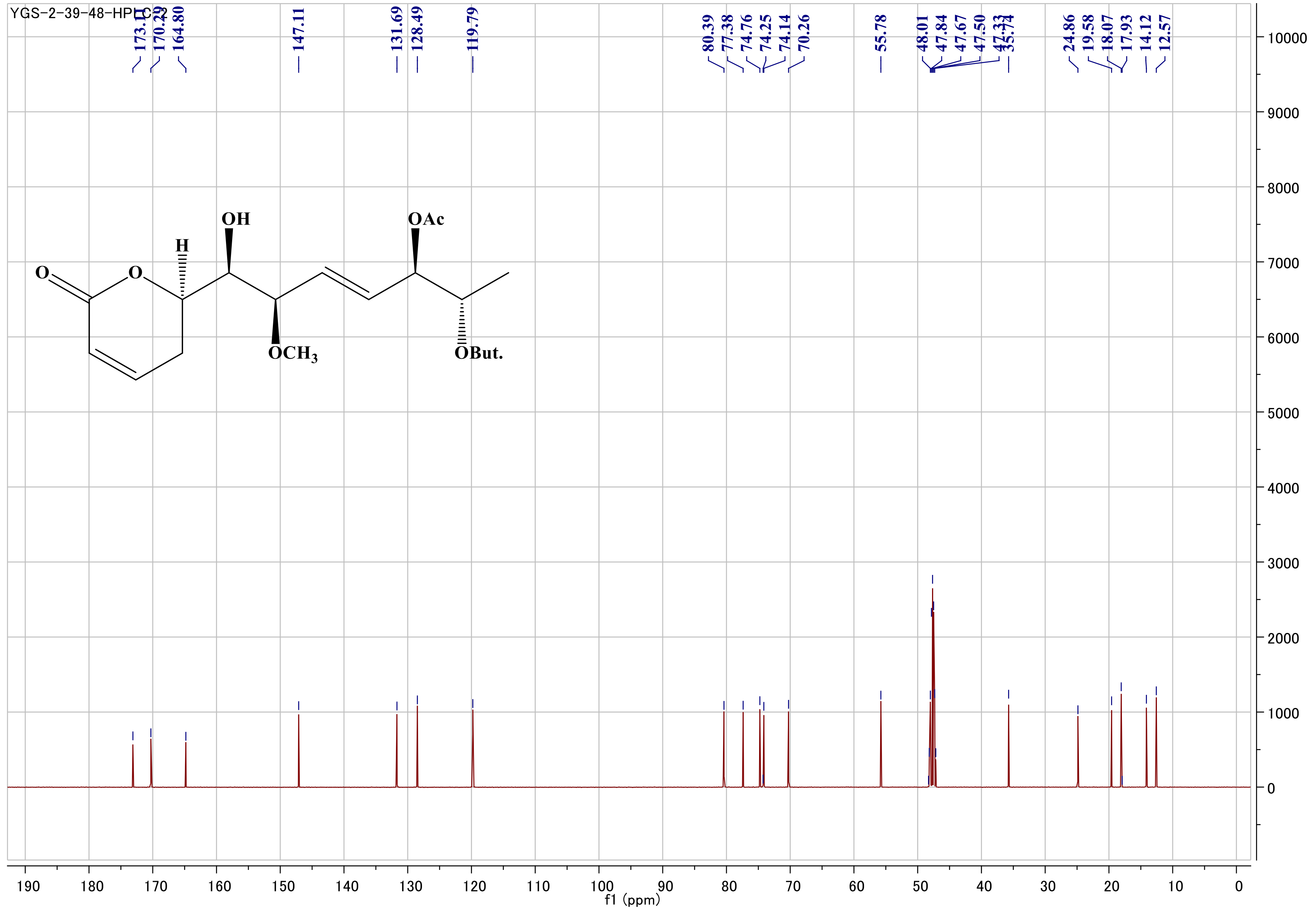

S62: ^{13}C NMR of **8**

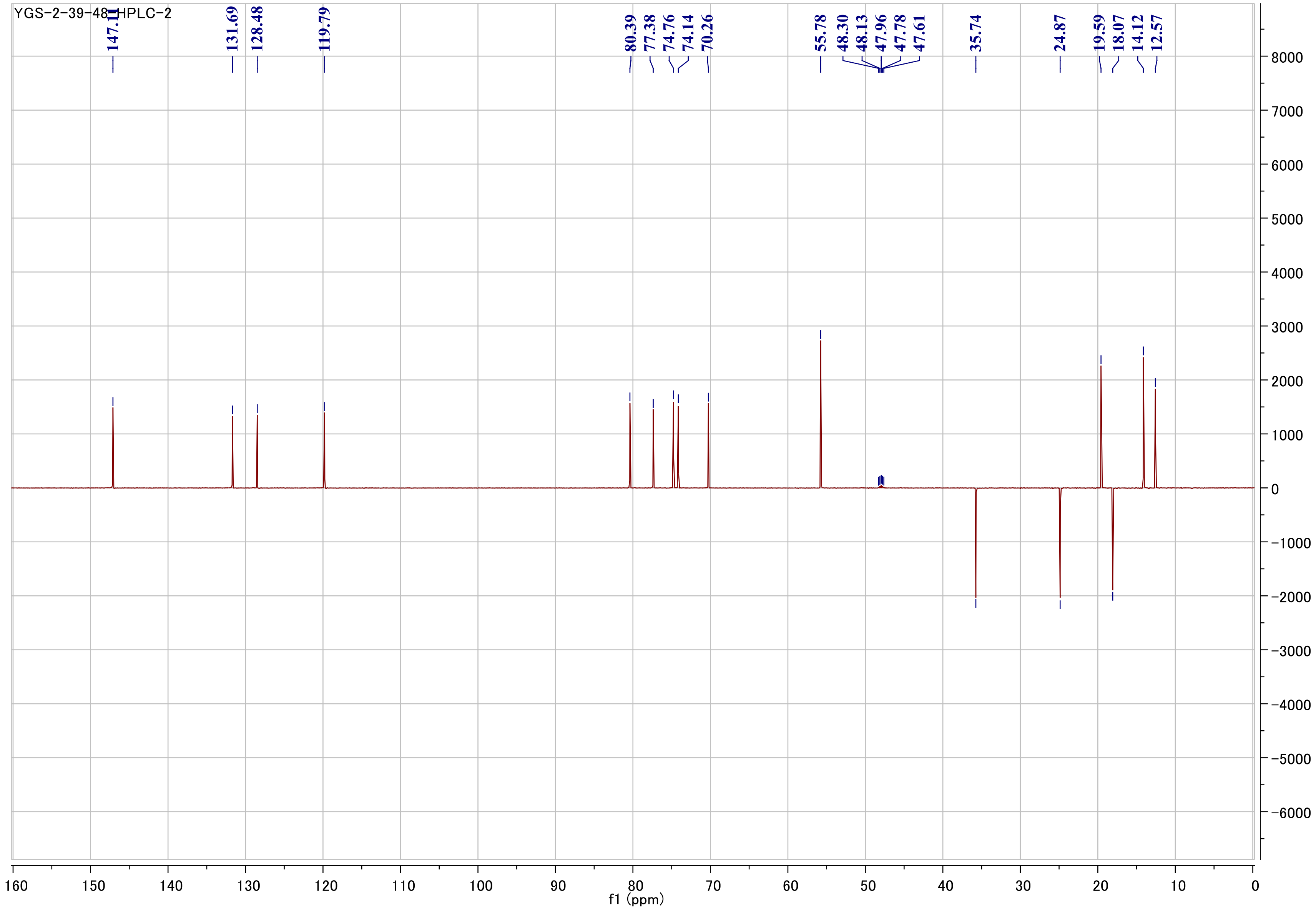

S63: DEPT-135 of **8**

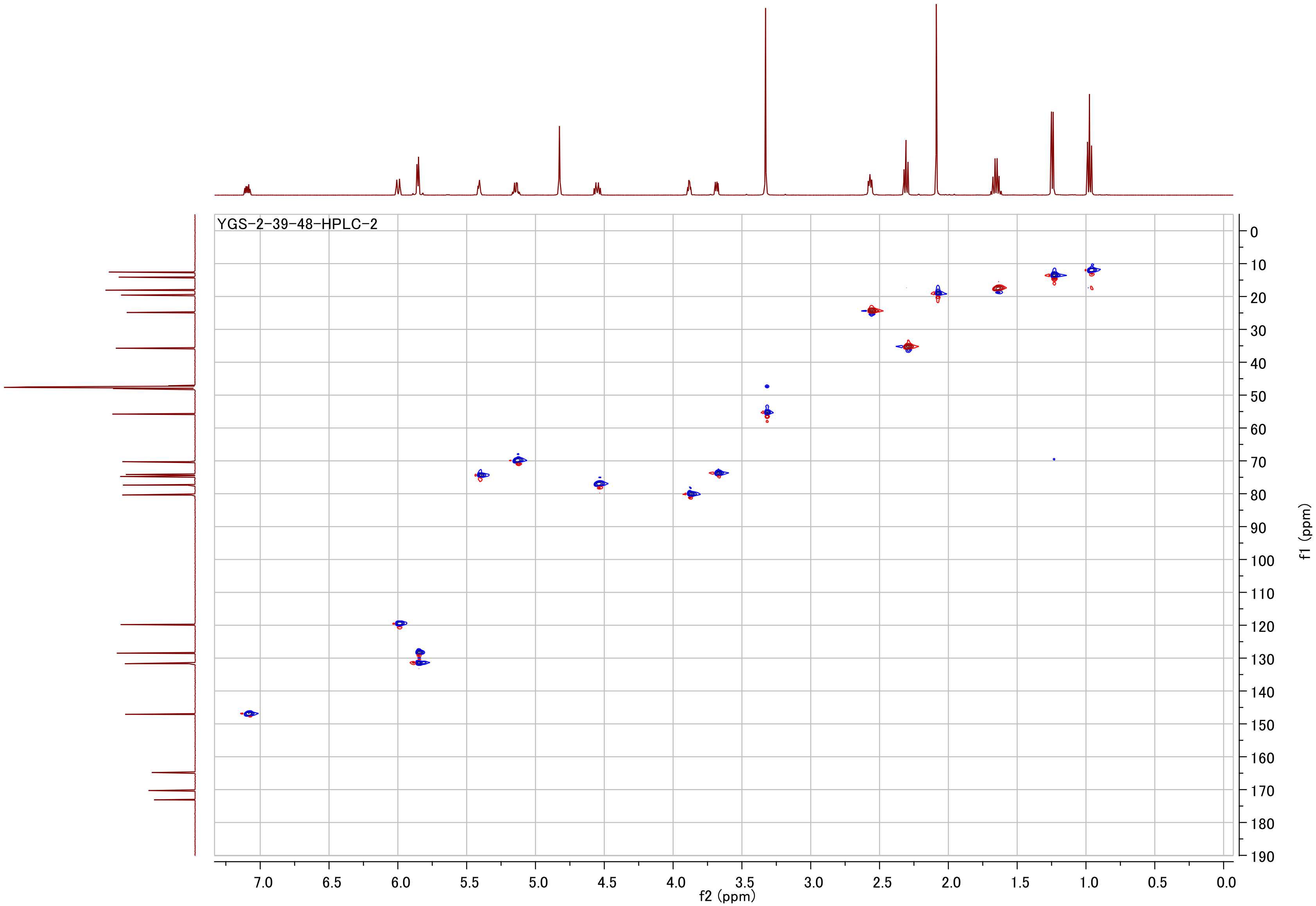

S64: HSQC of **8**

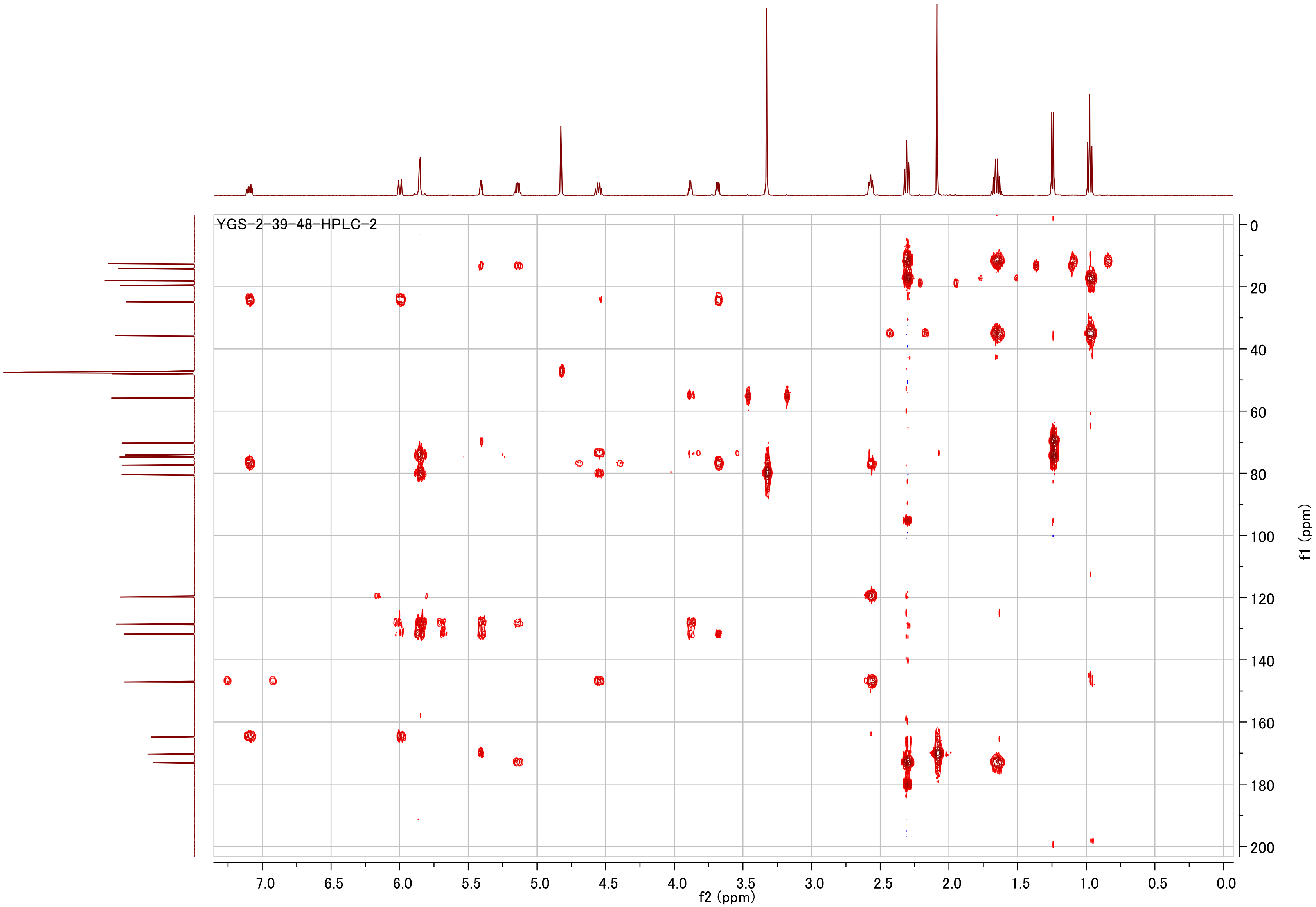

S65: HMBC of **8**

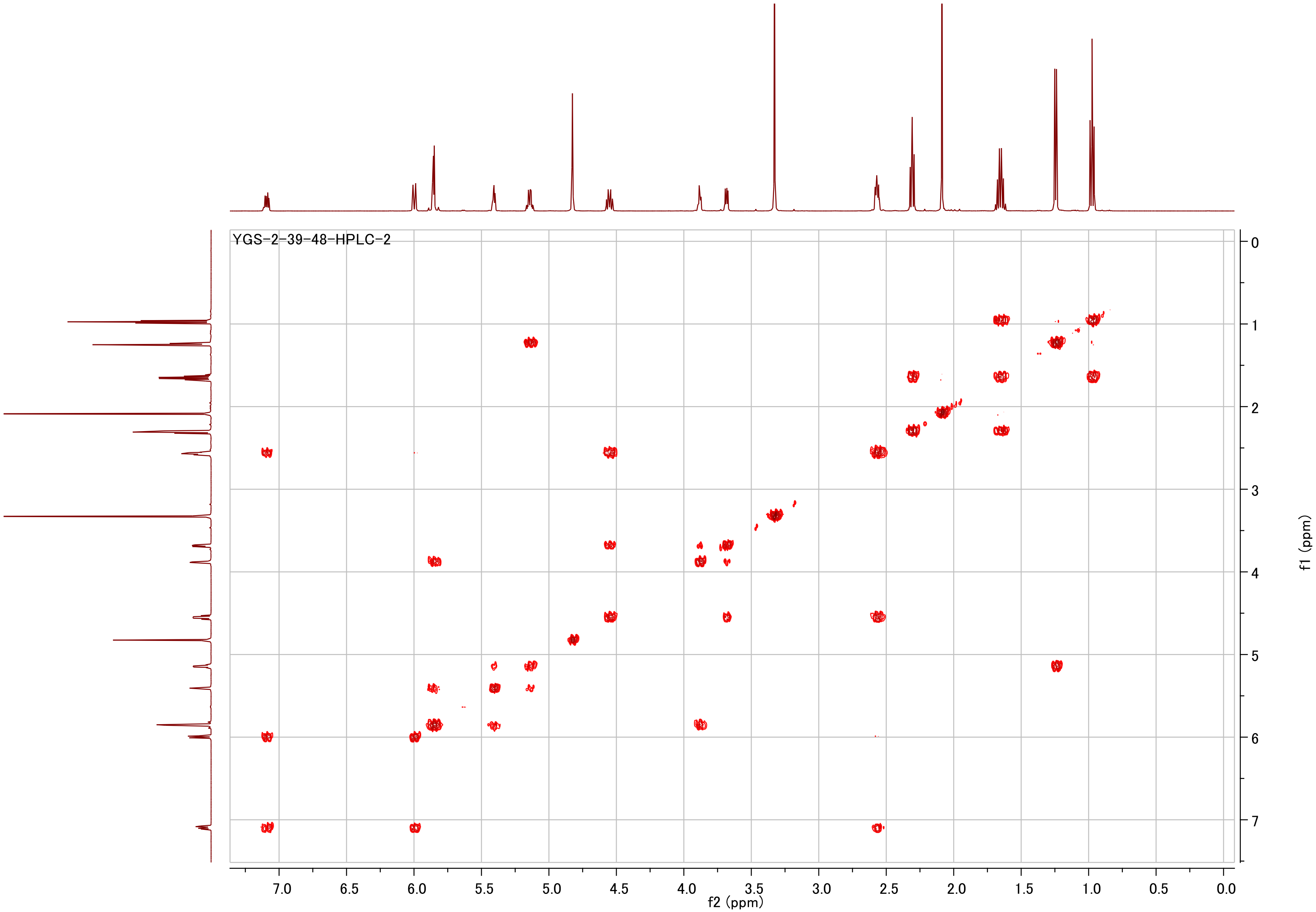

S66: ^{1}H ^{1}H COSY of **8**

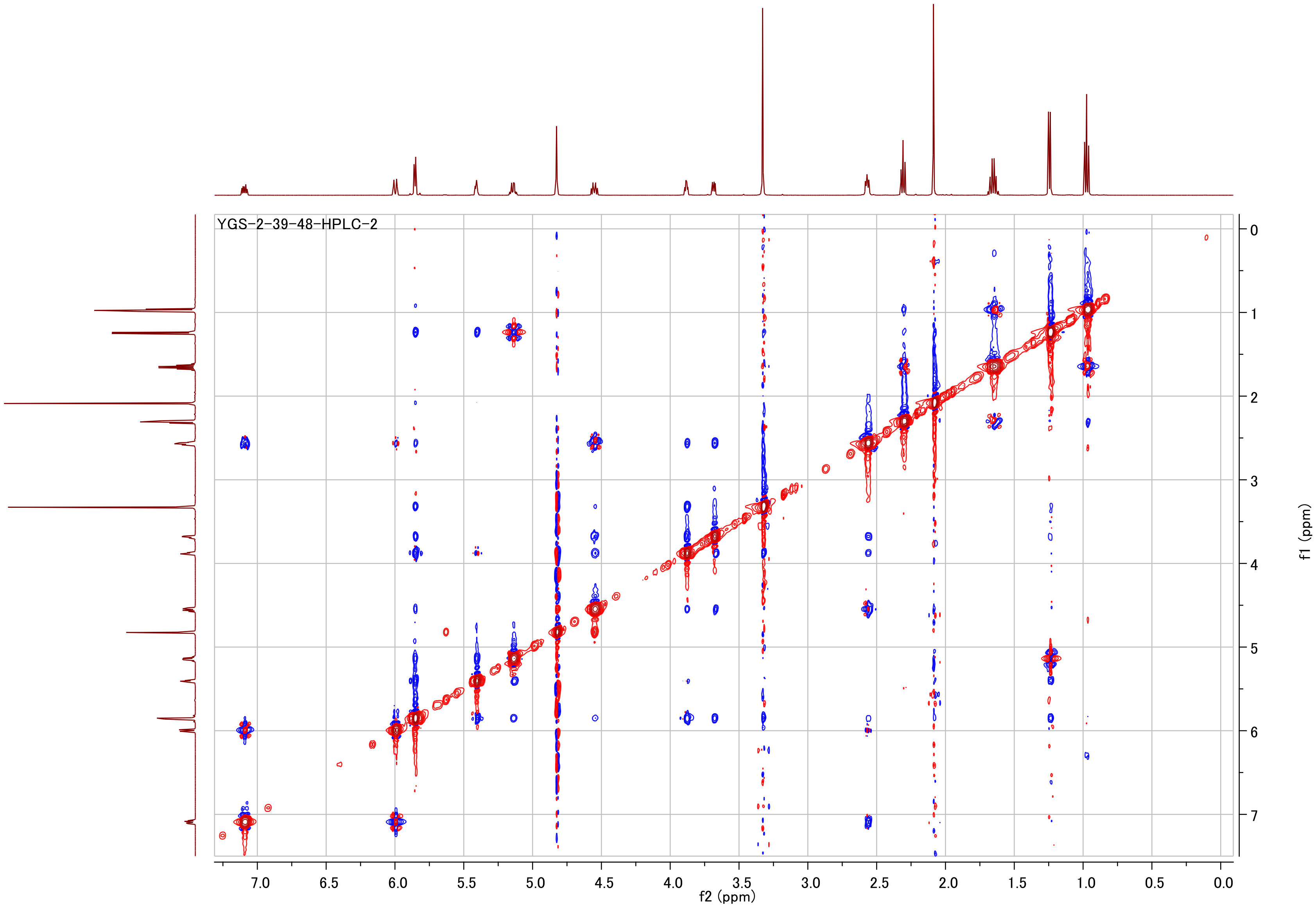

S67: NOESY of **8**

[Mass Spectrum]
Data : Umeyama-CI.05-Mar-2018.003 Date : 05-Mar-2018 15:59
Sample : YGS-4-22-7A(CH4)
Note : MStation
Inlet : Direct Ion Mode : CI+
Spectrum Type : Normal Ion [MF-Linear]
RT : 0.93 min Scan# : 35-k(12)[k=1.0]
BP : m/z 189 Int. : 398.03 (4173664)
Output m/z range : 35 to 500 Cut Level : 0.00 %

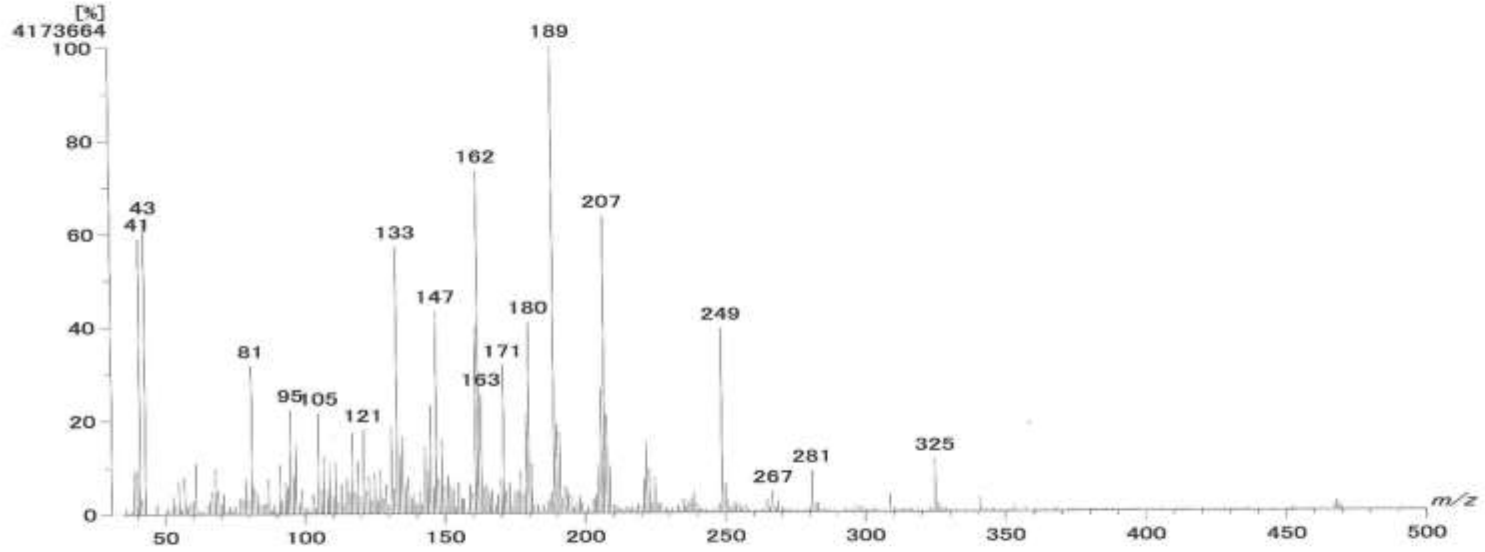

S68: LRCIMS of **9**

Data : Umeyama-CIHR.05-Mar-2018.001 Date : 05-Mar-2018 16:12
Instrument : MStation
Sample : YGS-4-22-7A
Note : MStation
Inlet : Direct Ion Mode : CI+
RT : 1.22 min Scan# : 29
Elements : C 150/0, H 250/0, O 50/0
Mass Tolerance : 5mmu
Unsaturation (U.S.) : 0.0 – 15.0

	Observed m/z	Int%	Err[ppm / mmu]	U.S.	Composition
1	267.1236	12.71	+1.3 / +0.4	5.5	C14 H19 O5

S69: HRCIMS of **9**

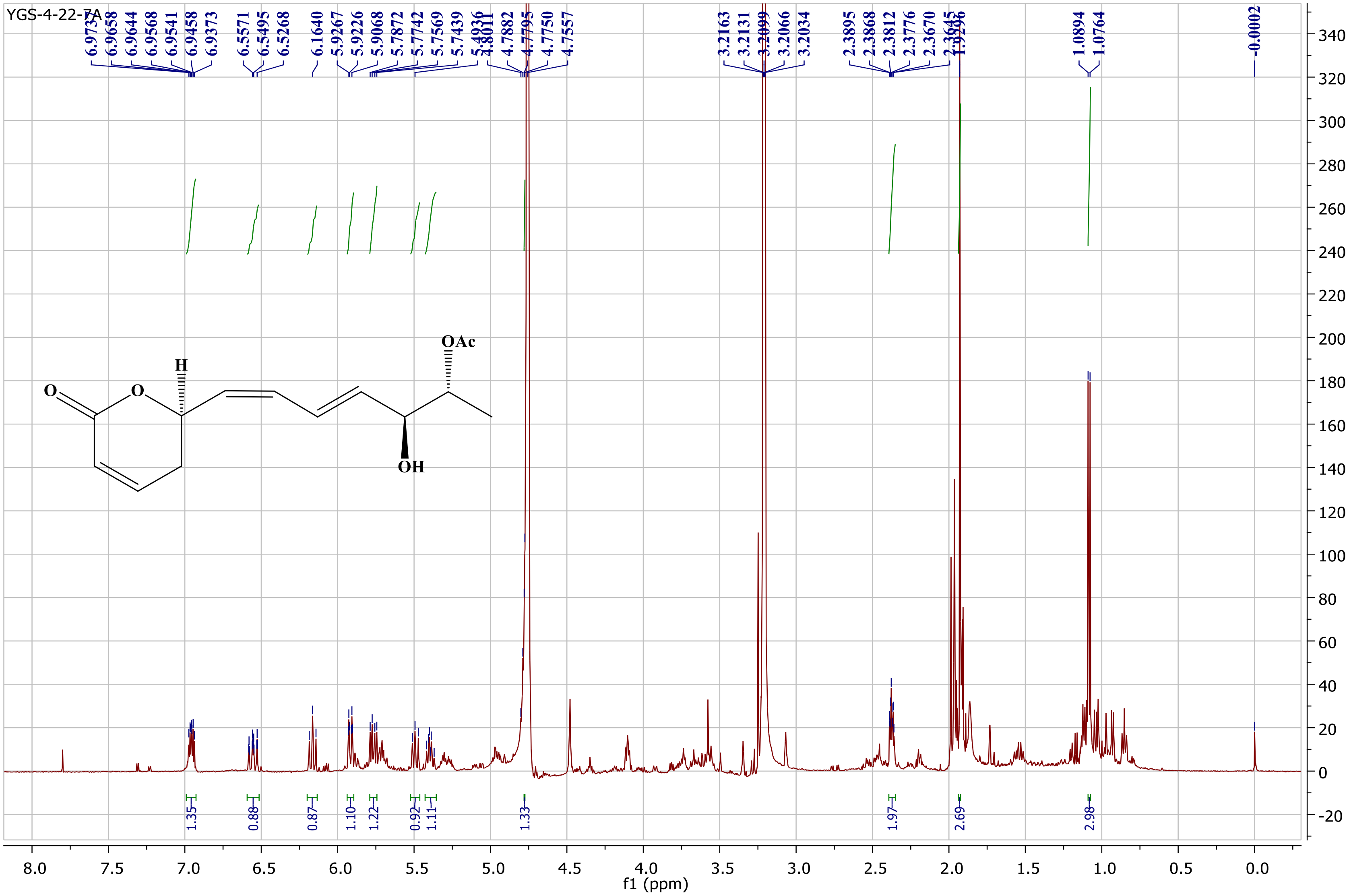

S70: ^{1}H NMR of **9**

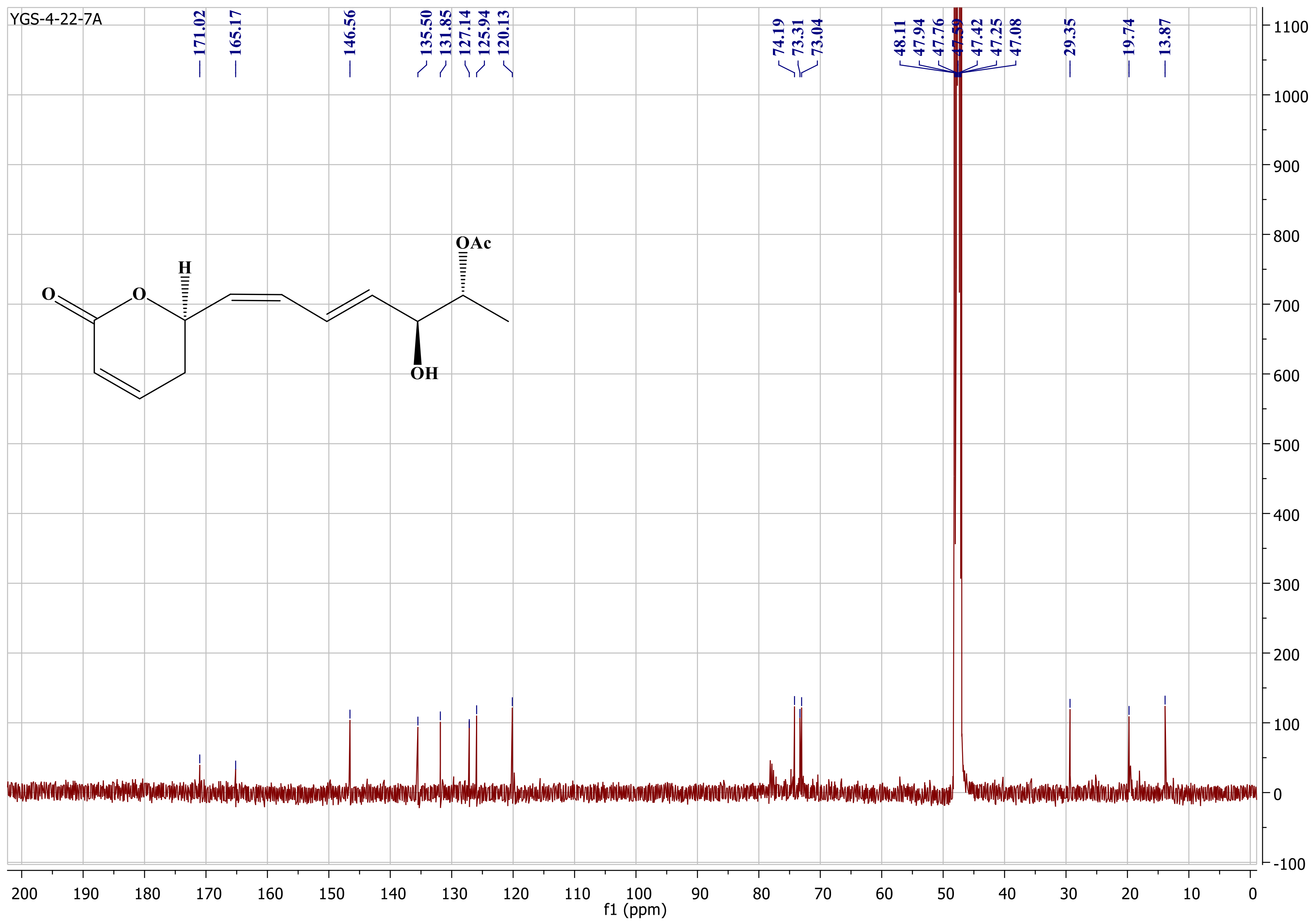

S71: ^{13}C NMR of **9**

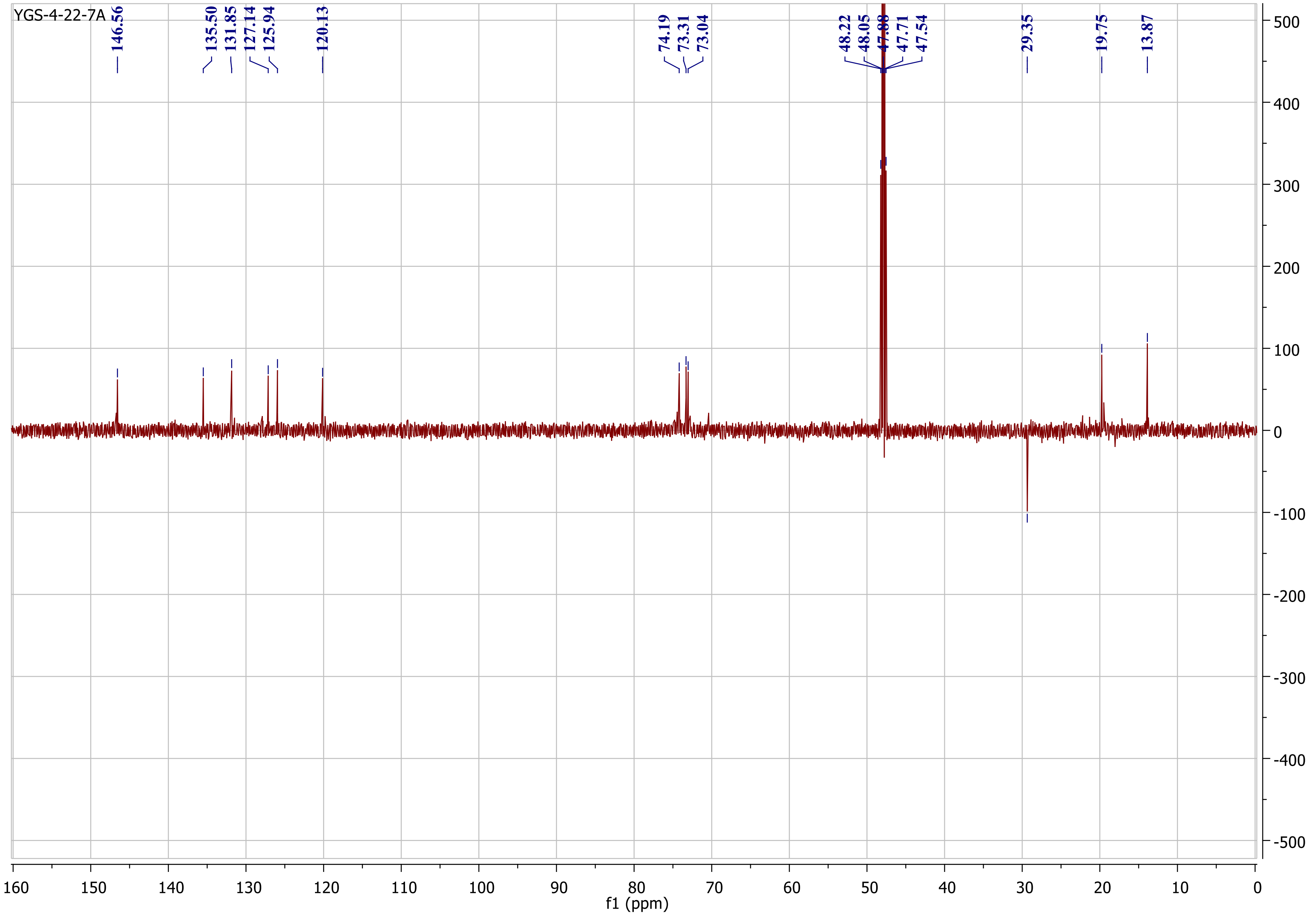

S72: DEPT-135 of **9**

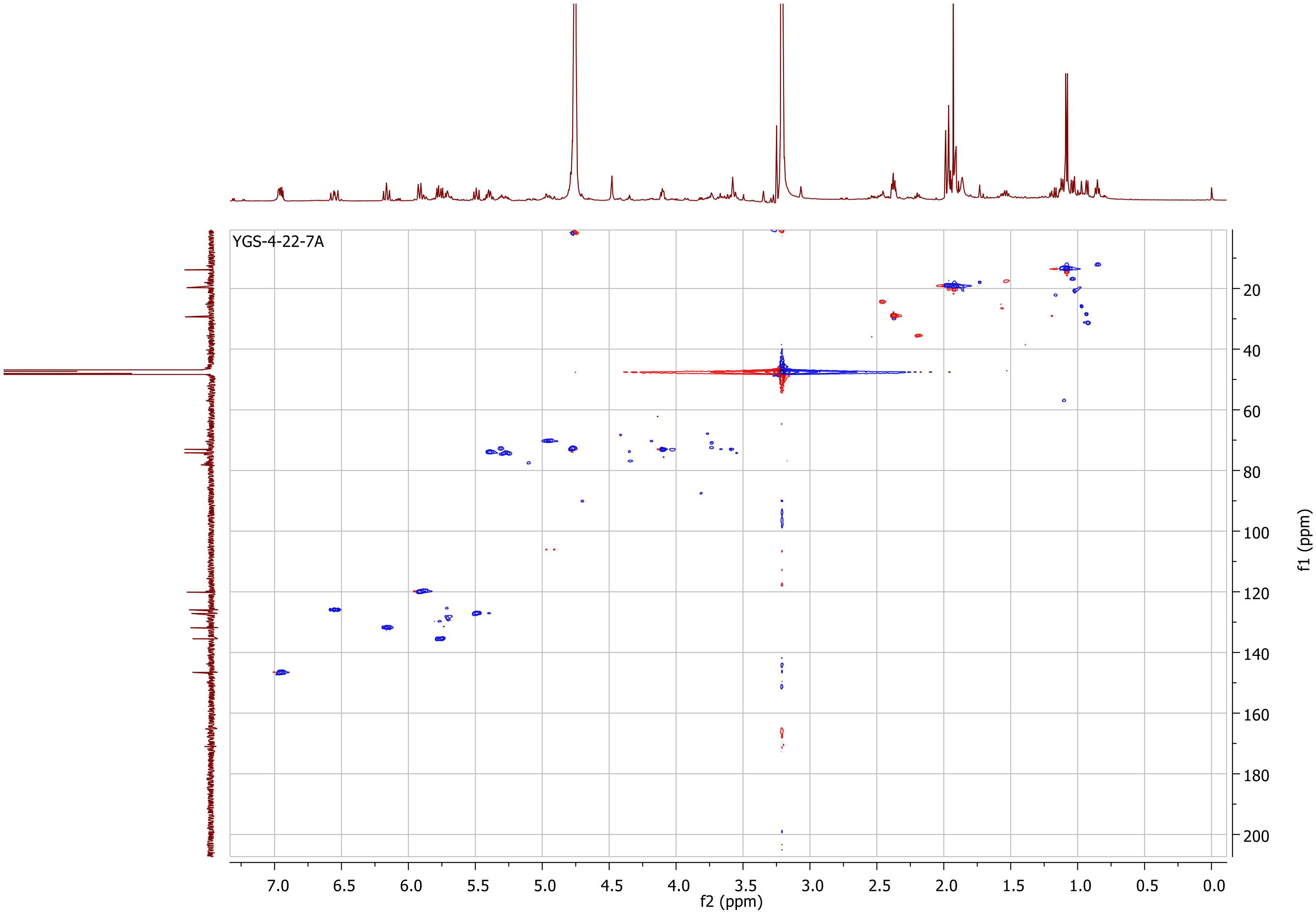
YGS-4-22-7A
f2 (ppm)
f1 (ppm)
7.0
6.5
6.0
5.5
5.0
4.5
4.0
3.5
3.0
2.5
2.0
1.5
1.0
0.5
0.0
20
40
60
80
100
120
140
160
180
200

S73: HSQC of **9**

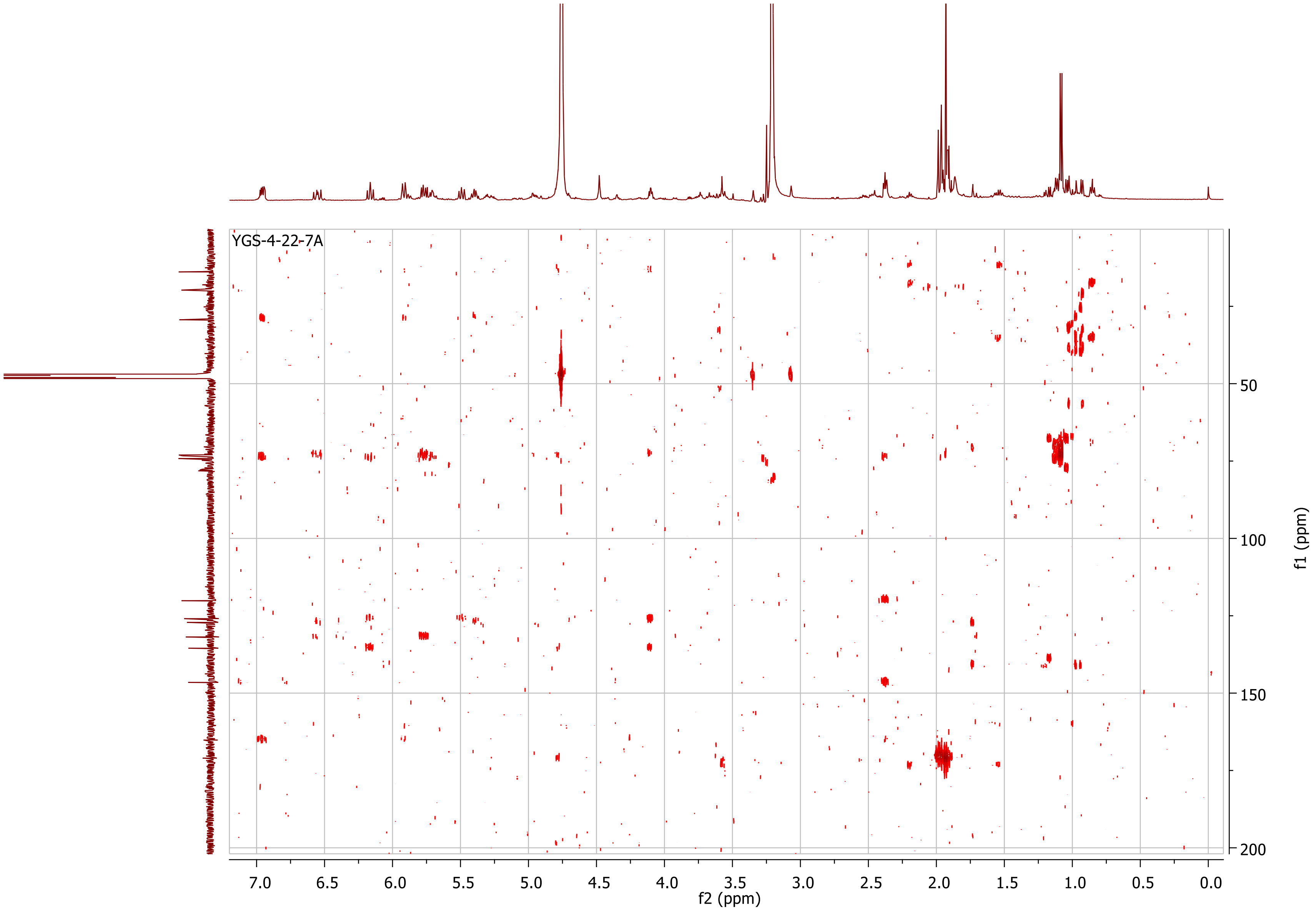
YGS-4-22-7A
7.0
6.5
6.0
5.5
5.0
4.5
4.0
3.5
3.0
2.5
2.0
1.5
1.0
0.5
0.0
f2 (ppm)
50
100
150
200
f1 (ppm)

S74: HMBC of **9**

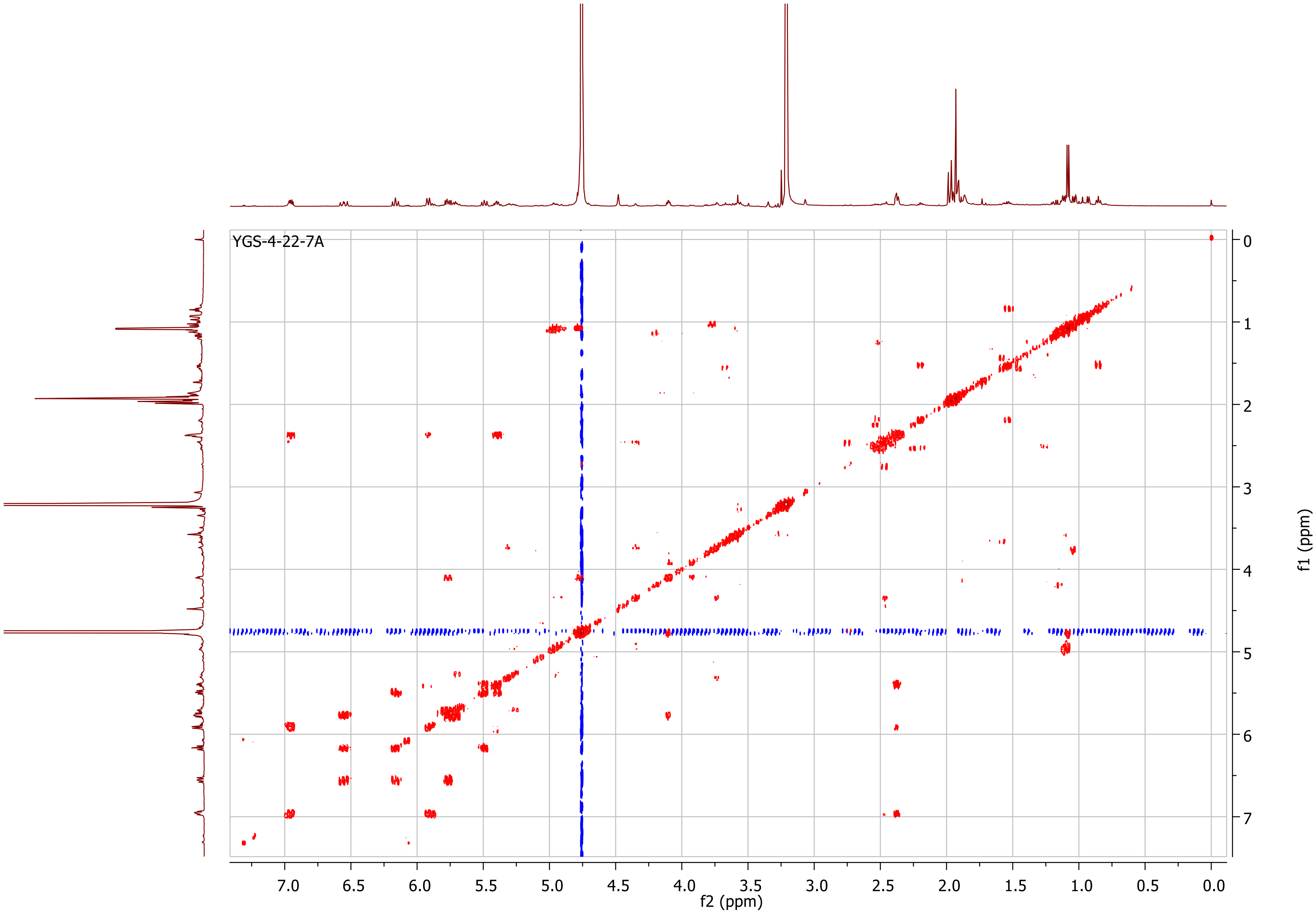

S75: ^{1}H ^{1}H COSY of **9**

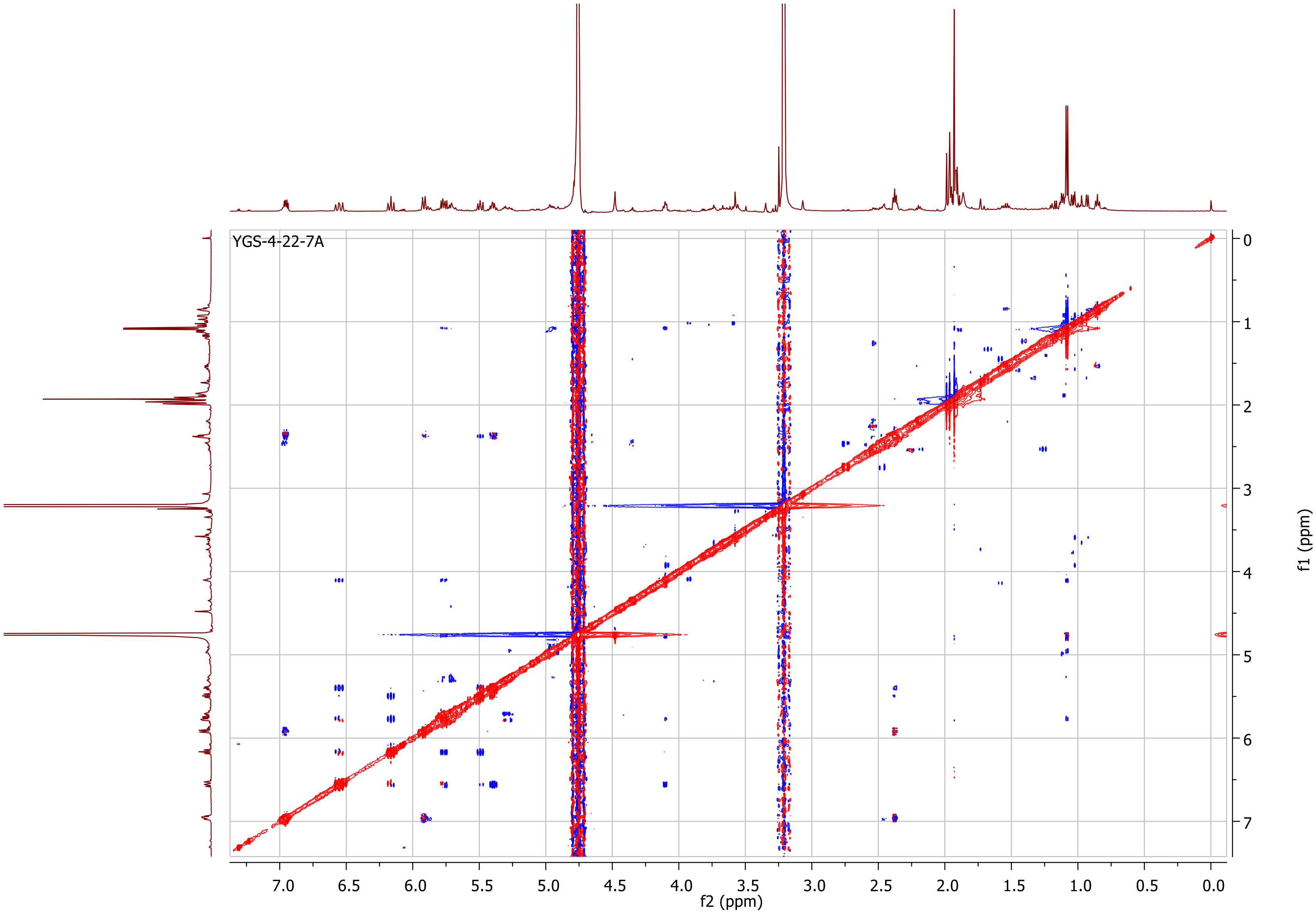

S76: NOESY of **9**

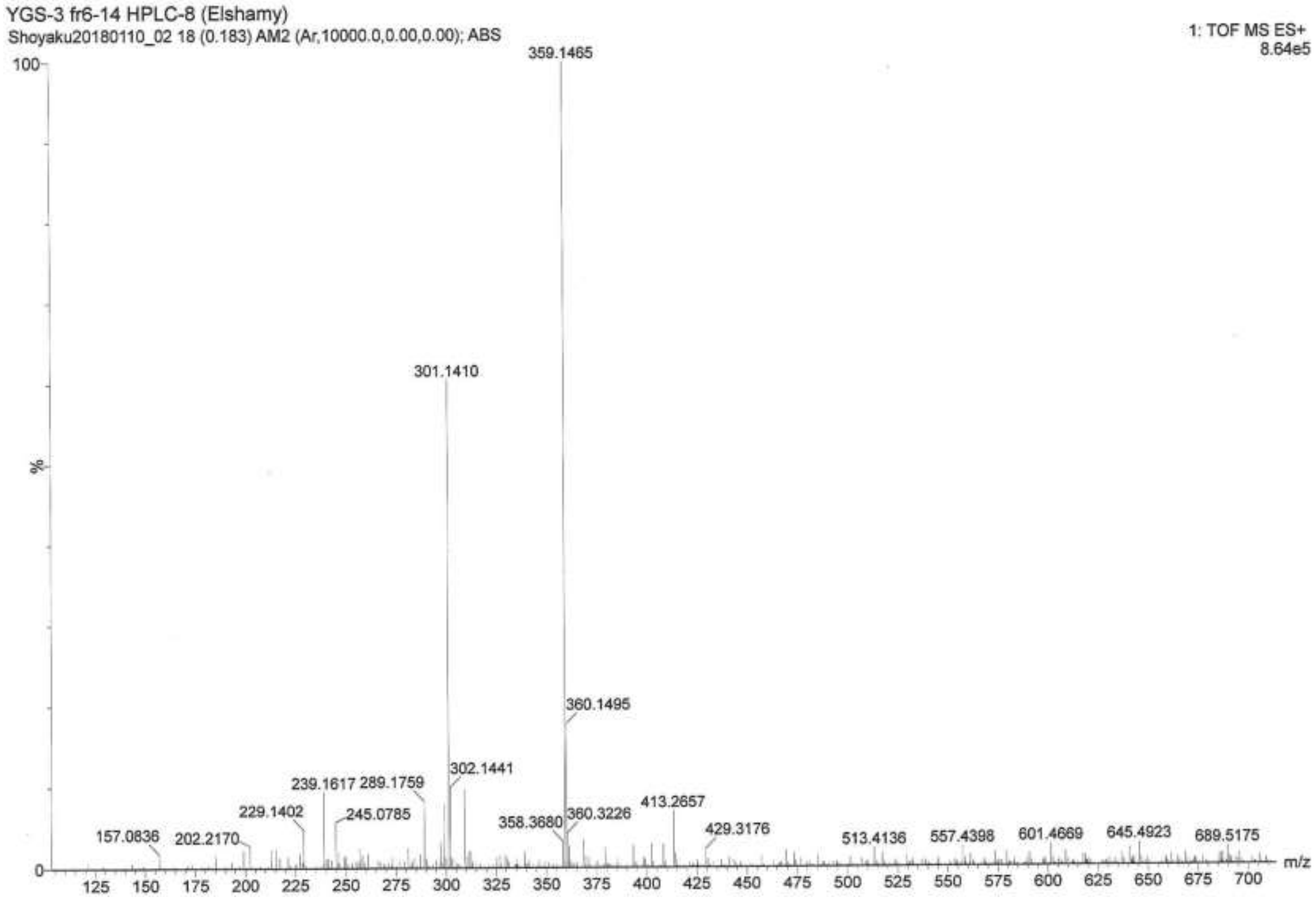

S77: TOFESIMS of **10**

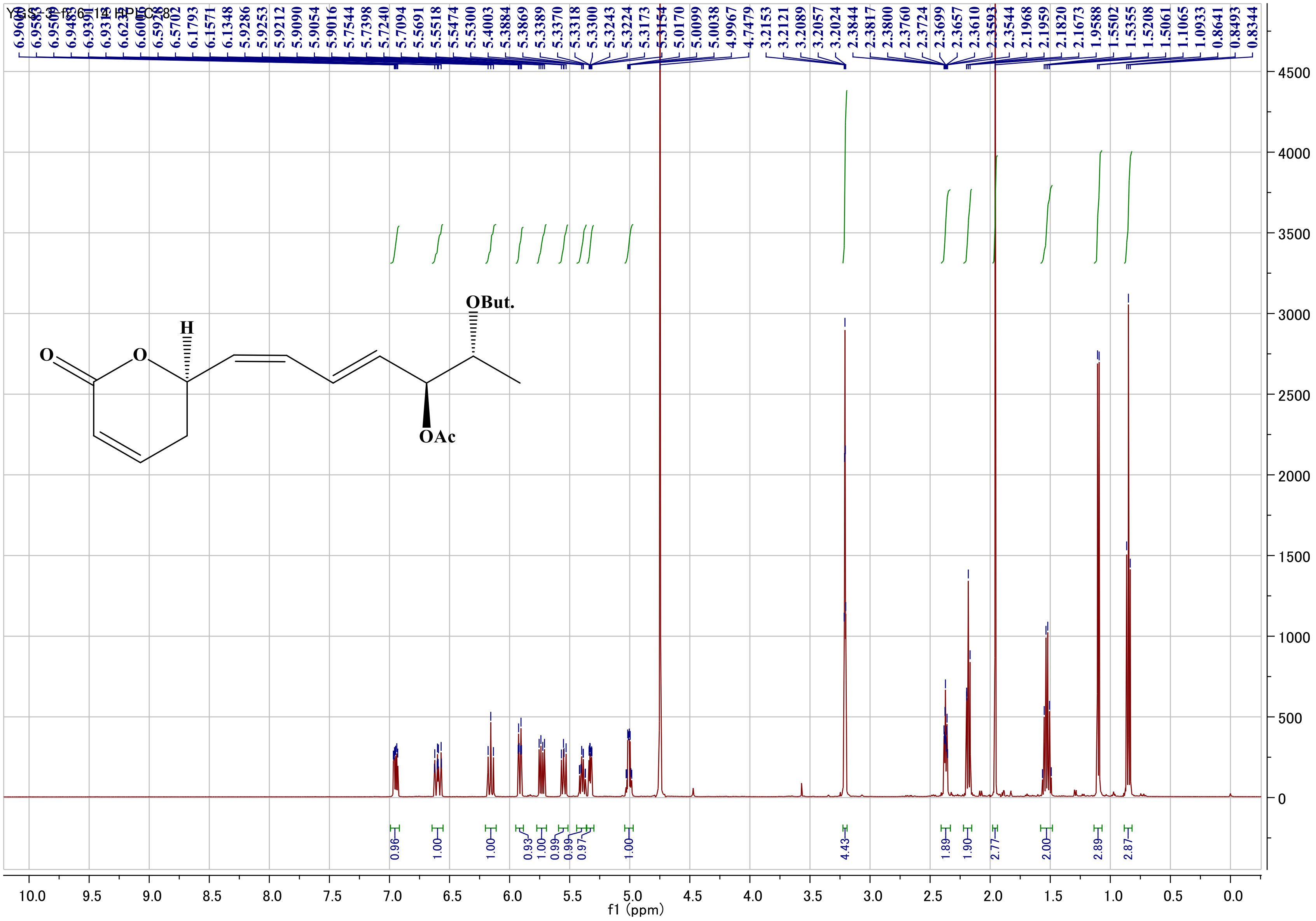

S78: ^{1}H NMR of **10**

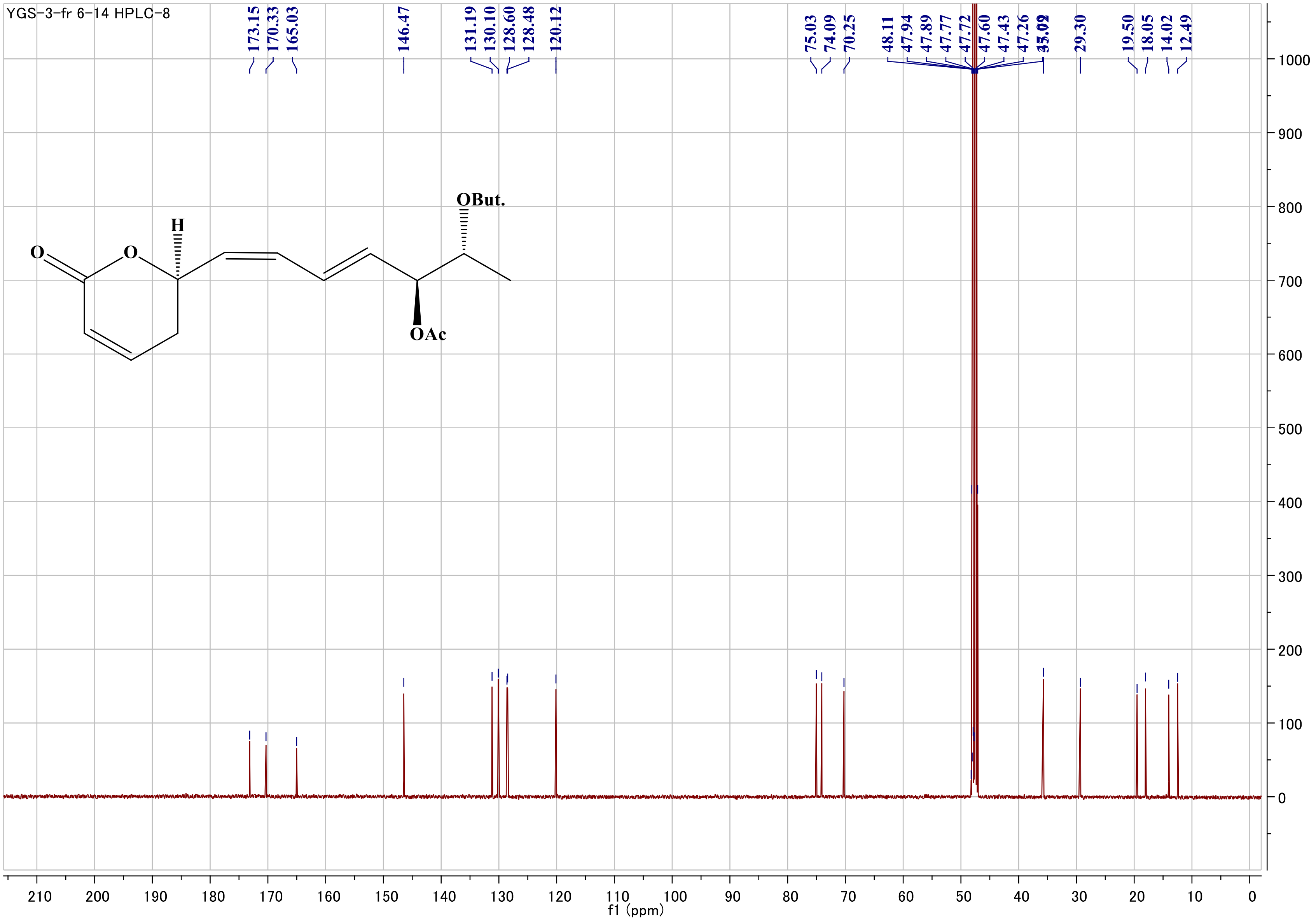

S79: ^{13}C NMR of **10**

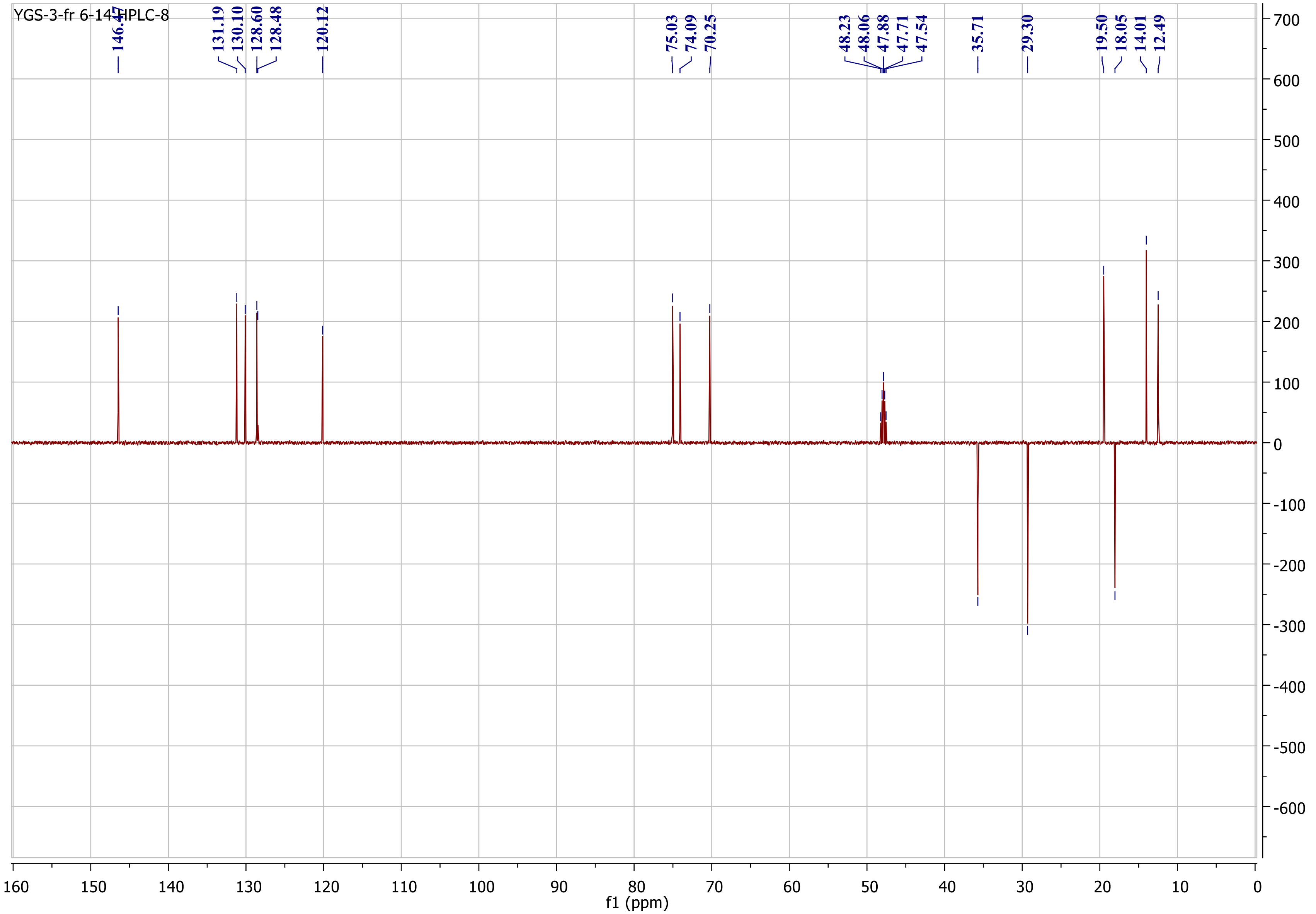

S80: DEPT-135 of **10**

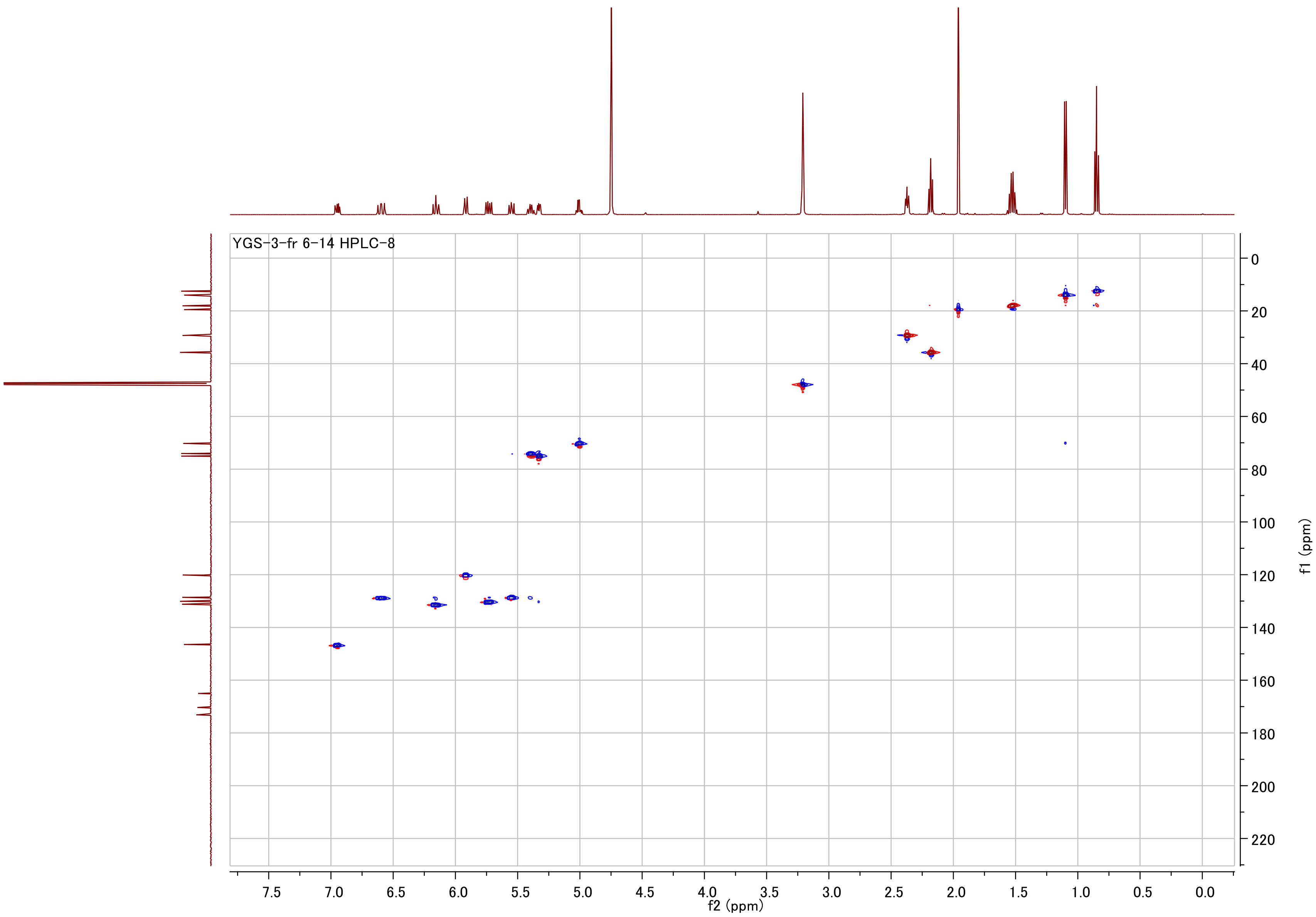

S81: HSQC of **10**

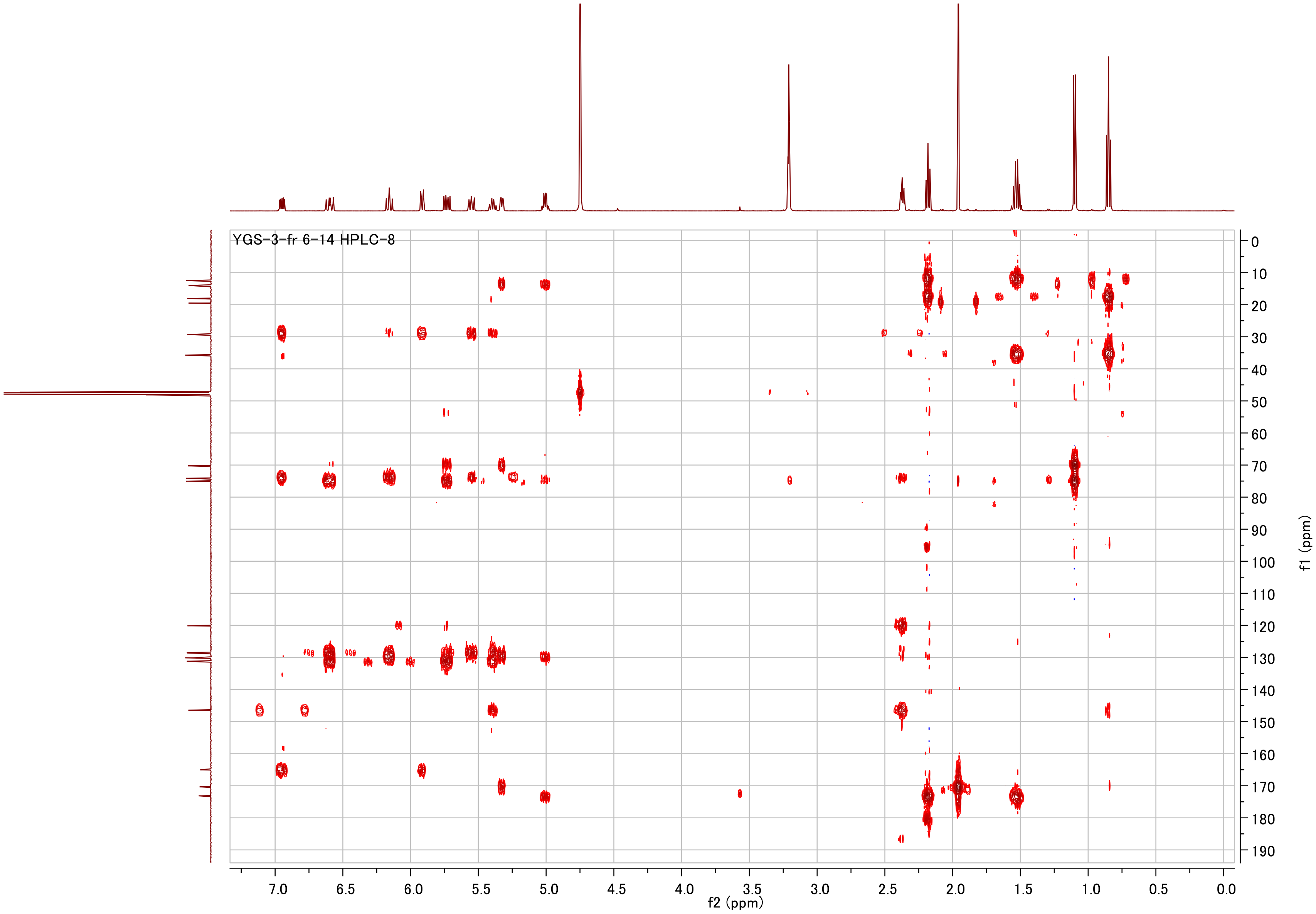

S82: HMBC of **10**

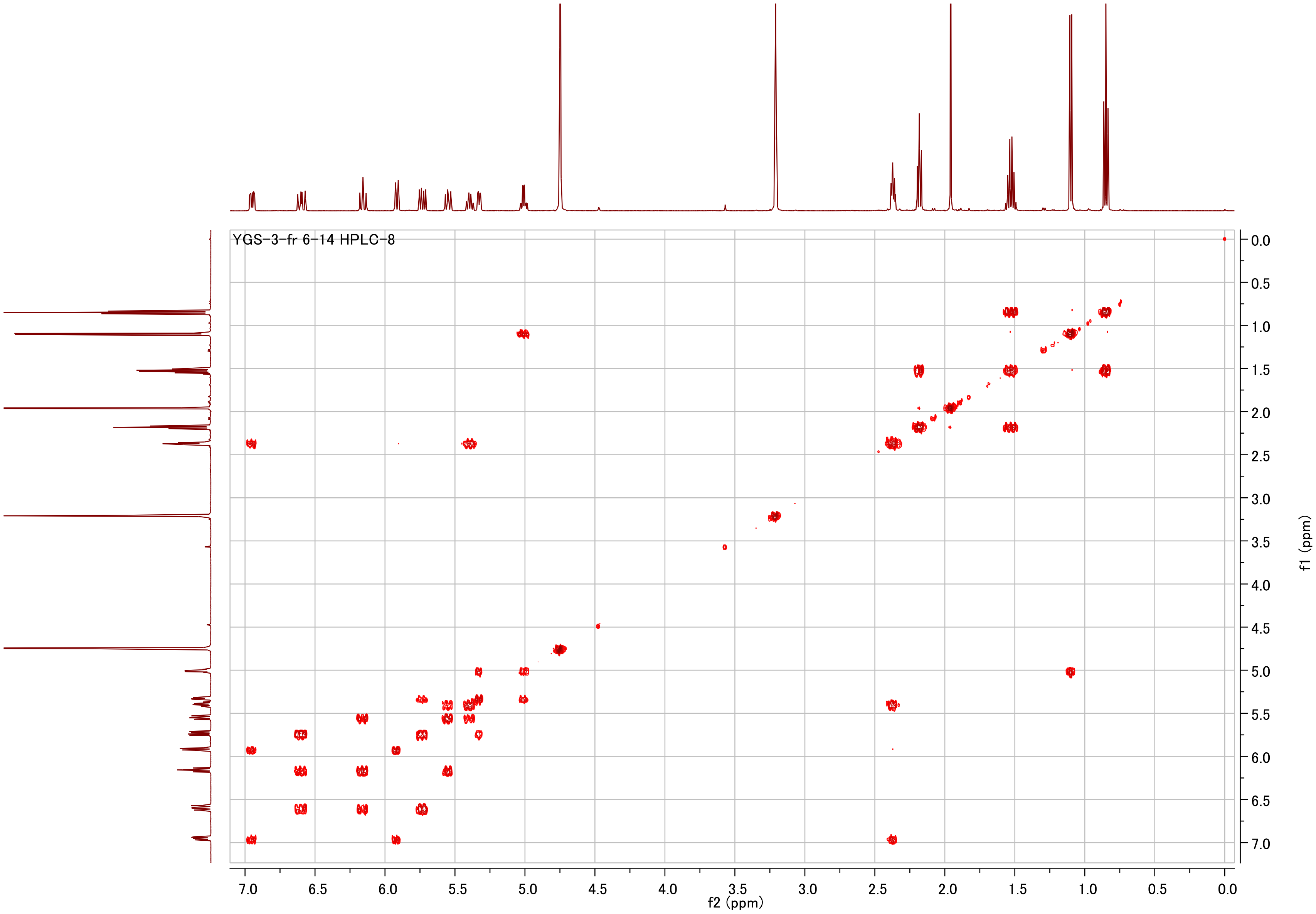

S83: ^{1}H ^{1}H COSY of **10**

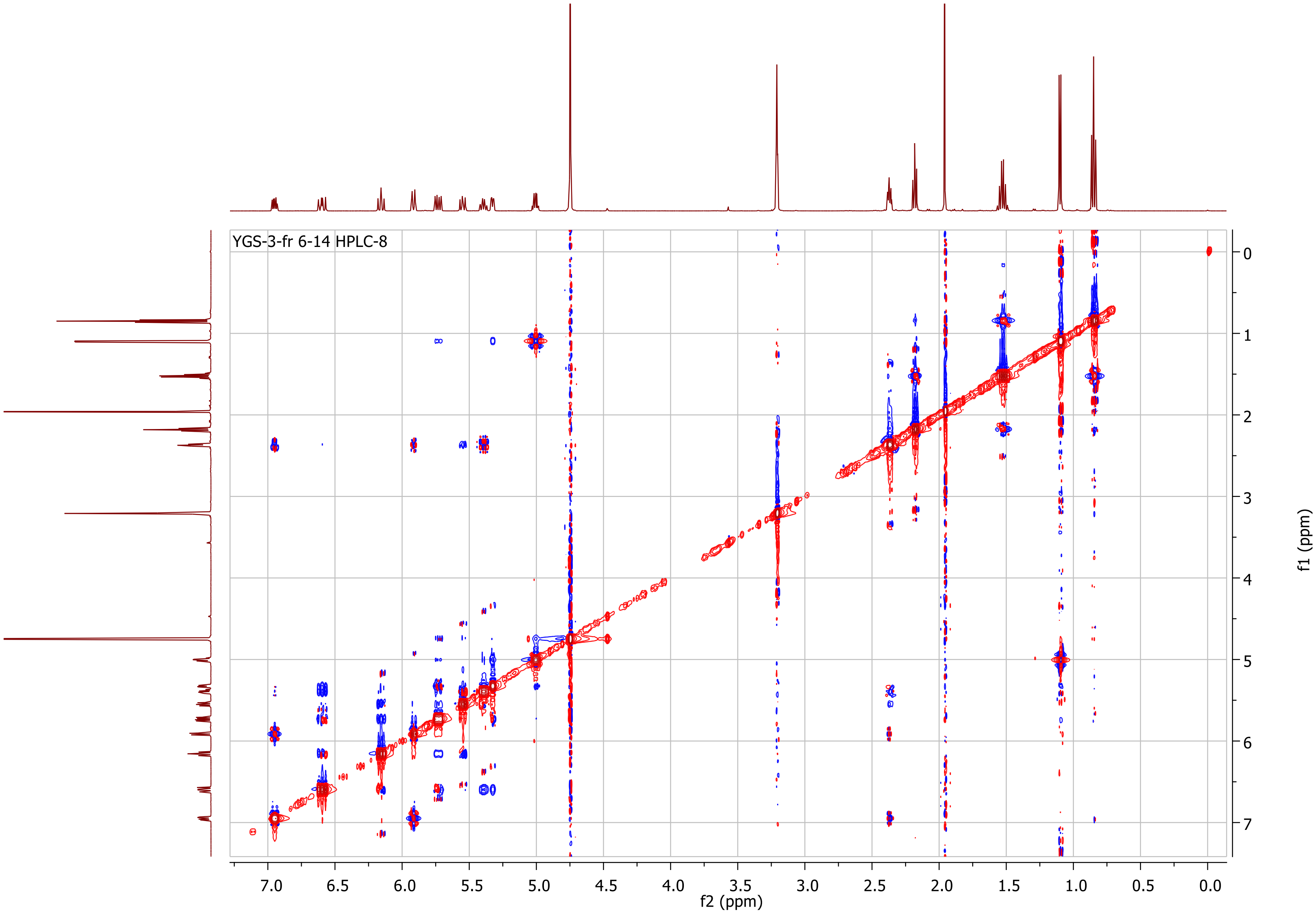

S84: NOESY of **10**

[Mass Spectrum]
Data : Umeyama-CI.22-Feb-2018.001 Date : 22-Feb-2018 10:37
Sample : YGS-6-5A(CH4)
Note : MStation
Inlet : Direct Ion Mode : CI+
Spectrum Type : Normal Ion [MF-Linear]
RT : 1.15 min Scan# : 43
BP : m/z 337 Int. : 399.99 (4194240)
Output m/z range : 35 to 500 Cut Level : 0.00 %

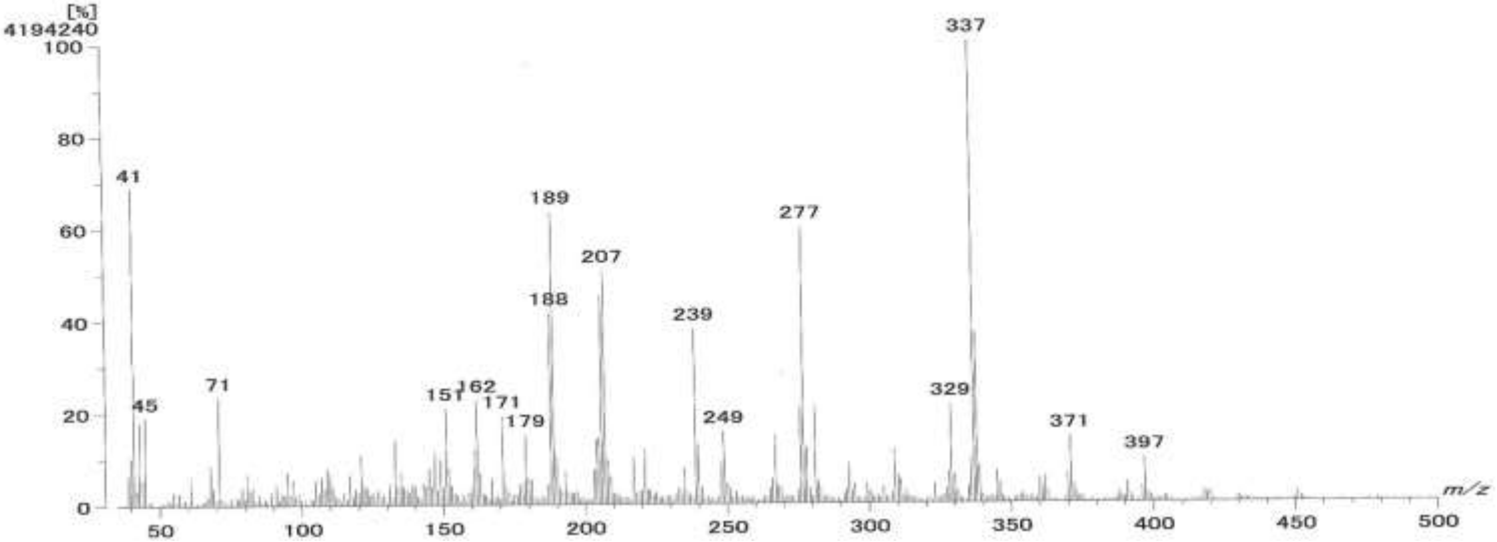

S85: LRCIMS of **11**

Data : Umeyama-CIHR.22-Feb-2018.003 Date : 22-Feb-2018 19:13
Instrument : MStation
Sample : YGS-6-5A
Note : MStation
Inlet : Direct Ion Mode : CI+
RT : 0.54 min Scan# : 15
Elements : C 150/0, H 250/0, O 50/0
Mass Tolerance : 5mmu
Unsaturation (U.S.) : 0.0 – 15.0

	Observed m/z	Int%	Err[ppm / mmu]	U.S.	Composition
1	397.1871	2.06	+2.2 / +0.9	6.5	C20 H29 O8

S86: HRCIMS of **11**

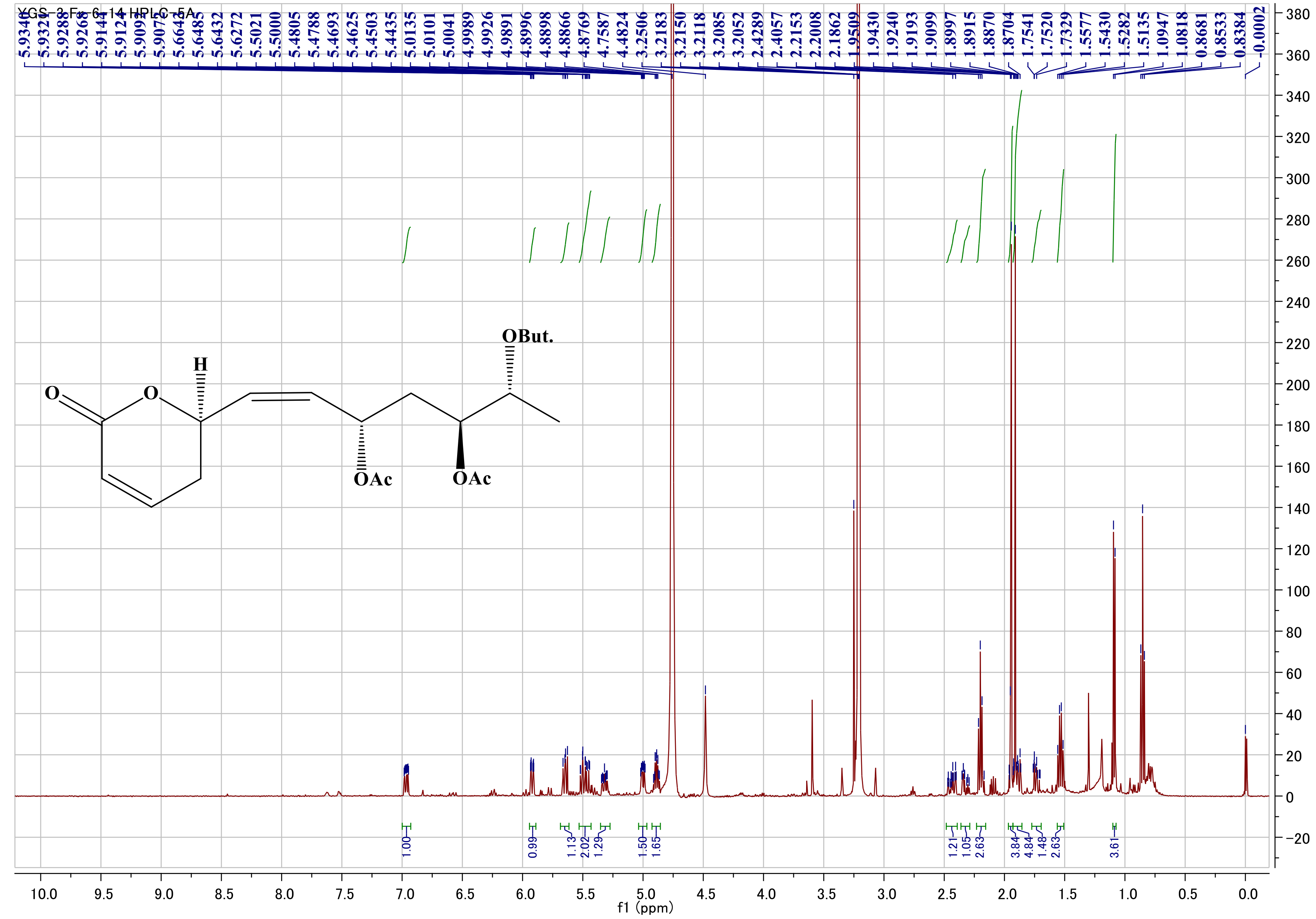

S87: ^{1}H NMR of **11**

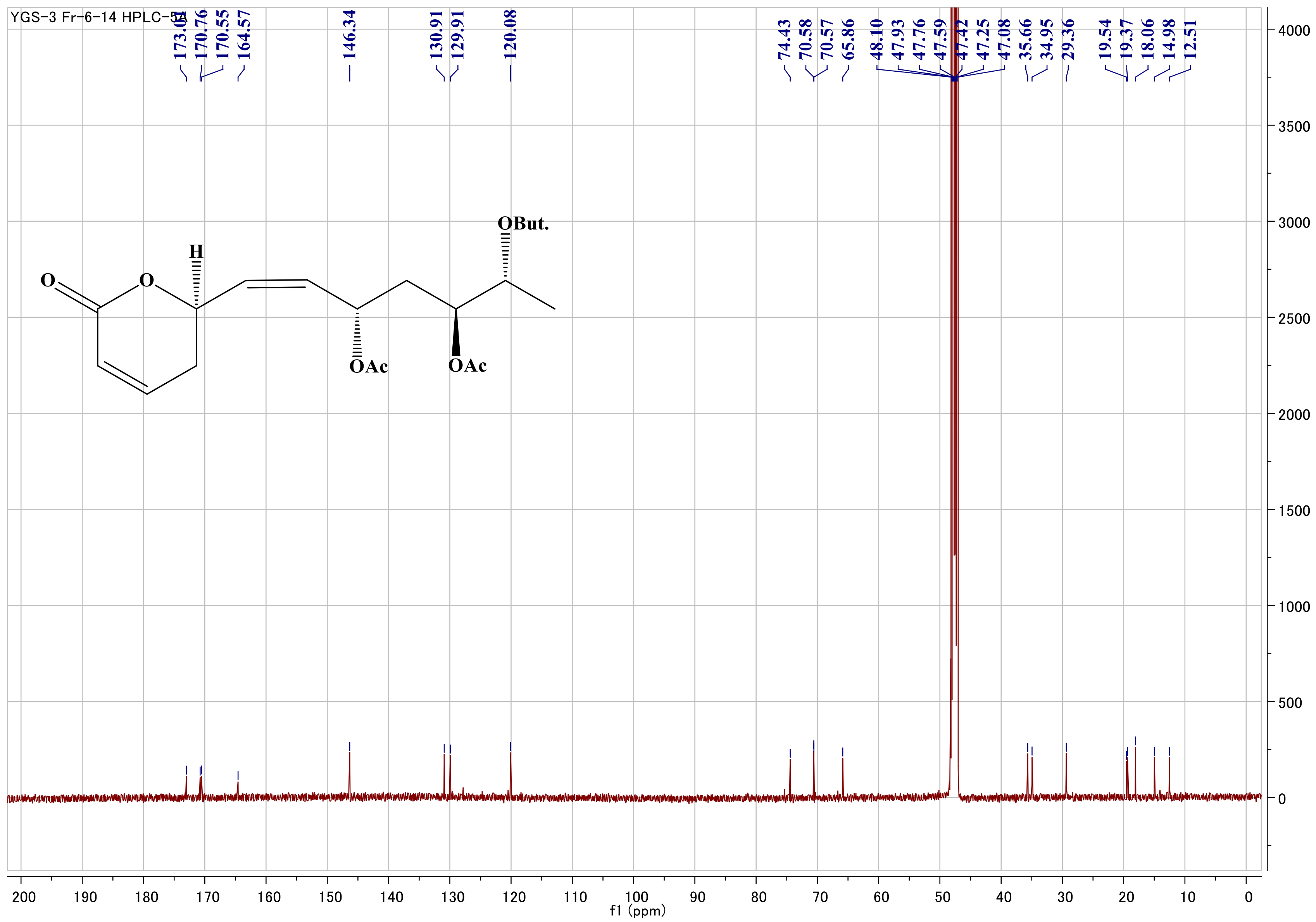

S88: ^{13}C NMR of **11**

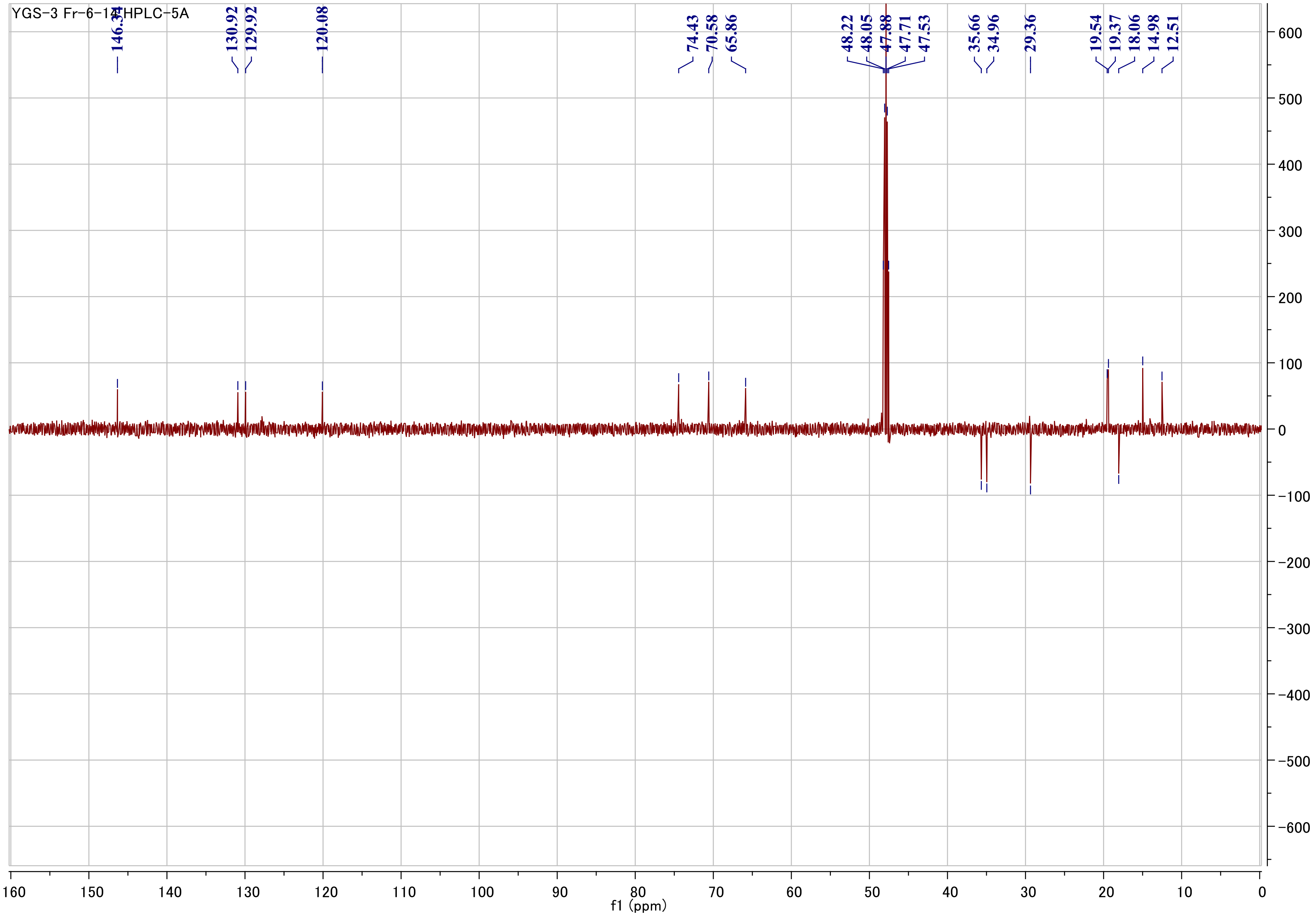

S89: DEPT-135 of **11**

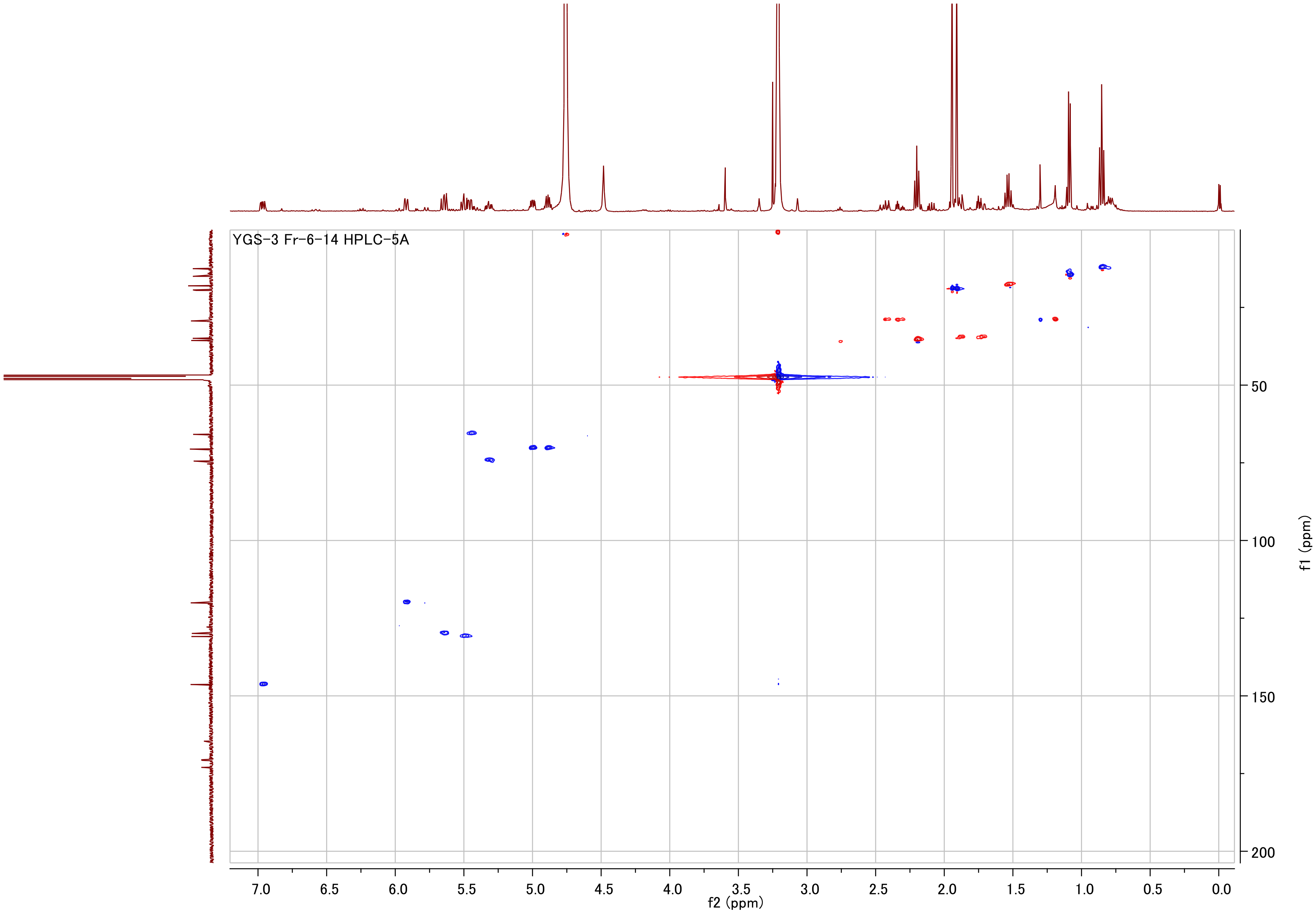

S90: HSQC of **11**

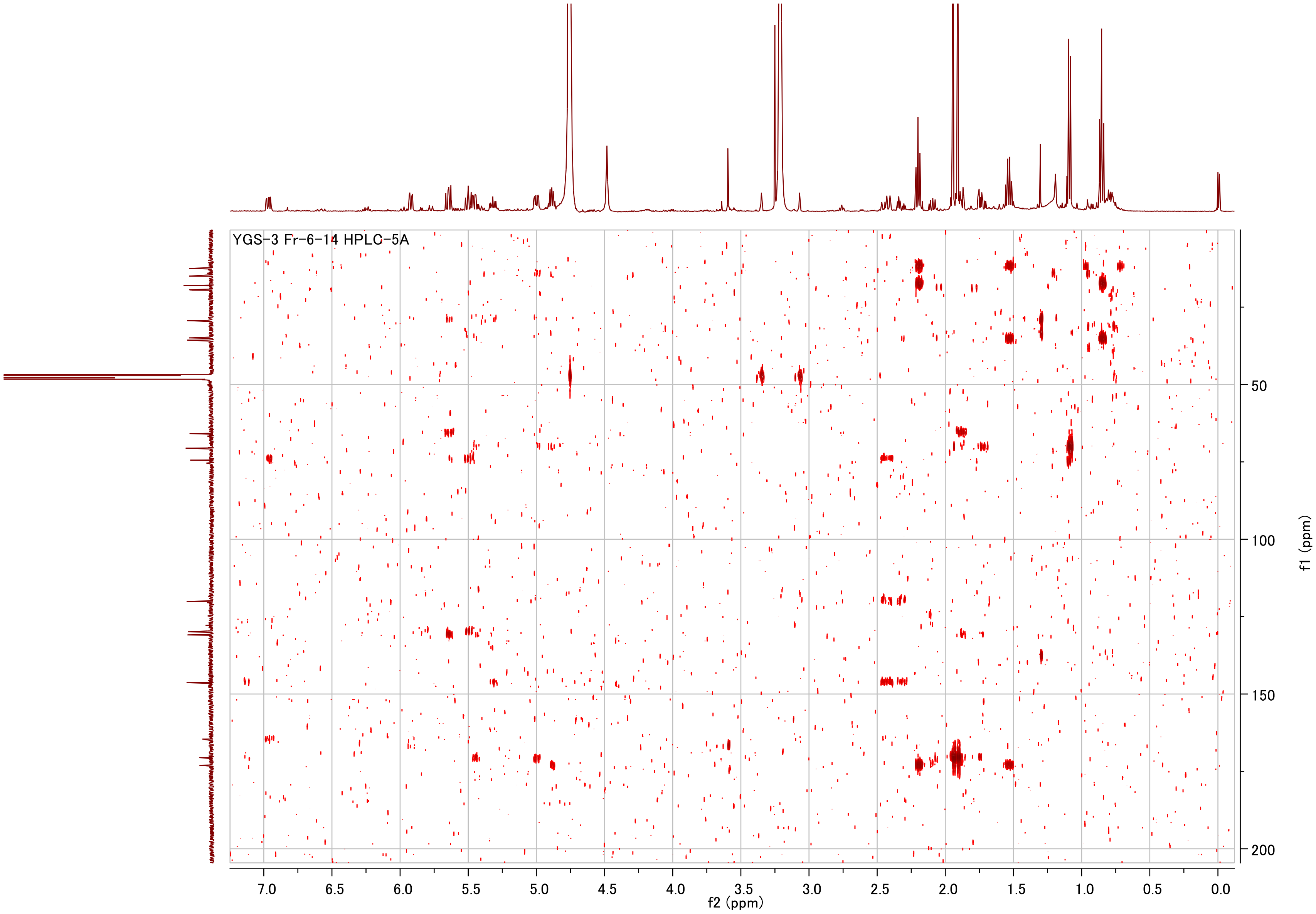

S91: HMBC of **11**

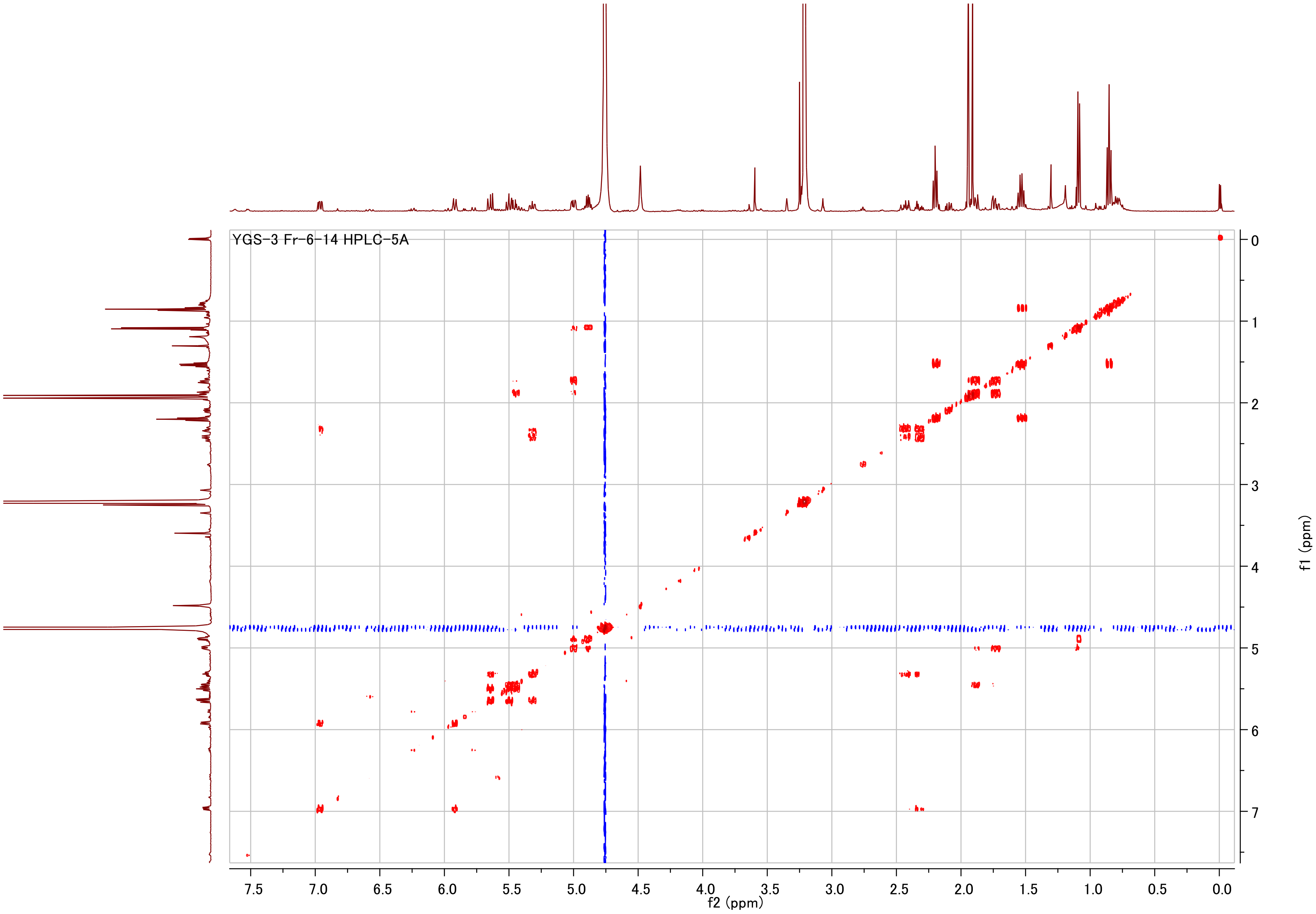

S92: ^{1}H ^{1}H COSY of **11**

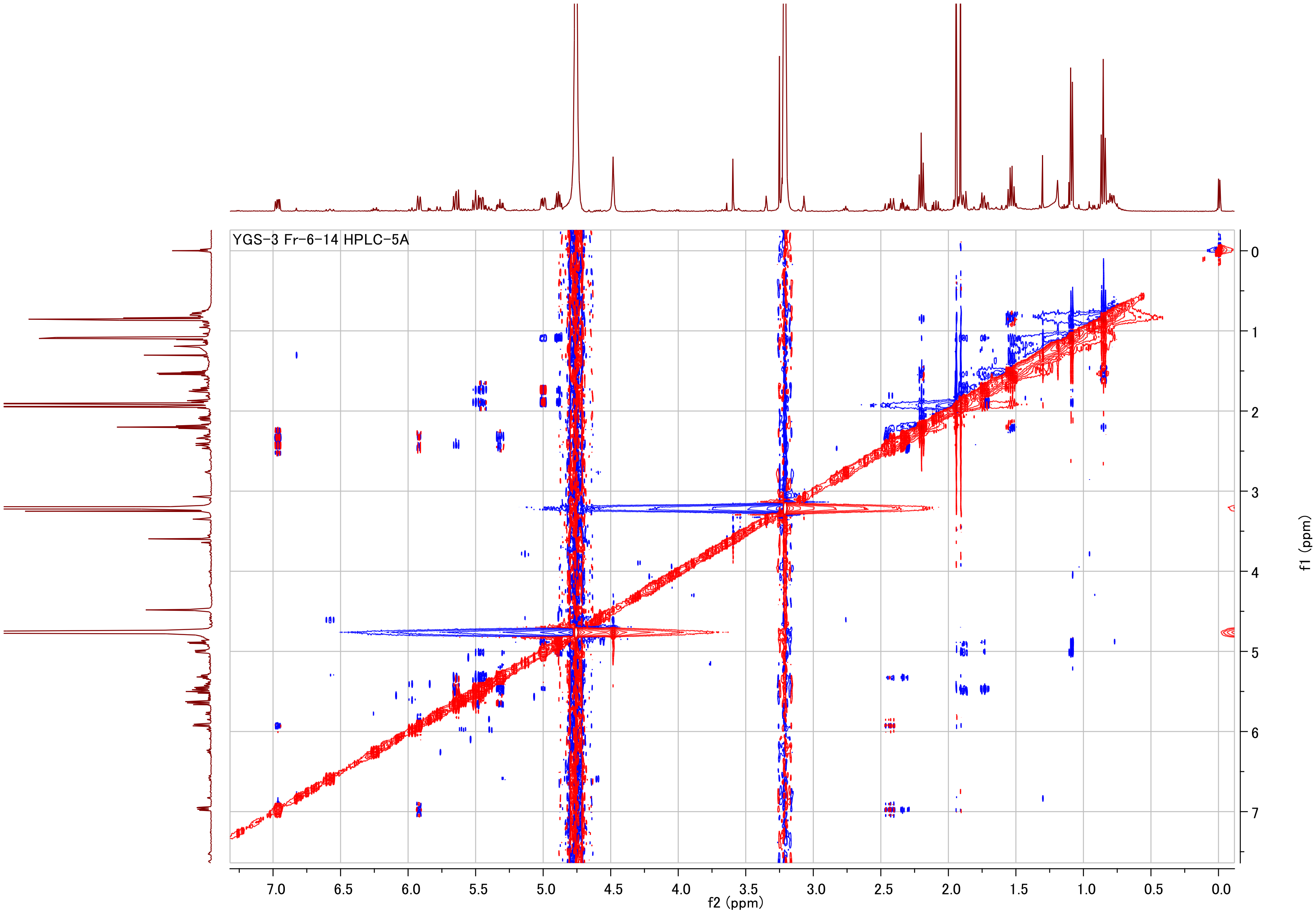

S93: NOESY of **11**

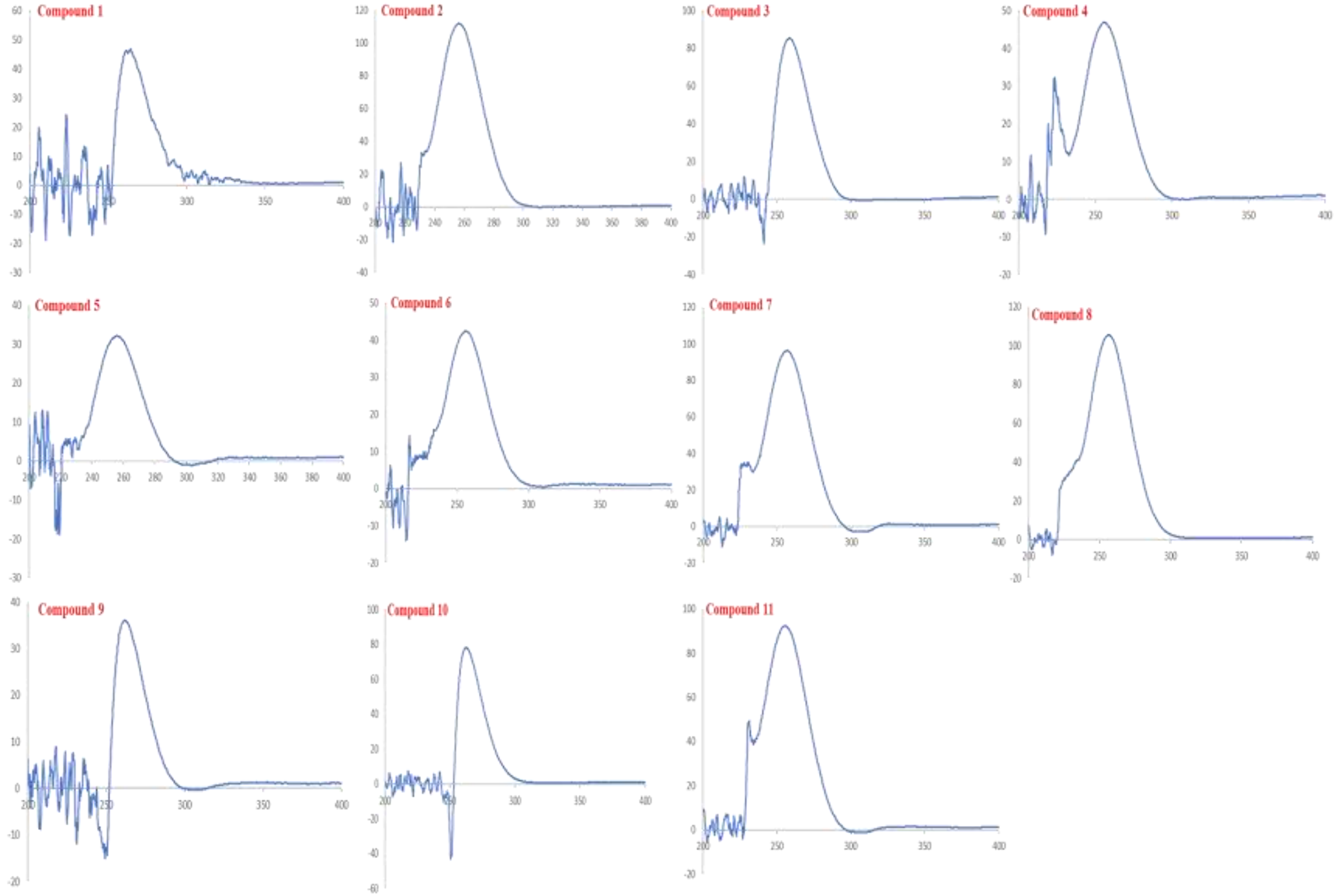

S94: Experimental ECD of isolates **1-11**
